# The Impact of Modifying Food Service Practices in Secondary Schools Providing a Routine Meal Service on Student’s Food Behaviours, Health and Dining Experience: A Systematic Review and Meta-Analysis

**DOI:** 10.3390/nu14173640

**Published:** 2022-09-02

**Authors:** Edwina Mingay, Melissa Hart, Serene Yoong, Kerrin Palazzi, Ellie D’Arcy, Kirrilly M. Pursey, Alexis Hure

**Affiliations:** 1 School of Medicine and Public Health, University of Newcastle, Newcastle, NSW 2308, Australia; 2 Hunter Medical Research Institute, New Lambton Heights, Newcastle, NSW 2305, Australia; 3 School of Health Sciences, University of Newcastle, Newcastle, NSW 2308, Australia; 4 Hunter New England Mental Health Service, Calvary Mater Hospital, Newcastle, NSW 2298, Australia; 5 Hunter New England Population Health, Hunter New England Local Health District, Newcastle, NSW 2287, Australia; 6 School of Health Sciences, Swinburne University of Technology, Melbourne, VIC 3122, Australia

**Keywords:** school meals, school-based, food service, food behaviour, dining experience, nutrition, adolescent, intervention

## Abstract

The education sector is recognised as an ideal platform to promote good nutrition and decision making around food and eating. Examining adolescents in this setting is important because of the unique features of adolescence compared to younger childhood. This systematic review and meta-analysis examine interventions in secondary schools that provide a routine meal service and the impact on adolescents’ food behaviours, health and dining experience in this setting. The review was guided by Preferred Reporting Items for Systematic Reviews and Meta-Analyses (PRISMA) Checklist and Cochrane Handbook recommendations. Studies published in English searched in four databases and a hand search yielded 42 interventions in 35 studies. Risk of bias was assessed independently by two reviewers. Interventions were classified using the NOURISHING framework, and their impact analysed using meta-analysis, vote-counting synthesis or narrative summary. The meta-analysis showed an improvement in students selecting vegetables (odds ratio (OR): 1.39; 1.12 to 1.23; *p* = 0.002), fruit serves selected (mean difference (MD): 0.09; 0.09 to 0.09; *p* < 0.001) and consumed (MD: 0.10; 0.04 to 0.15; *p* < 0.001), and vegetable serves consumed (MD: 0.06; 0.01 to 0.10; *p* = 0.024). Vote-counting showed a positive impact for most interventions that measured selection (15 of 25; 41% to 77%; *p* = 0.002) and consumption (14 of 24; 39% to 76%; *p* = 0.013) of a meal component. Interventions that integrate improving menu quality, assess palatability, accessibility of healthier options, and student engagement can enhance success. These results should be interpreted with caution as most studies were not methodologically strong and at higher risk of bias. There is a need for higher quality pragmatic trials, strategies to build and measure sustained change, and evaluation of end-user attitudes and perceptions towards intervention components and implementation for greater insight into intervention success and future directions (PROSPERO registration: CRD42020167133).

## 1. Introduction

Globally in 2017, 11 million deaths and 255 million disability adjusted life years were attributable to dietary risk factors, in particular diets high in sodium, and low in fruit, wholegrains, nuts and seeds, vegetables, and seafood omega-3 fatty acids [1]. Eating behaviours track from childhood and adolescence to adulthood and across generations [2,3,4,5,6,7,8,9], and a higher intake of fruit and vegetables is protective against burden of disease [10]. Evidence from the United States (US), Australia and the United Kingdom shows fruit intake declines with age from childhood to adolescence [11,12,13]. Vegetable intake remains alarmingly low in adolescents. For example, in the US in 2017, only 2% of high school students met the national vegetable consumption guidelines [14], and in Australia in 2017–2018, 3.8% of adolescents aged 10–14 met the recommended intake [12]. Global patterns of adolescent dietary intake have limitations related to data quality and comparability [15,16]. However, the effect of the nutrition transition towards less healthy eating patterns over the past 30 years is evident from the increased global levels of overweight and obesity in young people (10–24 years); an increase from 147.2 million in 1990 to 324.1 million in 2016 [17].

Adolescence is defined as the life stage that sits between childhood and adulthood and represents the ages from 10 to 19 years (consistent with World Health Organization (WHO, Geneva, Switzerland) definition) [18]. The population of adolescents worldwide has reached 1.2 billion representing more than 18% of the total population [19]. This is the largest cohort of adolescents in history [19] and they are developing and navigating adolescence during substantial global change [15,20]. Changes include urbanisation, globalisation of food and food environments, social and lifestyle changes, and the evolution and globalisation of all forms of media and communication [21]. These are all factors known to influence adolescents’ decision making around food and eating, and therefore their nutritional intake, health and wellbeing [15,20]. Adolescence in the public health arena has been underserved, and often neglected [22,23]. The Lancet Commission on Adolescent Health and Wellbeing (2016), and more recently the Lancet Series on Adolescent Health (2022), call for investment and targeted focus on this group of emerging adults for their health now, their adult life, and for the next generation of children [15,20].

The education sector has long been a key domain to reach adolescents and influence health behaviours [24]. United Nations Educational, Scientific and Cultural Organization (UNESCO, Paris, France) and the WHO are calling for all countries to make every school a health-promoting school [25]. The school food environment is one component of this, and refers to ‘all the spaces, infrastructure and conditions inside and around the school premises where food is available, obtained, purchased and/or consumed (for example tuck shops, kiosks, canteens, food vendors, vending machines); also taking into account the nutritional content of these foods. The environment also includes all of the information available, promotion (marketing, advertisements, branding, food labels, packages, promotions, etc.) and the pricing of foods and food products’ [26]. This environment connects several influential factors around food and eating; the context of eating with peers and their influence on food choice and eating behaviour, the preparation and presentation of food offerings, and health messaging which has the potential to contribute significantly to students’ dining experience and nutritional intake.

Boarding schools and school meal programs, as part of a school food environment, provide a daily routine meal service that may include breakfast, lunch, and/or dinner. Each main meal per day provides a significant proportion of an individual’s daily intake, and therefore influences overall diet quality. For example, boarding schools may provide all meals to students during a school year. In the United Kingdom there are approximately 65,000 boarder students (0.7% of all school students) [27,28], in Australia 23,000 boarder students (0.6% of all school students) [29], and in the US 35,000 boarder students [30]. With regard to school meal programs, in the US in 2018, 58% of students participated in the National School Lunch Program (NSLP; 29.7 million students) and 30% in the School Breakfast Program (14.7 million students) [31]. Studies indicate that students who participate in both programs consume up to 47% of their daily energy intake from school meals [32,33]. In France, a school lunch program is offered to all students, and according to the July 2006 Individual and National Study on Food Consumption (INCA2), 64% of middle and high school students eat lunch at school at least three times a week [34]. Finland, Sweden and Korea offer a free school meal to all students each day [35,36,37], and in Japan, it is mandatory for students to participate in the school meal program which is not free of charge, however low-income families receive financial support for the service [38]. Therefore, routine school meals provide a unique opportunity to reach large numbers of adolescents and influence their diet quality, food behaviours and dining experience. However, they also present challenges and barriers, such as (1) food waste, (2) palatability of food, (3) budgetary constraints, (4) low participation rates, (5) nutrition standards and regulations, and (6) availability of competitive foods, often nutritionally poor, from alternative menu offerings or vending machines [39,40]. 

A recent umbrella review of systematic reviews (reviews published from 2010 onwards) assessed the effectiveness of randomised controlled trials of school-based nutrition interventions on students (aged 6–18 years) dietary intake [41]. While this was restricted to outcomes that measured consumption, and included both primary and secondary school students, the review found that interventions can have a positive effect on fruit, vegetable and fat intake, highlighting the significance of the school food environment as a setting for such interventions. Another systematic review evaluated the potential benefits of universal free school meals (i.e., free meals to all participating students), found mostly positive associations with diet quality or food security (*n* = 4 of 7 studies conducted in the US, and *n* = 15 of 19 studies in other countries that are members of the Organization for Economic Cooperation and Development (OECD, Paris, France)), and academic performance [42]. This form of meal provision offered more broadly across the school student body, without competing lunch meal offerings (e.g., a la carte), enables a nutritious meal for all, thereby reducing diet-related disparities [42,43,44]. Other recent reviews have evaluated interventions targeting food environments in varying settings: (1) across a range of school types [39,45,46,47,48,49], or (2) including universities or other adult or all-age settings [50,51], or (3) schools restricted to the US [39], or (4) specific full service settings such as military [52] and restaurants [53]. 

A key gap in existing literature is a systematic review and meta-analysis of nutrition interventions within secondary school dining rooms evaluating adolescent food behaviours specifically in that context rather than habitual (within and outside school) behaviours. This is important to address because the school dining room is a place where students congregate to consume meals together in the same dining room, and from the same food service. This repeated exposure therefore has the potential to contribute significantly to overall intake, perhaps only second to the home environment [54]. Importantly, evaluation of nutrition interventions in the school dining room, and student food behaviours specifically in this setting, can inform the design and development of future school food service policy and practice. Therefore, the aim of the current review was to examine nutrition interventions within secondary school dining rooms that provide a routine meal service; the types of intervention strategies implemented and outcomes measured, and the impact on adolescents’ food behaviours, health and dining experience specifically within this setting. In this instance, a routine meal service is defined as one that provides a default main meal (rather than an optional purchase) to most students on most days (excluding weekends).

## 2. Materials and Methods

The current review was undertaken consistent with best practice recommendations outlined in Cochrane Handbook for Systematic Reviews of Interventions [55], and reported in accordance with the Preferred Reporting Items for Systematic Reviews and Meta-Analyses (PRISMA) 2020 statement and checklist [56]. The protocol was prospectively registered with PROSPERO (number: CRD42020167133). This paper presents the findings for students aged 10–19 attending secondary schools.

### 2.1. Search Strategies

A Population Intervention Comparison Outcome Study design (PICOS) model was used to develop the research question and database search strategy. The search strategy was developed in consultation with a senior research librarian from the University of Newcastle and tested in MEDLINE (E.M.) to verify that relevant articles from preliminary searches were retrieved. It was reviewed by authors with experience in the setting (A.H., S.Y.) and adapted for use in other databases including EMBASE, CINAHL and PsycINFO according to their phrase searching and truncation guidelines. Search terms were based on three search sets: (1) institutional setting, AND (2) intervention, AND (3) study type. Searches were restricted to English language and included all studies published up to 7 May 2020. Full search strategies for all databases included keywords and subject headings relevant to the research question to capture a broad range of nutrition-related interventions implemented within secondary schools that provide a routine meal service. Other relevant sources were hand searched (E.M.) to identify additional studies published up to December 2021, including the reference list of included articles, authors of interest, and systematic reviews of school food environment interventions [39,45,46,47,48,49,50,53,57,58,59,60,61,62,63]. 

### 2.2. Study Selection

All records retrieved were imported to Endnote X9.3.3 [64] for record management, and COVIDence software platform for double screening [65]. Initial screening of the title, abstract and citation was conducted by two reviewers independently (E.M., S.Y., E.D., K.M.P.) and allocated an ‘exclude reason’ based on a hierarchical approach: (1) study design or publication type, (2) population, (3) intervention, or (4) outcome. Full-text articles retrieved were screened independently by two reviewers (E.M., S.Y., E.D., K.M.P.) to assess eligibility. Discrepancies at each stage were resolved through discussion between reviewers or a third reviewer (A.H.).

### 2.3. Eligibility Criteria

The PICOS model was used to develop and tailor inclusion and exclusion criteria (Table 1). Randomised and non-randomised experimental trials with or without a control group (includes no intervention or comparing an intervention), and single group before-after studies were considered for this review. Setting-based public health interventions are often evaluated by clusters (i.e., groups such as schools), and we used Schmidt (2017) to categorise study designs for cluster-level interventions as either cluster randomised trial (C-RT), cluster non-randomised trial (C-NRT), controlled before-after study (CBA), and a before-after study without control (BA) [66]. In addition, we used the Cochrane Handbook to classify other studies as a non-randomised trial (NRT) when groups being compared were allocated based on methods outside the control of investigators, such as the allocation of groups according to the natural course of people’s choice [67]. C-NRT, CBA, BA and NRT studies were included to better address the review questions and PICOS criteria as only a small number of randomised trials are available, or likely to be available in such setting-based interventions [67].

Interventions were required to be implemented in a school-based setting and focused on modifying the practices of the schools’ routine meal service. For example, schools that provide a daily meal program to students, or boarding schools rather than canteen purchases over the counter. For this review, the meals needed to be provided to most students (≥50%), rather than optionally to target groups, to capture those students who are repeatedly exposed to the intervention. At <50% participation our assumption is that most students are consuming their dietary intake outside of that routine meal service, and therefore changes to the food service would have less impact. To meet the population criteria, a study had to clearly indicate students’ level of participation (i.e., text indicating most students participated or a given participation rate of ≥50% of enrolled students). If not clearly indicated, assumptions were made for relevant interventions in countries where school meal programs are known to be provided to most students: (1) Finland, where all students attending primary or secondary school are entitled to a free daily school meal [37], (2) Sweden, where a free daily school meal is offered to all students aged 7–16 years and to most aged 16–19 years [68], (3) France, where 64% of middle and high school students eat a school lunch at least three times per week [34], and (4) US, where 95% of all schools and 58% of enrolled students participate in the NSLP [31,69]. Studies were restricted to those undertaken in upper-middle- and high-income countries as defined by the World Bank Group [70] to reduce heterogeneity and increase generalisability among schools in these countries with similar nutrition governance and investment in meal services at school. Future reviews should address the needs of low-and lower-middle income countries due to the increasing prevalence of school feeding programs [43].

### 2.4. Data Extraction and Management

A data collection form was adapted from Cochrane Handbook recommendations [71] and the following extracted by the first author (E.M.) and checked by at least one other author (A.H., M.H., E.D.): study design, setting, duration and purpose, student details, inclusion and exclusion criteria, intervention details and duration, analytical methods, range of outcomes examined related to a meal component (this included a food item, food group or nutrient) or school meal program participation rate or other student measures related to the meal service, measurement tools and tool scoring, key findings and limitations. If a study included both relevant and irrelevant data components, according to the eligibility criteria, only relevant data was extracted for analysis and reporting. For example, where a study included both elementary and middle schools, and outcomes reported separately by school type, only middle school data was extracted for analysis. Study data were tabulated and managed in Microsoft Excel version 2206 (Microsoft Corporation, Redmond, WA, USA); data for the meta-analysis were managed using REDCap version 12.5.5 (Research Electronic Data Capture; Vanderbilt University, Nashville, TN, USA) tools [72,73] hosted at Hunter Medical Research Institute, Newcastle, NSW, Australia.

### 2.5. Risk of Bias and Quality Criteria

Studies included in this review were assessed for risk of bias and quality by two authors independently (E.M., A.H., M.H., E.D., K.P.) using the Quality Criteria Checklist (QCC) for primary research according to the Academy of Nutrition and Dietetics Evidence Analysis Manual [74]. This critical appraisal tool allows assessment of multiple study designs and identifies sub-questions that are the most important quality considerations for each study design. Each relevant study was rated on validity (10 questions) for the scientific soundness of the investigation, assigning each question as ‘yes’, ‘no’, ‘unclear’ or not applicable (NA). The most important sub-questions by study design were prioritised for each validity question according to guidelines outlined in the Evidence Analysis Manual. Overall, a study was rated as ‘positive’ when ≥6 questions (including 4 designated priority questions) were answered ‘yes’. If all 4 priority questions were not answered as ‘yes’ but ≥6 questions overall were ‘yes’ the study was rated as ‘neutral’. If ≥6 questions were answered ‘no’ the study was rated as ‘negative’ and excluded from the review.

### 2.6. Data Analysis

A narrative synthesis was adopted to classify intervention strategies and identify and group outcomes measured. To assess the impact of interventions, meta-analysis, vote-counting synthesis based on the direction of effect, or a narrative summary were performed. 

#### 2.6.1. Classification of Intervention Strategies

One author (E.M.) used the NOURISHING framework [75] to classify intervention strategies according to the frameworks’ suite of three key domains: (1) food environment, (2) food system, and (3) behaviour change communication; and accompanying ten action areas. The framework applies a socio-ecological and comprehensive approach to capture both environmental and behavioural strategies to promote healthier eating, improve dietary behaviours and optimise health [75,76], and has been used in recent reviews to classify intervention strategies [77,78]. Within this classification, and where relevant, behavioural economics theory [79] was also applied to categorise ‘nudging’ strategies to influence food decisions towards healthier choices (similar to other reviews [46,50]) according to concepts of: (1) acceptability: to address palatability and taste expectations, (2) accessibility: addresses the placement and convenience of healthier options, (3) availability: providing adequate variety of healthy options and limiting less healthy items, (4) presentation: improvements to the dining room and display of food, and (5) promotion: includes marketing strategies, activities and material.

#### 2.6.2. Grouping of Outcomes Measured

Interventions could contribute multiple outcomes, and each eligible nutrition-related outcome was categorised according to pre-specified outcome domains from a post hoc review of included studies: (1) student selection of a meal component, (2) student consumption of a meal component, (3) health status, (4) knowledge, (5) meal program participation rate, or (6) attitudes and perceptions related to changes to the meal service. Measurements of knowledge were included when education or promotion related to modifications to the meal service, thereby indirectly contributing to food behaviours in the dining room. Meal program participation rates reflect student acceptability of a meal service at the population level. Measurements assessing attitudes and perceptions allowed for additional insight into the dining experience including evaluation of the cafeteria environment, meal quality and palatability, qualitative feedback, sensory attributes or student satisfaction.

#### 2.6.3. Impact of Interventions

##### Meta-Analysis

Acceptable methods from Cochrane Handbook were applied to conduct all meta-analyses [80,81,82]. A meta-analysis was performed where possible to pool post-intervention scores of parallel arm controlled trials (randomised or non-randomised) with change-from-baseline scores (where baseline scores act as comparator) from BA studies (i.e., single group) and from the intervention arm of CBA studies (i.e., rather than a comparison of post-intervention scores between groups) [80,82]. Manipulation of data and analyses were conducted in Stata version 15 (StataCorp LLC, College Station, TX, USA) [83]. To be eligible for inclusion in a meta-analysis, at least two studies were required to report a pre-specified outcome with a common scale of measurement [80] for a meal component that was reported separately as either fruit, vegetables, milk or entrée (the primary component of a NSLP meal that contains grains, meat, vegetables and/or fruit; e.g., chicken salad sandwich, tacos or spaghetti [84]). For this analysis, pre-specified outcomes included: (1) percent of students selecting a meal component, (2) percent of serve consumed of a meal component by students, (3) mean number of serves selected per student per day, or (4) mean number of serves consumed per student per day. A ‘serve’ reflects a standardised portion; for example, a piece of whole fruit or prespecified weight or volume of fruit or vegetables. Each outcome per meal component was analysed using a separate meta-analysis model, and interventions could contribute multiple outcomes. For example, one intervention may contribute three outcomes such as percent of students selecting fruit, mean number of fruit serves selected by students per day, percent of fruit serve consumed when selected by students. For studies with multiple intervention arms, each intervention arm was treated separately for analysis using change-from-baseline scores. We hypothesise a random distribution of estimate effects for each outcome, because while outcome estimates for studies are related, there are noted differences in population characteristics, study designs, and the way outcomes are measured for each paper. Statistically, to estimate a random effect, a large enough number of studies must be combined; there is no universal recommendation for the minimum number of studies needed to perform a random effects meta-analysis [80], so we have used a cutoff of 5 or more studies. For those outcomes combining less than 5 studies, a fixed effects model was performed (which assumes a common estimate effect), however the results should be interpreted cautiously as they are not then generalisable to similar other studies. Combined estimates are presented as odds ratios (OR) and 95% confidence intervals (CI), and estimates of heterogeneity (I squared) are presented.

For continuous outcomes, where a standard deviation (SD) was not reported, alternative statistics (standard error, CI, t-statistic or *p*-value) were used to calculate a SD [82]. Where studies measured the percent of students selecting a meal component, dichotomous variables were determined for number of students that ‘selected’ or ‘did-not-select’ meal component for analysis. For both continuous and dichotomous outcomes, sample sizes were based on number of observations per study. We were not able to identify individual student level data because aggregate group level data were provided, and the number of observations during a data collection time point may represent multiple measurements per student. The meta-analysis pooled data from school cafeteria records and/or researcher direct observation of lunch trays depending on data collection methods; the former presenting a much larger sample size for analysis. In some studies, where limited sample sizes were provided, an estimate sample size was calculated where possible using reported data (e.g., from school cafeteria records: number of data collection days per arm, number of schools per arm, mean student enrolment per school, and proportion of students selecting a school lunch). Where there were sufficient clusters per arm (more than five), a design effect was applied to estimates assuming an intra-cluster correlation coefficient (ICC) of 0.05 [81]. We have chosen to ignore a clustering effect when there were five or fewer schools per arm because there are not enough clusters to account for within-cluster correlation. Where studies measured more than one change-from-baseline time point following intervention implementation, the meta-analysis prioritised mean values measured during the intervention period rather than follow-up measurements after the intervention ceased.

##### Vote-Counting Based on the Direction of Effect

Because numerous studies could not be included in the meta-analysis modelling (due to limited information about effect estimates, sample size, or without one of the pre-specified outcomes for meta-analysis), vote-counting based on the direction of effect method was used to synthesise results of all included studies that provided information on direction of effect [85]. Manipulation of data and analyses were conducted in Microsoft Excel version 2206 (Microsoft Corporation, Redmond, WA, USA). Vote-counting based on direction of effect and preparation of the effect direction plot followed Cochrane Handbook guidelines and methods according to Boon and Thomson (2021) [85,86]. The direction of effect (where provided) of each eligible outcome was recorded as either a positive impact (favouring intervention), negative impact (favouring comparator) or no change. Similar outcomes were combined into pre-specified outcome domains. For studies with multiple outcomes within a given outcome domain, a direction of effect was reported where a clear majority (≥70%) of outcomes reported similar direction (i.e., either positive or negative). If <70%, direction of effect was reported as no change or mixed effects [86]. For studies with multiple outcomes that included whole food items alongside measurements of their macro-and micronutrient parts, a sensitivity analysis was conducted to exclude measures that would overinflate the vote count. A sign test was performed for each outcome domain using the count of positive and negative effects (excluding no change/mixed effects) to determine any evidence of effect along with a 95% CI estimation for binomial proportions using the Wilson interval method [85,86,87]. For studies that incorporated change from baseline scores (controlled or uncontrolled), within-group results (i.e., before and after measurements) were prioritised for inclusion in analysis, otherwise post-intervention scores were used for parallel arm or crossover trials. To assess the robustness of synthesised results, a post hoc sensitivity analysis was conducted for outcome domains apportioning variables for study quality, study design, intervention duration, number of NOURISHING framework domains or action areas, student engagement, and behaviour change communication strategies that include promotional activities or student and/or staff training.

##### Narrative Summary

A narrative summary of results is provided for studies or specific outcomes in studies that were not eligible for inclusion in the meta-analysis or vote counting synthesis. Due to the range of intervention strategies in the school dining room, a post hoc analysis of intervention components warranting closer examination is also provided.

## 3. Results

Our results highlight a range of study designs and intervention strategies that were implemented in the school dining room setting. While the selection and consumption of meal components were the most frequently measured outcomes, measurement of attitudes and perceptions related to the changes to the meal service provide useful insight into student experiences and intervention success. To assess the impact of interventions, the meta-analysis, vote-counting and narrative synthesis found no trend associated with study design or quality. However, the assessment did highlight the importance of the school food environment as an ideal platform to improve nutrition by uncovering trends associated with certain intervention components according to the NOURISHING frameworks’ suite of domains and action areas.

### 3.1. Search Results

**Figure 1:**
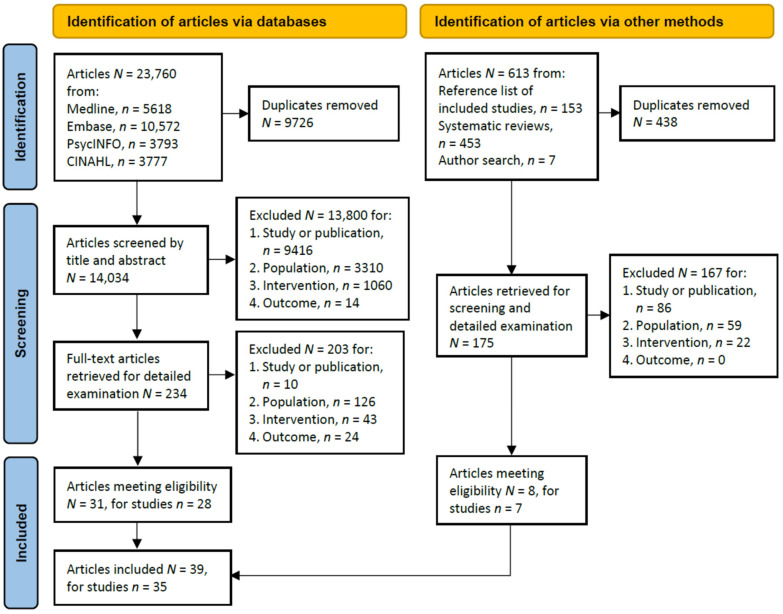
Preferred Reporting Items for Systematic Reviews and Meta-Analyses (PRISMA) 2020 flow diagram to illustrate selection of included studies for systematic review.

Figure 1 illustrates a PRISMA flowchart of the study selection process. From all sources, 35 studies reported in 39 peer-reviewed articles were included for synthesis.

### 3.2. General Characteristics of Included Studies

Table 2 presents study characteristics and intervention components classified according to the NOURISHING framework’s domains, and corresponding details of strategies implemented. Included studies were conducted predominately in the US (31 of 35, 88.6%). Two studies were conducted in the United Kingdom, one in Sweden and one in France. Study designs included C-RTs (*n* = 4, 11%), C-NRTs (*n* = 3, 9%), NRTs (*n* = 5, 14%), CBA (*n* = 7, 20%) and BA studies (*n* = 16, 46%). Over one-third of the studies were pilot studies (13 of 35, 37%; 2 of these were randomised). All studies were pragmatic experimental trials conducted in real-life routine practice conditions within the dining room of secondary schools (includes middle and high schools; students aged 10–19 years). Publication dates ranged from 1985 to 2021. Duration of interventions ranged from one day to two years.

### 3.3. Risk of Bias and Quality Assessment

Nine studies (26%) were rated as ‘positive’ indicating the studies adequately addressed issues of inclusion/exclusion, bias, generalisability, data collection and analysis. The remaining 26 studies (74.3%) were rated as ‘neutral’ indicating they are neither exceptionally strong nor exceptionally weak; no studies were rated as ‘negative’. Inadequate description of handling withdrawals, lack of blinding, and missing data were the main risks of bias in those studies rated as neutral. Full quality assessments are provided (Supplementary Materials Table S1).

### 3.4. Data Analysis

#### 3.4.1. Intervention Strategies

A total of 42 interventions were implemented across 35 studies (7 studies included 2 intervention arms). Table 3 presents the classification of intervention strategies from included studies using the NOURISHING framework. Table 4 summarises the number of domains and action areas targeted per intervention. All interventions targeted at least one action area from the food environment domain; mostly the provision of healthy food in some form (i.e., policy implementation, reformulation of recipes or menus, increased availability of healthy options or reduced availability of less healthy options) and/or choice architecture to nudge students towards healthier food choices. Twenty-five interventions targeted the food system domain (60%) which included engagement with stakeholders across the food service (students and staff) and procurement of healthier ingredients, and 29 interventions targeted at least one action area from the behaviour change communication domain (69%). Overall, 22 of 42 interventions (52%) included components across all 3 domains, 10 interventions (24%) across 2 domains, and 10 interventions (24%) across 1 domain only. 

#### 3.4.2. Outcomes Measured

A detailed assessment of outcomes measured, measurement tools and scoring, results, major findings and limitations are provided (Supplementary Materials Table S2). Most interventions contributed multiple outcomes that were eligible for inclusion. Selection and/or consumption of a meal component/s were the most frequently measured outcomes (in 35 of 42 interventions, 83%). Fifteen interventions measured selection and consumption, 11 measured selection only, and 9 measured consumption only. Outcomes (categorised according to pre-specified outcome domains) included:

1. Student selection of a meal component: measured as either (i) percent of students selecting, (ii) numbers of serves selected, or (iii) amount selected using weight or fluid measurements; assessed in *n* = 26 interventions (62%) and included fruit (*n* = 17), vegetables (*n* = 18), entrée (*n* = 11), milk (*n* = 11), grains (*n* = 4), protein foods (*n* = 3), energy (*n* = 2), and desserts, overall meal, healthier foods, sides and saturated fat (each, *n* = 1).
2. Student consumption of a meal component: measured as either (i) percent of serve consumed, (ii) number of serves consumed, or (iii) amount consumed using weight of fluid measurements; assessed in *n* = 24 interventions (57%) and included measures of fruit (*n* = 14), vegetables (*n* = 15), milk (*n* = 10), entrée and energy (each, *n* = 5), protein foods and saturated fat (each, *n* = 4), grains, calcium, iron, sodium, total fat, vitamin A and vitamin C (each, *n* = 3), fibre and sodium (each, *n* = 2), overall meal, healthier foods, sides, carbohydrates, folate and zinc (each, *n* = 1).
3. Health status: Blood pressure (BP) was measured in *n* = 1 intervention to assess the impact of reduced sodium in school meals. Body mass index (BMI) was measured in *n* = 1 intervention to assess the impact of interactive kiosks to guide student lunch choices.
4. Knowledge: One study (*n* = 2 intervention arms) measured knowledge about fish before and after an intervention that aimed to increase students’ intake of fish at school and included classroom education about fish preparation in the school kitchen.
5. Meal program participation rate: assessed in *n* = 5 studies and represents the proportion of enrolled students that participated in the school meal program pre-and post-intervention, reflecting population level selection/acceptance of the school meal program without separating components of the meal program or reflect consumption.
6. Attitudes and perceptions related to changes to the meal service: assessed in *n* = 15 interventions (*n* = 13 with before and after measurements) to assess students’ attitude toward school lunch and the cafeteria, acceptability of modified or new menu items, or feedback on intervention components.

#### 3.4.3. Impact of Interventions

##### Meta-Analysis

Twelve studies of 13 interventions were eligible for inclusion in meta-analyses; four were judged to be at low risk of bias with mixed effectiveness for selection and consumption outcomes [88,89,90,91]. The number of intervention schools ranged from one to 12. Intervention duration ranged from one day to two years with no difference in effectiveness between shorter (≤2 months) compared to longer (3+ months) interventions. Intervention strategies varied and included updating nutrition standards (*n* = 2) [90,92], reformulation of recipes or menus including engagement of a professional chef (*n* = 4) [89,93,94,95], removal of competitive foods (*n* = 1) [96], nudging strategies (*n* = 5) [88,91,96,97,98], and food systems education and promotion (*n* = 1) [99]. The pooled effect of interventions is summarised here, and forest plots provided (Supplementary Materials Figures S1–S4):

1. Number of students selecting a meal component (Supplementary Materials Figure S1): Four separate meta-analyses were prepared for fruit (*n* = 7 studies), vegetables (*n* = 8 studies), entrée (*n* = 6 studies; 7 interventions) and milk (*n* = 6 studies). The pooled effect showed interventions increased the proportion of students selecting vegetables (OR: 1.39; 95% CI: 1.12, 1.73; *p* = 0.002), with no change in the proportion of students selecting fruit (OR: 1.03; 95% CI: 0.84, 1.27; *p* = 0.774), entrée (OR: 1.03; 95% CI: 1.00, 1.06; *p* = 0.076) or milk (OR: 0.96; 95% CI: 0.91, 1.01; *p* = 0.088).
2. Percent of serve consumed of a meal component by students (Supplementary Materials Figure S2a): Four separate meta-analyses were prepared for fruit (*n* = 5 studies), vegetables (*n* = 6 studies), entrée (*n* = 4 studies) and milk (*n* = 5 studies). The pooled effect found no change in the percent of serve consumed by students who selected fruit (mean difference MD: 2.99; 95% CI: −2.24, 8.21; *p* = 0.262), vegetables (MD: 8.64; 95% CI: −4.67, 21.94; *p* = 0.203), entrée (MD: 4.46; 95% CI: −0.93, 9.84; *p* = 0.105) or milk (MD: 0.88; 95% CI: −5.61, 7.36; *p* = 0.791). Supplementary Materials Figure S2b presents a sensitivity analysis excluding Wansink et al. [95] (1 day intervention measuring vegetable consumption) and an improved pooled effect for vegetables (MD: 13.69; 95% CI: 6.09, 21.28; *p* < 0.001).
3. Mean number of serves of a meal component selected per student per day (Supplementary Materials Figure S3a): Two separate meta-analyses were prepared for fruit (*n* = 4 studies) and vegetables (*n* = 4 studies). The pooled effect showed interventions increased the number of fruit serves selected per student per day (MD: 0.09; 95% CI: 0.09, 0.09; *p* < 0.001), with no change in vegetable serves selected (*p* = 0.977). The pooled estimate for both fruit and vegetable serves selected per student per day is not a good representation due to the large sample size for one study (Bogart et al., 2014 [88]; *n* = 102,262) that highly influenced the pooled estimate (weighting > 99%). A sensitivity analysis excluded this study (Supplementary Materials Figure S3b). The pooled effect of remaining three studies showed an increase in serves selected per student per day of fruit (MD: 0.14; 95% CI: 0.08, 0.20; *p* < 0.001) and vegetables (MD: 0.11; 95% CI: 0.05, 0.18; *p* = 0.001).
4. Mean number of serves of a meal component consumed per student per day (Supplementary Materials Figure S4): Two separate meta-analyses were prepared for fruit (*n* = 4 studies) and vegetables (*n* = 4 studies). The pooled effect showed interventions increased the number of serves consumed per student per day of fruit (MD: 0.10; 95% CI: 0.04, 0.15; *p* < 0.001) and vegetables (MD: 0.06; 95% CI: 0.01, 0.10; *p* = 0.024).

##### Vote Counting Based on the Direction of Effect

Forty one of 42 interventions and four outcome domains (selection and consumption of a meal component, meal program participation rate, attitudes and perceptions) were eligible for inclusion in vote counting based on direction of effect analysis; nine were judged to be at low risk of bias with either positive or mixed effects across outcome domains. Two outcome domains (health status and knowledge) were excluded because they only included one study, or outcomes within the domain were not suitable to combine. 

**Figure 2:**
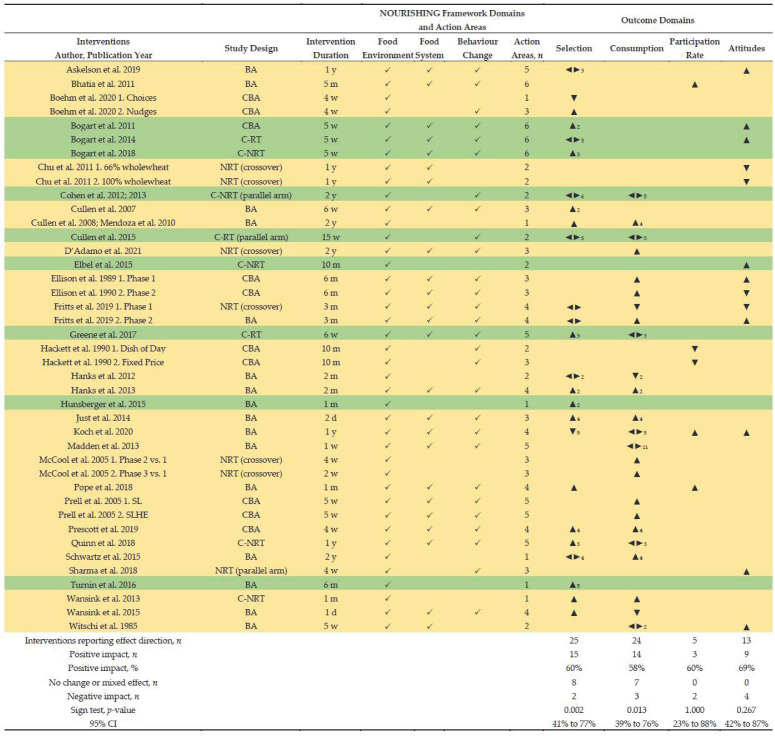
Effect direction plot summarising direction of student food behaviours in the dining room from studies that modified the practices of the routine meal service at secondary schools. **LEGEND:** Study design: C-RT, cluster randomised trial; C-NRT, cluster non-randomised trial; CBA, controlled before after study; BA, before after; NRT, non-randomised trial; studies include pre-post scores for single or multiple arm trials unless indicated as parallel arm or crossover beside study design. Study quality according to the Academy of Nutrition and Dietetics Quality Criteria Checklist [74]: denoted by row colour: green = positive rating; amber = neutral rating. Effect direction: upward arrow ▲ = positive impact, downward arrow ▼ = negative impact, sideways arrow ◄► = no change or mixed effects for multiple outcomes. Subscript numbers: Number of outcomes within each category synthesis is 1 unless indicated in subscript beside effect direction. Sign test: excludes studies with mixed effects direction as they cannot be said to represent either a positive or a negative effect direction [86]. 95% CI (confidence interval): estimation for binomial proportions using the Wilson interval method [85]. y: year/s; m: month/s; w: week/s; d: day/s. SLHE: school lunch plus home economics intervention; SL: school lunch intervention; ✓: indicates the intervention has included components from the nominated NOURISHING framework domain [44,88,89,90,91,92,93,94,95,96,97,98,99,101,103,104,105,106,107,108,109,110,111,112,113,114,115,116,117,118,119,120,121,122,123,124,125].

Figure 2 presents the effect direction plot for eligible outcome domains and includes intervention duration and components according to the NOURISHING framework’s domains and action areas. There was evidence that interventions that modified the routine meal service had an impact on (1) student selection of a meal component, with 15 of 25 interventions reporting a positive impact (60%; 95% CI 41% to 77%, *p* = 0.002), 2 negative, and 8 mixed effect (32%), and (2) student consumption of a meal component, with 14 of 24 interventions reporting a positive impact (58%; 95% CI: 39% to 76%, *p* = 0.013), 3 negative, and 7 mixed effect (29%), and (3) meal program participation rate, with 3 of 5 interventions reporting a positive impact (60%; 95% CI: 23% to 88%) and 2 negative, and (4) attitudes and perceptions related to changes to the meal service, with 9 of 13 interventions reporting a positive impact (69%; 95% CI: 42% to 87%, *p* = 0.267) and 4 negative. Nine interventions were judged to be at low risk of bias; 5 of 8 (63%) favoured the intervention for selection of a meal component, 3 of 3 (100%) showed mixed effects for consumption of a meal component, and 3 of 3 (100%) favoured the intervention for student attitudes and perceptions.

Table 5 presents results of sensitivity analysis apportioning variables for study quality, study design, intervention duration, number of NOURISHING frameworks’ domains and action areas, student engagement, and behaviour change communication strategies. Effect direction plots for each are provided (Supplementary Materials Figures S5–S11). For the selection and consumption of a meal component, notable findings include:

1. Intervention duration: there was evidence that shorter interventions (≤2 months) had greater impact on selection and consumption of a meal component compared to longer interventions (≥3 months): selection, 12 of 15 short interventions favoured the intervention (80%; 95% CI: 55% to 93%, *p* = 0.003) compared to 3 of 10 longer interventions (30%; 95% CI: 11% to 60%, *p* = 0.625); consumption, 8 of 13 short interventions favoured the intervention (62%; 95% CI: 36% to 82%, *p* = 0.109) compared to 6 of 11 longer interventions (55%; 95% CI: 28% to 79%, *p* = 0.125).
2. NOURISHING framework domains: there was evidence that interventions targeting three domains had a greater impact on selection and consumption of a meal component compared to interventions targeting less (≤2): selection, 10 of 15 targeting three domains favoured the intervention (67%; 95% CI: 42% to 85%, *p* = 0.012), compared to 5 of 10 targeting ≤2 domains (50%; 95% CI: 24% to 76%, *p* = 0.219); consumption, 9 of 15 targeting three domains favoured the intervention (60%; 95% CI: 36% to 80%, *p* = 0.065), compared to 5 of 9 targeting ≤2 domains (56%; 95% CI: 27% to 81%, *p* = 0.219).
3. NOURISHING framework action areas: there was evidence that interventions targeting more action areas (≥3) had a greater impact on selection and consumption of a meal component compared to interventions that targeted less (≤2): selection, 11 of 16 with more action areas favoured the intervention (69%; 95% CI: 44% to 86%, *p* = 0.006), compared to 4 of 9 with less (44%; 95% CI: 19% to 73%, *p* = 0.375); consumption, 11 of 17 with more action areas favoured the intervention (65%; 95% CI: 41% to 83%, *p* = 0.022), compared to 3 of 7 with fewer (43%; 95% CI: 16% to 75%, *p* = 0.625).
4. Student engagement: there was evidence that interventions that engaged students in development and/or implementation had a greater impact on selection and consumption of a meal component compared to interventions without student engagement: selection, 7 of 9 with student engagement favoured the intervention (78%; 95% CI: 45% to 94%, *p* = 0.016), compared to 8 of 16 without student engagement (50%; 95% CI: 28% to 72%, *p* = 0.109); consumption, 5 of 6 with student engagement favoured the intervention (83%; 95% CI: 44% to 97%, *p* = 0.063), compared to 9 of 18 without student engagement (50%; 95% CI: 29% to 71%, *p* = 0.146).

##### Narrative Summary

The health status outcome domain included two outcomes from two studies that were not similar to combine for analysis. Ellison et al. [100] measured BP (in addition to sodium intake). The intervention showed a significant improvement in systolic (−1.7 mmHg; 95% CI: −0.6, −0.29; *p* = 0.003) and diastolic BP (−1.5 mmHg; 95% CI: −0.6, −2.5; *p* = 0.002). The knowledge outcome domain included one study with two intervention arms [101]; both measured and significantly increased pre-to post-intervention student knowledge about fish (*p* < 0.001). Bean et al. [102] was excluded from all previous analyses due to limited data and direction of effect not reported. The impact of food service staff training on implementing behavioural economics strategies showed no change in student sales of fruit (*p* = 0.150), vegetables (*p* = 0.245), salad bar (*p* = 0.525), milk (*p* = 0.245) or water (*p* = 0.986).

All interventions targeted at least one action area from the NOURISHING frameworks’ food environment domain, and components warrant closer examination in respect to four outcome domains (selection and consumption of a meal component, meal program participation rate, attitudes and perceptions). Notable findings include the benefits associated with school-food policy implementation, increasing the availability and accessibility of healthy options, and reduced availability of less healthy options. For example, Cullen et al. [103] mandated restrictions on less healthy options and increased consumption of vegetables, fibre, vitamin A, vitamin C, calcium, sodium (all *p* < 0.025) and the percentage of energy of the lunch meal consumed from fruit, vegetables and entrée (*p* < 0.002) [104]. Schwartz et al. [92] implemented updated NSLP nutrition standards and increased vegetable and entrée consumption (*p* < 0.05). Bhatia et al. [44] improved meal program participation at all participating schools (statistical significance not assessed) after removing competitive offerings and expanding and promoting NSLP options, Boehm et al. [96] removed competitive foods and increased number of entrees served daily compared to control (*p* < 0.05), and Madden et al. [105] placed restrictions on less healthy options and increased consumption of fruit and vegetables (*p* < 0.001). The introduction of a fast service lane offering pre-plated healthy options increased service speed (*p* < 0.01) and students were satisfied with the service speed and meal quality [106]. Installation of water jets near the lunch line improved students’ water-drinking behaviours [107]. Strategic placement of healthier options [91,96,97,98,108] and pre-sliced fruit [88,91,96,98,108,109,110,111] were mostly effective in increasing selection or consumption of meal components. A novel intervention that integrated technology in the dining room for students to visualise and select a balanced meal increased the proportion of students selecting fruit and vegetables (*p* < 0.05; consumption not assessed) [112]. 

To improve the nutritional quality of meals, eight studies engaged a professional chef or dietitian to guide recipe or menu reformulation, staff training, and/or facilitate promotional events for students [89,93,94,98,105,113,114,115]. Three of these studies included student taste-testing of modified recipes; spices and herbs were added to NSLP vegetable dishes with mixed effects on consumption [113], a chef modified pizza and burger recipes and increased vegetable consumption (*p* < 0.005) [93], and new vegetable-focused entrée recipes increased selection (*p* < 0.001) [94]. Three studies included staff training to modify recipes resulting in increased consumption of vegetables (*p* < 0.01) [89], reduced consumption of sodium (*p* < 0.001) and saturated fat (statistical significance not assessed) [115,116], and improved nutritional quality of the lunch meal and increased overall fruit and vegetable consumption (*p* < 0.001) [105]. The remaining two studies engaged a dietitian to implement menu change goals and improved fruit and vegetable selection (statistical significance not assessed) [114], and as part of a team to support implementation of choice architecture strategies which had mixed results across meal components but increased consumption of fruit excluding juice (*p* < 0.05) [98].

**Table 2:** Characteristics of the studies included in the systematic review.

#	Author, Year of Publication	Study Design, Study Duration ^+^ (Dates)	Setting	Sample Characteristics	Study Aims	Intervention Duration ^+^ (Dates), Components *	Intervention Detail
1	Askelson et al., 2019 [117]	Before-after Pilot 1 y (2016)	USA, Iowa, rural and urban areas	6 middle schools (5 rural and 1 urban); 1 intervention Grades served by schools K-12; 5–8; 6–8 and 7–8 Enrolment across all schools, *n* = 3326, range *n* = 341–1140 per school; all students exposed to intervention; age NR; eligible for FRP lunch, range 18% to 42%	To improve the lunchroom environment to promote healthy food choices and empower food service staff with the knowledge, skills, and ability to communicate with students about making healthy choices in the lunchroom	1 y (2016) Food service operations BE: accessibility, availability, presentation Stakeholder engagement: staff, students BE: promotion Staff training Student training	Changing how students move through the lunch line to improve food service Offering pre-sliced fruit, re-arranging milk coolers, adding bowls, bins and stand-alone carts for whole fruit to the lunch line, adding menu boards Student lunchroom assessment conducted by students to inform nudge strategies; student groups assisted with planning and implementation of lunchroom changes; meetings between staff and students Visual cues at lunch line for staff communication prompts, food naming, table signage with menu and fruit/veg facts Webinars for food service staff including nutrition for adolescents, communication strategies Research team trained students on principles of behavioural economics and how it can be applied in the lunchroom
2	Bean et al., 2019 [102]	Before-after 2 y (2014–2016)	USA, Virginia	16 schools: 8 middle, 8 high; 1 intervention Demographic data: student sample size or age NR School district demographics: 75% African American, 13% Hispanic, 9% white, 1% Asian, 2% other ethnicity; 83% of schools with >90% NSLP participation rate	To examine the impact of food service staff training on Smarter Lunchroom adherence in school cafés	2 y (2014–2016) BE: accessibility, availability, presentation BE: promotion Staff training	Smarter Lunchroom changes: strategic placement of healthy foods, low-cost/no-cost solutions to promote healthier school lunches (varied between schools) Signage/marketing materials and suggestive selling strategies Train-the-trainer model to teach cafeteria staff Smarter Lunchroom principles to promote student healthy food selections
3	Bhatia et al., 2011 [44]	Before-after Pilot 2 y (2008–2010)	USA, San Francisco, California	3 schools: 1 middle school, 2 high schools; 1 intervention Demographic data: enrolment across all schools, *n* = 4304; student age NR	To examine the impact of removing competitive a la carte lunch offerings and providing greater diversity of meal offerings for all students, on NSLP participation rates	5 m (January–May 2010) Food service operations BE: availability, acceptability, presentation Stakeholder engagement: staff, students BE: promotion Staff training	New point-of-service system, additional staff for line control, a la carte line removed and re-purposed for NSLP A la carte options removed, expanded NSLP options, add salad bars and refrigerators, student taste testing, installation of student-designed mural, designed and posted new menus Students engaged for taste testing, surveys and mural design; staff consultation for design and implementation of initiatives Branded and marketed former a la carte locations; student taste testing Training on NSLP rules
4	Boehm et al., 2020 [96]	Controlled before-after (random allocation of schools) Pilot 9 m (September 2013–May 2014)	USA, Northeast USA, urban area	3 high schools; 2 interventions 2 I-schools: (1) Choices school, *n* = 1177 enrolled students, (2) Nudging school, *n* = 2140 enrolled students 1 C-school: *n* = 1297 enrolled students Demographics: student age NR; ethnic diversity (NS differences across schools); >95% students eligible for FRP meals, therefore free meals provided to all students	To compare federally reimbursable meals served when competitive foods are removed and when marketing and nudging strategies are used in school cafeteria operating the NSLP	4 w (April–May 2014) Choices school: food service operations Choices school, BE: availability Nudging school, BE: accessibility, availability, presentation Nudging school, BE: promotion	Choices school: competitive foods removed and line re-purposed as NSLP cold lunch line Choices school: competitive food options removed Nudging school: fruit and milk placement in high traffic areas, whole fruit in colourful bowls, pre-sliced fruit in grab-n-go containers Nudging school: meal of the day promotional signage, posters of celebrities and athletes drinking milk
5	Bogart et al., 2011 [109]	Controlled before-after (non-random allocation of schools) Pilot 15 w (dates NR)	USA Los Angeles, California	2 middle schools, 1 intervention 1 I-school, 1 C-school Similar demographic data for ethnicity and 77% students eligible for FRP lunch I-school: *n* = 399 7th grade students completed pre and post surveys (50% female, mean age 13, SD 0.5); *n* = 140 7th grade student advocates; enrolled students or student sample size NR	To pilot a community-based intervention for adolescents, Students for Nutrition and eXercise (SNaX) to translate school obesity-prevention policies into practice through peer leader advocacy of healthy eating and school cafeteria changes	5 w (dates NR) BE: nutrition labelling BE: availability, accessibility Stakeholder engagement: students BE: promotion Student training	POS signage with nutritional information Introduction of pre-sliced fruit Formative research results from students Handouts to students; SNaX related cafeteria changes publicised during a 7th grade assembly; posters explaining how to read nutritional information Peer leader training
6	Bogart et al., 2014 [88]	Cluster randomised trial (controlled) 3.5 y (January 2009–June 2012)	USA Los Angeles, California	10 middle schools, 1 intervention Similar demographic data for ethnicity; >83% students eligible for FRP lunch; student age and gender NR 5 I-schools, *n* = 1515 mean number of students enrolled per school (SD = 323) 5 C-schools, *n* = 1524 mean number of students enrolled per school (SD = 266) *n* = 2997 7th grade students from I-school completed B and FU surveys	To conduct an RCT of SNaX, and examine effect on cafeteria participation, student eating behaviours and cafeteria attitudes	5 w per school (during spring semester each y; January to June) BE: nutrition labelling BE: availability, accessibility Stakeholder engagement: students BE: promotion Student training	POS signage with nutritional information ↑ variety of sliced/bite-sized fruit and veg, water stations with free chilled water, pre-sliced fruit and veg Lunchtime peer leader activities (wearing T-shirts, taste tests, distribution of promotional material) Posters marketing cafeteria changes; student taste testing Peer leader training to communicate SNaX messages
7	Bogart et al., 2018 [110]	Cluster non-randomised trial (controlled) 2 y (2013–2015)	USA Los Angeles, California	65 middle schools, 1 intervention *n* = 17 I-schools, *n* = 22311 enrolled students, 70% students in NSLP; *n* = 47 C-schools, *n* = 56,120 enrolled students, 86% students in NSLP *n* = 242 student advocates at end of I-year (student grade NR) *n* = 187 students completed student advocate surveys *n* = 154 student advocates participated in post-I focus groups	To disseminate an evidence-based middle-school obesity-prevention program, SNaX	5 w per school (1 y across all schools; 2014–2015) BE: availability, accessibility, acceptability Stakeholder engagement: students BE: promotion Student training	Introduction of pre-sliced fruit, water stations with free chilled water; student taste tests of food reformulated to be healthier Lunchtime activities including taste tests, distribution of promotional items (e.g., bookmarks), videos, lessons, kick-off assembly Cafeteria food focussed school-wide announcements and posters; student promotion of SNaX at lunchtime; student taste testing Student training to promote SNaX
8	Chu et al., 2011 [118]	Non-randomised trial (controlled, crossover) 1 y (spring and fall semesters 2009)	USA, Minnesota, Texas, urban and suburban areas	5 schools, 2 interventions 3 middle schools (1 Minnesota, 2 Texas), 2 high schools (1 Minnesota, 1 Texas) Demographics: Hispanic students, Texas range 25.7% to 54.5%, Minnesota range 1.4% to 35.6%; non-Hispanic, Texas range 1.7% to 47.3%, Minnesota range 26% to 94.7%; students eligible for FRP meals, range 30.5% to 100% across all schools; student age not reported	To compare student acceptance of whole-wheat vs. refined tortillas in school meals according to sensory attribute ratings	30 w (2 school semesters, 2009) Recipe changes Procurement	2 interventions to ↑ wholegrains and fibre intake. Replace refined tortillas in soft-taco entrée dish with (1) 66% white whole wheat tortilla, and (2) 100% white whole wheat tortilla Food supplier sourced for whole wheat tortillas
9	Cohen et al. 2012 [89] 2013 [119]	Cluster non-randomised trial (controlled, parallel arm) Pilot 2 y (2007–2009)	USA, MA, Boston	4 middle schools, 1 intervention 2 I-schools: 88% eligible for FRP meals, 78% participation in NSLP, *n* = 1609 student participants 2 C-schools, 86% eligible for FRP meals, 70% participation in NSLP, *n* = 1440 student participants Students in grades 6–8, most aged 12–14 years	2012: To evaluate the impact of chef-based model on student’s selection and consumption of school lunches 2013: To assess the impact of food waste on nutrient consumption, if school foods served could be valid proxies for food consumed, and costs associated with food waste	2 y (2007–2009) BE: acceptability Recipe changes Staff training	Meals modified to enhance palatability using sauces, seasoning, salad dressings Replace trans and saturated fats with unsaturated fats; ↓ added sugar and salt, ↑ wholegrains and fibre Professional chef engaged to train cafeteria staff to improve menu diet quality and cooking techniques
10	Cullen et al., 2007 [114]	Before-after Pilot 1 y (spring 2003–spring 2004)	USA, California, North Carolina, Texas	6 middle schools, 1 intervention 2 California, *n* = 2873 students 2 North Carolina, *n* = 1565 students 2 Texas, *n* = 1810 students Student age NR; baseline differences in ethnicity and eligibility for FRP meals (range, 55–97%) between schools	To examine the feasibility of instituting school food environment changes during a 6-week pilot in school foodservice programs	6 w (winter/spring 2004) BE: availability Stakeholder engagement: staff, students Staff training	Expand healthy menu options to meet goals: (1) serve ≥3 fruit and veg items/day, (2) include ≥10 different fruit and veg items/3-week period, (3) serve ≥2 lower-fat entrees/week Focus groups with students and school staff to inform the development of school foodservice changes Dietitian facilitated staff training to implement menu change goals
11	Cullen et al., 2008 [103] Mendoza et al., 2010 [104]	Before-after 5 y (2001–2006)	USA, Texas	3 middle schools, 1 intervention Students in grades 6–8; *n* = 2690 enrolled students across all schools (2001–2002 school year), and *n* = 3306 (2005–2006 school year) FRP eligibility, range 26–68% in 2001–2002, and 38–75% in 2005–2006	To assess the effect of the Texas Public School Nutrition Policy on middle school student lunchtime food consumption	2 y (2004–2006) Food standards implementation	Statewide policy that applied to all school food sources (NSLP, snack bars, vending): restrict portion size of high-fat and sugar snacks, SSBs and the fat content of all foods served; limit frequency of serving high-fat veg
12	Cullen et al., 2015 [90]	Cluster randomised trial (controlled, parallel arm) Pilot 15 w (fall 2011)	USA, TX, Houston	4 intermediate schools, 1 intervention 2 I-schools, 2 C-schools Student age or enrolment numbers NR; Sample size for observations, *n* = 427 students (I-schools, *n* = 212; C-schools, *n* = 215)	To investigate changes in student food selection and consumption in response to the new NSLP meal patterns during fall 2011	15 w (fall 2011) Food standards implementation BE: promotion	New regulations for allowable food serves for reimbursable meal: 1 fruit, 2 veg, 1 protein, 2 grain, 1 milk Colour displays of food at cafeteria entrance, signage with instructions for food selections, supporting materials for class teachers and parents
13	D’Adamo et al., 2021 [113]	Non-randomised trial (controlled, crossover) 2 y (dates NR)	USA, Maryland, Baltimore, urban area	1 high school, 1 intervention I-group (herbs and spices), C-group (typical recipe) *n* = 273 enrolled students Demographics: 57% female, African American 76% Hispanic 10%, ≥2 races 10%, White 4%, Asian < 1%, 100% eligible for FRP meals All students provided lunch trays for veg plate waste assessment	To determine whether stakeholder-informed addition of spices and herbs to NSLP veg would increase intake	4 school semesters (dates NR) Recipe changes, BE: acceptability Stakeholder engagement: staff, students BE: promotion	Addition of a variety of spices and herbs to 7 different NSLP veg recipes; student taste testing to inform recipe changes Engagement with school staff, teachers, food service staff and students to assess needs, attitudes and preferences for NSLP veg; health educators and professional chef led after-school student nutrition education and veg recipe sensory-testing; ‘Lunch Bunch’ student-led advocacy group ‘Lunch Bunch’ promoted veg recipes with spices and herbs (year 2 only); student created signage for display around the school; school announcements
14	Elbel et al., 2015 [107]	Cluster non-randomised trial (controlled) 11 m (November 2010–September 2011)	USA, New York, NYC	17 schools (includes elementary, middle and high schools; split between school type unknown), 1 intervention 8 I-schools, 9 C-schools I-schools: *n* = 1091 mean number of students/school, 55% female, 54% eligible for FRP meals, 21% African American, 41% Hispanic, 25% White, 11% Asian C-schools: *n* = 1175 mean number of students/school, 52% female, 47.1% eligible for FRP meals, 13% African American, 33% Hispanic, 33% White, 20% Asian Sub-set of larger study separated survey data for middle and high school (8th and 11th grade; *n* = 1759 students).	To determine the influence of water-jets on observed water and milk taking and self-reported fluid consumption in NYC public schools	10 m (December 2010–September 2011) Food service operations BE: accessibility, availability	Water jet installation near lunch line in cafeterias; water jets in place throughout post-I period of study; no other parallel interventions such as activities to promote water drinking Increased water access and availability
15	Ellison et al. 1989 a [115] 1989 b [100] 1990 [116]	Controlled before-after (non-randomised) 4 y (1984–1988)	USA, NH and MA	2 boarding high schools, 2 interventions (phase 1 and 2) Student mean age 15 years, almost none obese, 77% white 1989a: Sodium intake from food diary assessment, at B *n* = 674 (I-group *n* = 340, C-group *n* = 334), at FU *n* = 431 (I-group *n* = 221, C-group *n* = 210); 1700 ballots for food acceptability rating 1989b: BP assessment, *n* = 650 students (I-group *n* = 309, C-group *n* = 341) 1990: Fat intake from food diary assessment, at B *n* = 774 (I-group *n* = 389, C-group *n* = 385), at FU *n* = 467 (I-group *n* = 228, C-group *n* = 239)	To measure the effects of changes in food purchasing and preparation practices on student acceptability of modified foods, sodium and fat intake, and BP	6 m/phase (phase 1: reduced sodium; phase 2: modified fat; years unclear) Recipe changes, BE: acceptability Stakeholder engagement: staff Procurement Staff training	Phase 1 and 2: collaboration with nutritionist to revise menus and recipes: ↓ sodium in preparation, ingredient swaps to enhance flavour, modified fat in recipes (reduced saturated fat, increase polyunsaturated fat) Phase 1 and 2: meetings with production staff to develop modified recipes, food service staff taste testing of modified recipes Phase 1 and 2: procurement of alternate products with ↓ sodium, ↓ SFA and ↑ PUFA Phase 1 and 2: collaboration with nutritionist for staff training, 4 components: (1) healthy diet in early life, staff’s essential role, (2) recipe testing (3) serving-line staff training because of their direct interaction with students
16	Fritts et al., 2019 [120]	Phase 1: Non-randomised trial (controlled, crossover) Phase 2: Before-after 10 m (March–December 2017)	USA, Pennsylvania, rural area	1 middle/high school, 2 interventions (phase 1 and 2) I-group (herb and spice veg), C-group (lightly salted veg); approx. 75% students participate in the NSLP, and 44% received FRP lunch; 600–700 students aged 11–18 years were served lunch daily across 3 lunch periods School district demographics: 97% Caucasian	To test whether adding herbs and spices to school lunch veg increases selection and consumption compared with lightly salted veg among rural adolescents	10 m (March–December 2017) Recipe changes, BE: acceptability BE: presentation Stakeholder engagement: staff Staff training	Phase 1 (March–May 2017): addition of a variety of spices and herbs to 8 different NSLP veg recipes to enhance palatability compared to lightly salted versions (C-group); phase 2 (October–December 2017): repeated exposure of 2 modified veg recipes with herbs and spices Phase 2: black plastic containers used to present 2 veg dishes on offer and improve visual appeal Phase 1: staff taste testing of new recipes; Phase 2: collaboration between school foodservice director, cafeteria staff and researchers to select 2 most appropriate recipes to incorporate into regular menu Phase 1 and 2: Industry partner who developed recipes conducted a half-day training session with cafeteria staff to demonstrate recipe preparation
17	Greene et al., 2017 [91]	Cluster randomised trial (controlled) 9 w (February–April 2014)	USA, New York, urban and rural districts	7 middle schools, 1 intervention 4 I-schools (2 urban, 2 rural) and 3 C-schools (2 urban, 1 rural) I-schools: *n* = 1258 enrolled students, 1–97% white, 55–92% economic disadvantage C-schools: *n* = 850 enrolled students, 5–90% white, 49–92% economic disadvantage All students in grades 5–8, age NR	To evaluate the impact of fruit-promoting Smarter Lunchroom interventions on middle school students’ selection and consumption of fruit	6 w (March–April 2014) BE: accessibility, availability, presentation Stakeholder engagement: students Staff training BE: promotion	Smarter Lunchroom changes: fruit placed first on the line, at least two varieties offered in at least two locations, pre-sliced fruit in small attractive cups, whole fruit in large attractive bowls at eye level Student focus groups to generate creative names 30–60 min session for cafeteria staff and food service managers on how to make fruit-promoting changes Creative names for fruit labels and display on menus
18	Hackett et al., 1990 [121]	Controlled before-after (non-randomised) 1 y (July 1987–July 1988)	UK, Northum-berland county	4 middle schools, 2 interventions 2 ‘dish of day free-choice’ I-schools; 2 ‘2 course fixed price’ I-schools 2 ‘affluent’ and 2 ‘less well-off’ schools (each allocated 1 free-choice I-school and 1 fixed-price I-school); Approx. *n* = 830 students aged 11–12 years across all schools Completion of surveys with school meal participation data: survey 1, *n* = 674 (*n* = 301 from free-choice I-schools, *n* = 373 from fixed-price I-schools); survey 2, *n* = 692 students (*n* = 333 from free-choice I-schools, *n* = 359 from fixed-price I-schools)	To improve the quality of school meals and their up-take via a healthy eating campaign	10 m (October–December 1987) Recipe changes BE: availability (price targets) BE: promotion	Modified menus to improve nutritional quality; 2 interventions: (1) ‘dish of day free-choice’ I-schools, and (2) ‘2 course meal fixed price’ I-schools ‘2 course meal fixed price’ initiative Both interventions: campaign pack per student to take home with healthy eating guidelines, new menus, ‘champion eater’ report card
19	Hanks et al., 2012 [122]	Before-after 4 m (February–May 2011)	USA, New York, Corning	1 high school, 1 intervention	To examine the application of the principle that healthier foods are more likely to be consumed if they were more convenient than less convenient less healthy foods	2 m (April–May 2011) BE: accessibility Food service operations	More convenient access to healthier food options (sub-sandwich bar, salad bar, veg, whole fruit, fruit parfait) Conversion of 1 of 2 service lines to a ‘convenience line’ that only offered healthier food options (as above) and flavoured milk
20	Hanks et al., 2013 [97]	Before-after Pilot 4 m (March–June 2011)	USA, New York	2 high schools, 1 intervention Grades 7–12, student numbers, age and other demographics NR	To investigate how small changes to school cafeterias can influence the choice and consumption of healthy foods	2 m (May–June 2011) BE: presentation, accessibility Food service operations Stakeholder engagement: staff BE: promotion	Fruit displayed in bowls and tiered stands, salad served in see-through to-go containers, fresh fruit located next to cash register, 100% fruit juice boxes in freezer next to ice cream ‘Healthy convenience’ line with only sub-sandwiches and healthier sides Cafeteria staff engaged to implement verbal prompts Cafeteria staff verbal prompts to promote healthy choices, lunch menu posted with colour photos of fruit and veg served, veg labelled with descriptive names
21	Hunsberger et al., 2015 [123]	Before-after 4 m (January–April 2010)	USA, Oregon, rural area	1 middle school, 1 intervention Students in grades 6–8, aged 11–15 years, 64.6% of ethnic minority, 32.5% have BMI >95th percentile (obese), 79% eligible for FRP meals, *n* = 531 average number of students/day that participated in the NSLP (78%) during study period	To investigate the impact of POS calorie information	17 d (February 2010) BE: nutrition labelling	POS signage with calorie labels; consultation with Mountain View Community Health Improvement and Research Partnership for program development
22	Just et al., 2014 [93]	Before-after Pilot 3 m (February–April 2012)	USA, New York	1 high school, 1 intervention *n* = 370 enrolled students, aged 13–18 years; School district demographics: ethnicity primarily white (93.9%), eligibility for FRP meals 19.8%	To conduct a pilot test to gauge the feasibility of the Chef Moves To School program, and measure student response through lunch selection and consumption	2 d (April 2012) BE: acceptability Recipe changes Stakeholder engagement: students BE: promotion	Student taste testing of new pizzas Professional chef engaged to use ingredients available in school cafeteria to develop 3 types of pizza (meat taco, bean taco, garlic spinach) and a ranch flavoured burger; new chefs lunch items available in cafeteria on 1 d Engaging students during after-school event After-school event for students to taste the chef’s lunch on offer the following day, meet the chef, talk about her profession and new recipes created
23	Koch et al., 2020 [124]	Before-after 2 y (2017–2018)	USA, New York City, NY	7 high schools, 1 intervention All students eligible to participate; *n* = 5719 enrolled students across all schools, 74% eligible for FRP lunch, age NR	To measure the effects of major changes to school cafeterias (STARCafe) on school lunch consumption and factors that may influence consumption (i.e., seated time, attitudes towards school lunch, perception of cafeteria noise, school lunch participation)	1 y per school (2017–2018) BE: availability BE: presentation Food service operations Stakeholder engagement: staff BE: promotion	Increased frequency and prominence of deli sandwiches (turkey and cheese), entrée salads, popcorn chicken salad, chicken dumplings and veg fried rice with zucchini, and fast-food options (popcorn chicken and pizza; both served with fries); new menu items included tuna wrap, popcorn chicken wrap, and tuna salad Dining area changes included comfortable seating options, planters, other dividers, and garbage can enclosures that matched tables. Wall décor included addition of school name, mascot or theme, and school mission Service line changed to an open-choice line School principals worked with New York City Department of Education to create a table layout with variety of social arrangements. Posters and signage promoting education messages to inspire healthy choices, menu options, dining room directions, instructions
24	Madden et al., 2013 [105]	Before-after 3 w (2005)	UK, London	1 secondary school, 1 intervention Student participants aged 12–16 years, *n* = 378 lunch observations, pre-I *n* = 180 (38.9% female), post-I *n* = 198 (26.3% female) 63% students eligible for free school lunch	To examine the effect of a short, low-budget kitchen-based intervention on energy, nutrient, and fruit and veg intakes	1 w (2005) BE: availability (including price targets) Recipe changes Stakeholder engagement: staff Procurement Staff training	↑ fruit and veg offered with price targets to attract choice (salad bowls at no cost, variety of fresh fruit at cost), ↓ availability of less healthy options (larger packets of chips removed, only small chocolate bars) replaced with healthier alternative (reduced-fat cereal bars) Collaboration with dietitian to modify lunch ingredients: ↓ total fat and saturated fat, (salad added to baguettes, veg topped pizza, variety of fresh fruit, side salad) Collaboration with kitchen staff to develop menu changes Procurement of ↓ fat mayonnaise and cheese, trimmed bacon, new hot chip variety Dietitian facilitated 2hr education session with kitchen staff based around Eatwell Plate (UK guidelines)
25	McCool et al., 2005 [108]	Non-randomised trial (controlled, crossover) Pilot 12 w (dates NR)	USA, metropolitan area	1 middle school, 3 interventions (phase 1–3) Enrolled students, *n* = 1234, age NR, 87.4% eligible for FRP meals	To compare the amount of apple consumed by students when they were offered whole versus sliced ready-to-eat packaged apples	12 w (dates NR; phase 1 = 6 weeks, phase 2 = 4 weeks, phase 3 = 2 weeks) BE: accessibility, availability (price targets)	Apples offered to all students for free in addition to the regular lunch meal as (1) phase 1, whole apples, (2) phase 2, pre-sliced apples, and (3) phase 3, whole and pre-sliced apples; for all interventions fruit placed at the end of lunch line for students to take as many whole and/or pre-sliced apples as they wanted
26	Pope et al., 2018 [94]	Before-after Pilot 3 m (September–November 2015)	USA, Vermont, rural area	1 middle school, 1 intervention *n* = 587 eligible students in grades 4–8 eligible to participate; average NSLP participation rate = 66% Student age NR; numbers who participated in taste-testing NR	To investigate whether providing samples of a veg-focused lunch entrée the day before it appeared on the lunch menu ↑ NSLP participation	1 m (October 2015) Recipe changes BE: availability, acceptability Stakeholder engagement: students BE: promotion	4 new entrées developed by the research team, including 2 registered dietitians, and prepared by school food service staff: (1) chicken and broccoli alfredo, (2) root veg stew, (3) savoury turkey loaf, (4) eggplant parmesan. 1 new entrée offered/week Increased veg variety, students sample new entrée the day before offered in the cafeteria Students engaged for taste testing Students were invited to taste a sample of the new entrée the day before it was served
27	Prell et al., 2005 [101]	Controlled before-after (randomised) 5 w (1998–1999 school year)	Sweden, Göteborg	3 secondary schools: 2 interventions (1) C-group, no intervention, *n* = 83 students (63% participation) (2) SL-group (school lunch intervention), *n* = 58 students (51% participation) Grade 8, aged approx. 14 years (3) SLHE-group (SL + home economics intervention), *n* = 87 students (60% participation)	To examine the effectiveness of 2 school-based interventions aimed at increasing adolescents’ intake of fish at school	5 w Both groups, BE: acceptability, presentation Both groups: stakeholder engagement: students SLHE-group, student education Both groups: Staff training BE: promotion	Alternative fish dish served, improved accompaniments (choice of 2 sauces, freshly boiled potatoes), fish dish garnish, lunchroom decorated with fish-related objects Students voted for a fish dish for school lunch Modifications to curricula with 5 lessons about fish including a slide show of fish preparation in school kitchen 1 day staff training in fish preparation Fish dish on display
28	Prescott et al., 2019 [99]	Controlled before-after (non-random allocation of schools) 6 m (November 2017–April 2018)	USA, Colorado	2 middle schools, 1 intervention (1) I-group (poster + education), *n* = 268 grade 6 students across 2 schools (2) C-group (poster only), *n* = 426 students in grades 7–8 across 2 schools	To examine the impact of a student-driven sustainable food systems education and promotion intervention on adolescent school lunch selection, consumption and waste behaviours, particularly for fruit and veg, during school lunch	12–16 classes (from December 2017) + 2 weeks (April 2018) BE: presentation Stakeholder engagement: students Student education BE: promotion (2 weeks, April 2018)	Waste reduction posters displayed in cafeteria Grade 6 students consulted for development of promotional posters; grade 6–8 students voted for best posters for display in school cafeteria Teachers implemented a standards-based curriculum on sustainable food systems Posters promoting waste reduction displayed in school cafeteria
29	Quinn et al., 2018 [98]	Controlled before-after (non-random allocation of schools) 1 y (2013–2014 school year)	USA, Washington, King County	11 schools, 1 intervention 6 I-schools (3 middle and 3 high schools; *n* = 1026 mean number students enrolled per school), 5 C-schools (3 middle and 2 high schools; *n* = 1219 mean number students per school) *n* = 2309 tray observations across all schools and time points Student age not reported	To evaluate whether a year-long choice architecture intervention implemented by school cafeteria managers changed student selection and consumption of healthy foods	1 y (2013–2014) BE: presentation, availability, accessibility Stakeholder engagement: staff BE: promotion Staff training	Attractive containers, pre-sliced fruit, strategic placement Kitchen manager a member of the technical team (includes dietitian, school nutrition specialist and project lead) that provided implementation support Staff verbal prompts, creative naming and signage to promote healthy foods Training and support throughout school year to implement BE strategies based on Smarter Lunchroom principles
30	Schwartz et al., 2015 [92]	Before-after 3 y (2012–2014)	USA, Connecticut, New Haven, low-income urban area	12 middle schools, 1 intervention Approx. *n* = 680 enrolled students in grade 5 (all schools); Sample population followed over 3 years, *n* = 502 in grade 5 (2012), *n* = 465 in grade 6 (2013) and *n* = 373 in grade 7 (2014) School district demographics: >70% eligible for free-lunch, 13% for reduced-price; 47% African American, 38% Hispanic, 15% white	To examine food component selection and consumption data pre- and post- revisions to the NSLP nutrition standards and policies	2 y (2012–2014) Food standards implementation	Updated nutrition standards for the NSLP implemented in the 2012–2013 school year: ↑ wholegrains, new calorie limits by age group, ↓ sodium, different veg served each week, ↑ fruit and veg portion size
31	Sharma et al., 2018 [106]	Non-randomised trial (controlled, parallel arm) 4 w (November–December, y NR)	USA	1 middle-high school, 1 intervention I-group, 1 fast service lane (FSL) C-group, 2 regular service lanes (RSL) Approx. *n* = 650 enrolled students in grades 6–12	To investigate whether middle and high school students are averse to loss of time and to assess feasibility of a fast food service lane intervention that would serve limited choices of pre-plated lunch meals	4 w (November–December, year NR) Food service operations (includes BE: accessibility, availability) BE: promotion	Re-configure 1 of 3 lunch service lanes into a pre-plated FSL that offered pre-plated fruit, veg sides; students allowed 1 of 2 entrees on offer that day Promotional posters to alert students to the new FSL were strategically placed in the school a week prior to the start of the field experiment
32	Turnin et al., 2016 [112]	Before-after 1 y (dates NR)	France, Toulouse, suburban and urban areas	3 middle schools (1 suburban, 2 urban), 1 intervention *n* = 350 students for analysis, mean age 13.3 years (range, 11.5 to 16.4 years) School A, B and C; *n* = 84, 88 and 178 students respectively	To evaluate the impact of interactive Nutri-Advice kiosks on children’s nutritional skills and their ability to apply it to food choices in a middle school cafeteria menu (food choice competencies)	6 m (November–May, year NR) Food service operations	Installation of kiosk stations with Nutri-Advice software for children to assess and select a well-balanced meal from daily food available on cafeteria menu
33	Wansink et al., 2015 [95]	Before-after Pilot 2 m (March–April 2012)	USA, New York, Lansing	1 high school, 1 intervention *n* = 370 enrolled students in grades 9–12; age not reported School district demographics: 93.9% white, 2% African American; 19% students eligible for FRP lunch	To examine the potential impact that a school garden intervention, independent of corresponding educational materials, has on students veg selection and intake	1 d (24 April 2012) Recipe changes BE: presentation, acceptability Procurement BE: promotion	School garden leafy greens were harvested and included for service at the salad bar Salad garnish (raspberries) and raspberry vinaigrette dressing Sourcing school garden produce School announcements, colourful signage advertising salads served that day included school garden leafy greens
34	Wansink et al., 2013 [111]	Cluster randomised trial (controlled) Duration unclear (2011)	USA, New York, Wayne County	6 middle schools, 1 intervention 3 I-schools, 3 C-schools *n* = 2150 enrolled students across all schools	To determine the effect of offering pre-sliced fruit in schools on selection and intake	1 m (November 2011) BE: accessibility	Cafeteria staff provided pre-sliced apple upon student apple request
35	Witschi et al. 1985 [125]	Before-after Pilot 9 w (Oct-Nov 1982)	USA, New Hampshire	1 boarding high school, 1 intervention Approx. *n* = 1000 enrolled students; To monitor sodium intake: *n* = 228 students aged 15–18 years Palatability survey responses: *n* = 1036 (pre-I) and 748 (during-I)	To test the effects of dietary modification on total sodium intake of students and assess palatability for adolescents	5 w (October–November 1982) Recipe changes Procurement Stakeholder engagement: staff	Recipes modified to ↓ sodium, replace with non-sodium containing spices; frequently consumed commercially produced items (for example, meat products, cheese, potato chips) were replaced with ↓ sodium alternatives; foods obviously high in sodium omitted; students advised not to modify other aspects of lifestyle during study period, and encouraged not to add salt to food at the table Procurement of alternate products with ↓ sodium Modified recipes sampled by food service staff to assess palatability

* NOURISHING frameworks’ domains, denoted by shade colour: blue = food environment domain; green = food system domain; orange = behaviour change communication domain. ^+^ Duration: y: year/s; m: month/s; w: week/s; d: day/s. B: baseline; BE: behavioural economics; BP: blood pressure; C: control or comparison; CBPR: community-based participatory research; FU: follow-up; I: intervention; FRP: free or reduced-price; NR: not reported; NSLP: National School Lunch Program; POS: point of selection; SD: standard deviation; SSB: sugar sweetened beverages; Veg: vegetables; #: number; RCT: randomized controlled trial; SFA: saturated fatty acids; PUFA: polyunsaturated fatty acids; BMI: body mass index; ↑: increase; ↓: decrease; NYC: New York city.

**Table 3:** NOURISHING framework’s domains and action areas, and their application in the current review.

Domain *	Action Areas *		Sub-Action Areas Relevant to the Current Review *	Classification of Intervention Strategies from Included Studies
**Food environment**	**N**	Nutrition label standards and regulations on use of claims and implied claims on food	Interpretive labelling On-shelf labelling Calorie and nutrient labelling on menus and displays	Promotion—nutrition labelling on menus or at point of selection (e.g., traffic light, calorie or nutrient labelling)
	**O**	Offer healthy food and set standards in public institutions and other specific settings	Fruit and vegetable initiatives in schools Mandatory standards for food available in schools including restrictions on unhealthy food Voluntary guidelines for food available in schools Choice architecture	Food standards or policy implementation Implementation of voluntary policy or guideline initiatives Implementation of updated policy or national guidelines Presentation—improvements to the physical environment (e.g., dining room layout including wall decor, table arrangement) or presentation of food (e.g., attractive containers for healthy food, garnish on meals) Accessibility—placement, convenience (includes pre-sliced fruit/veg)
	**U**	Use economic tools to address food affordability and purchase incentives	Targeted subsidies for healthy food	Price targets to attract choice towards healthier options
	**R**	Restrict food advertising and other forms of commercial promotion	NA for this review	NA
	**I**	Improve nutritional quality of the whole food supply	Voluntary reformulation of food products Voluntary commitments to reduce portion sizes Limits on availability of high-fat meat products and high-sugar food products and beverages	Reformulation of recipes or menu to enhance nutritional quality; includes engagement of professional chef or dietitian Acceptability—taste-testing to inform changes; seasoning or sauces to enhance palatability Limits on fat and sugar in meals, and restrict portion sizes
	**S**	Set incentives and rules to create a healthy retail and food service environment	Initiatives to increase the availability of healthier food in stores and out-of-home venues Incentives and rules to offer healthy food options as a default in food service outlets Incentives and rules to reduce salt in food service outlets	Changes to food service operations such as modifications to the point of service or service lines Availability—increased variety or expand healthy options (includes offering pre-sliced fruit in addition to whole fruit); reduced availability of less healthy food and beverages
**Food system**	**H**	Harness food supply chain and actions across sectors to ensure coherence with health	Working with food suppliers to provide healthier ingredients Public procurement through ‘short-chains’ (e.g., local farmers) Community food production Governance structures; multi-sector/stakeholder engagement	Stakeholder engagement—student, school staff or food service staff engaged to participate in program development or implementation Sourcing healthier ingredients from food suppliers Using school garden produce in meal preparation
**Behaviour change communication**	**I**	Inform people about food and nutrition through public awareness	Public awareness, mass media and informational campaigns and social marketing on healthy eating, fruit and vegetables, unhealthy food and beverages, or concerning salt	Promotion and marketing—student sampling, posters, table tents, food naming, school announcements or food service staff prompts
	**N**	Nutrition advice and counselling in health care settings	NA for this review	NA
	**G**	Give nutrition education and skills	Nutrition education on curricula Community-based nutrition education Cooking skills Initiatives to train school children on growing food Training for caterers and food service providers	Food service staff training School garden initiatives with students and food service staff Student education or skills training related to modifications to the meal service

* This material has been reproduced from the World Cancer Research Fund International NOURISHING framework https://www.wcrf.org/int/policy/policy-databases/nourishing-framework (accessed on 26 July 2019). [75]; Hawkes et al., 2013 [76]; NOURISHING frameworks’ domains, denoted by shade colour: blue = food environment domain; green = food system domain; orange = behaviour change communication domain. NA: not applicable.

**Table 4:** Intervention strategies categorized according to the NOURISHING frameworks’ domains and relevant action areas *.

		Food Environment					Food System	Behavior Change Communication	
		N	O	U	I	S	H	I	G
Author	Number of Action Areas Targeted	Nutrition Label Standards and Regulations on Use of Claims and Implied Claims on Food	Offer Healthy Food and Set Standards in Public Institutions or Other Settings	Use Economic Tools to Address Food Affordability and Purchase Incentives	Improve Nutritional Quality of the Whole Food Supply	Set Incentives and Rules to Create a Healthy Retail and Food Service Environment	Harness Food Supply Chain and Actions Across Sectors to Ensure Coherence with Health	Inform People about Food and Nutrition through Public Awareness	Give Nutrition Education and Skills
**Interventions that include strategies across three domains:**									
Bhatia et al., 2011 [44]	6		•		•	•	•	•	•
Bogart et al., 2011 [109]	6	•	•			•	•	•	•
Bogart et al., 2014 [88]	6	•	•			•	•	•	•
Bogart et al., 2018 [110]	6		•		•	•	•	•	•
Askelson et al., 2019 [117]	5		•			•	•	•	•
Greene et al., 2017 [91]	5		•			•	•	•	•
Madden et al., 2013 [105]	5			•	•	•	•		•
Prell et al., 2005 1. SL [101]	5		•		•		•	•	•
Prell et al., 2005 2. SLHE [101]	5		•		•		•	•	•
Quinn et al., 2018 [98]	5		•			•	•	•	•
Fritts et al., 2019 1. Phase 1 [120]	4		•		•		•		•
Fritts et al., 2019 2. Phase 2 [120]	4		•		•		•		•
Hanks et al., 2013 [97]	4		•			•	•	•	
Koch et al., 2020 [124]	4		•			•	•	•	
Pope et al., 2018 [94]	4				•	•	•	•	
Prescott et al., 2019 [99]	4		•				•	•	•
Wansink et al., 2015 [95]	4		•		•		•	•	
Cullen et al., 2007 [114]	3					•	•		•
D’Adamo et al., 2021 [113]	3				•		•	•	
Ellison et al., 1989a [115], 1989b [100]	3				•		•		•
Ellison et al., 1990 [116]	3				•		•		•
Just et al., 2014 [93]	3				•		•	•	
**Interventions that include strategies across two domains:**									
Bean et al., 2019 [102]	4		•			•		•	•
Boehm et al., 2020 2. Nudges [96]	3		•			•		•	
Hackett et al., 1990 2. Fixed price [121]	3			•	•			•	
Sharma et al., 2018 [106]	3		•			•		•	
Chu et al., 2011 1. 66% wholewheat [118]	2				•		•		
Chu et al., 2011 2. 100% wholewheat [118]	2				•		•		
Cohen et al., 2012 [89], 2013 [119]	2				•				•
Cullen et al., 2015 [90]	2		•					•	
Hackett et al., 1990 1. Dish of day [121]	2				•			•	
Witschi et al., 1982 [125]	2				•		•		
**Interventions that include a strategy or strategies in one domain only:**									
McCool et al., 2005 1. Phase 2 vs. 1 [108]	3		•	•		•			
McCool et al., 2005 2. Phase 3 [108]	3		•	•		•			
Elbel et al., 2015 [107]	2		•			•			
Hanks et al., 2012 [122]	2		•			•			
Boehm et al., 2020 1. Choices [96]	1					•			
Cullen et al., 2008 [103]; Mendoza et al., 2010 [104]	1		•						
Hunsberger et al., 2015 [123]	1	•							
Schwartz et al., 2015 [92]	1		•						
Turnin et al., 2016 [112]	1					•			
Wansink et al., 2013 [111]	1		•						
		3	26	4	19	21	25	22	18

* table excludes NOURISHING frameworks’ action areas not relevant for this review; * NOURISHING frameworks’ domains, denoted by shade colour: blue = food environment domain; green = food system domain; •: indicates the intervention has included the NOURISHING framework action area according to our classification.

**Table 5:** Direction of effect sensitivity analysis for each outcome domain by study quality, study design, intervention duration, NOURISHING domains, NOURISHING action areas, stakeholder engagement, and behaviour change communication.

Outcome Domain		Interventions, *n*	Positive Impact, *n* (%)	Negative Impact, *n*	No Change or Mixed Effects	Sign Test, *p*-Value *	95% CI **
**Selection of a meal component**							
Study quality	Positive rating	8	5 (63%)	0	3	0.063	31% to 86%
	Neutral rating	17	10 (59%)	2	5	0.039	36% to 78%
Study design	Pre-post assessment	22	15 (68%)	2	5	0.002	47% to 84%
	Parallel arm or crossover	3	0 (0%)	0	3	NA	NA
Intervention duration	≤2 months	15	12 (80%)	1	2	0.003	55% to 93%
	3+ months	10	3 (30%)	1	6	0.625	11% to 60%
NOURISHING domains	Three domains	15	10 (67%)	1	4	0.012	42% to 85%
	One or two domains	10	5 (50%)	1	4	0.219	24% to 76%
NOURISHING action areas	Three to six action areas	16	11 (69%)	1	4	0.006	44% to 86%
	One to two action areas	9	4 (44%)	1	4	0.375	19% to 73%
Stakeholder engagement	Student engagement	9	7 (78%)	0	2	0.016	45% to 94%
	Without	16	8 (50%)	2	6	0.109	28% to 72%
Behaviour change communication	Promotion and/or training	18	11 (61%)	1	6	0.006	39% to 80%
	Without	7	4 (57%)	1	2	0.375	25% to 84%
**Consumption of a meal component**							
Study quality	Positive rating	3	0 (0%)	0	3	NA	NA
	Neutral rating	21	14 (67%)	3	4	0.013	45% to 83%
Study design	Pre-post assessment	18	11 (61%)	2	5	0.022	39% to 80%
	Parallel arm or crossover	6	3 (50%)	1	2	0.625	19% to 81%
Intervention duration	≤2 months	13	8 (62%)	2	3	0.109	36% to 82%
	3+ months	11	6 (55%)	1	4	0.125	28% to 79%
NOURISHING domains	Three domains	15	9 (60%)	2	4	0.065	36% to 80%
	One or two domains	9	5 (56%)	1	3	0.219	27% to 81%
NOURISHING action areas	Three to six action areas	17	11 (65%)	2	4	0.022	41% to 83%
	One to two action areas	7	3 (43%)	1	3	0.625	16% to 75%
Stakeholder engagement	Student engagement	6	5 (83%)	0	1	0.063	44% to 97%
	Without	18	9 (50%)	3	6	0.146	29% to 71%
Behaviour change communication	Promotion and/or training	17	9 (53%)	2	6	0.065	31% to 74%
	Without	7	5 (71%)	1	1	0.219	36% to 92%
**Meal program participation rate**							
Study quality	Positive rating	0	0 (0%)	0	0	NA	NA
	Neutral rating	5	3 (60%)	2	0	NA	23% to 88%
Study design	Pre-post assessment	5	3 (60%)	2	0	NA	23% to 88%
	Parallel arm or crossover	0	0 (0%)	0	0	NA	NA
Intervention duration	≤2 months	1	1 (100%)	0	0	NA	NA
	3+ months	4	2 (50%)	2	0	NA	15% to 85%
NOURISHING domains	Three domains	3	3 (100%)	0	0	0.250	44% to 100%
	One or two domains	2	0 (0%)	2	0	NA	0% to 66%
NOURISHING action areas	Three to six action areas	4	3 (75%)	1	0	0.625	30% to 95%
	One to two action areas	1	0 (0%)	1	0	NA	NA
Stakeholder engagement	Student engagement	2	2 (100%)	0	0	0.500	34% to 100%
	Without	3	1 (33%)	2	0	NA	6% to 79%
Behaviour change communication	Promotion and/or training	5	3 (60%)	2	0	NA	23% to 88%
	Without	0	0 (0%)	0	0	NA	NA
**Attitudes and perceptions related to changes to the meal service**							
Study quality	Positive rating	3	3 (100%)	0	0	0.250	44% to 100%
	Neutral rating	10	6 (60%)	4	0	0.754	31% to 83%
Study design	Pre-post assessment	9	8 (89%)	1	0	0.039	57% to 98%
	Parallel arm or crossover	4	1 (25%)	3	0	0.625	5% to 70%
Intervention duration	≤2 months	4	4 (100%)	0	0	0.125	51% to 100%
	3+ months	9	5 (56%)	4	0	NA	27% to 81%
NOURISHING domains	Three domains	8	6 (75%)	2	0	0.289	41% to 93%
	One or two domains	5	3 (60%)	2	0	NA	23% to 88%
NOURISHING action areas	Three to six action areas	9	7 (78%)	2	0	0.180	45% to 94%
	One to two action areas	4	2 (50%)	2	0	NA	15% to 85%
Stakeholder engagement	Student engagement	3	3 (100%)	0	0	0.250	44% to 100%
	Without	10	6 (60%)	4	0	0.754	31% to 83%
Behaviour change communication	Promotion and/or training	9	7 (78%)	2	0	0.180	45% to 94%
	Without	4	2 (50%)	2	0	NA	15% to 85%

**LEGEND:** Study quality: variables apportioned per risk of bias assessment results as either, (1) positive rating, or (2) neutral rating; Study design: variables apportioned according to measurement scores used for analysis as either, (1) pre-post measurements = intervention arm before and after scores, or (2) parallel arm or crossover = comparison of post-intervention scores; Intervention duration: variables apportioned according to duration of intervention implementation as either, (1) ≤2 months, or (2) 3+ months; NOURISHING domains: variables apportioned according to number of NOURISHING framework domains as either, (1) interventions targeting 3 domains, or (2) interventions targeting 1–2 domains. NOURISHING action areas: variables apportioned according to number of NOURISHING framework action areas as either, (1) interventions targeting 3–6 action areas, or (2) interventions targeting 1–2 action areas; Stakeholder engagement: variables apportioned according to presence of stakeholder engagement during intervention development and/or implementation as either, (1) with students, or (2) without students; Behaviour change communication: variables apportioned for interventions as either, (1) including promotion and/or training, or (2) without promotion and/or training. **TABLE NOTES:** * Sign test excludes studies with no change/mixed effects direction as they cannot be said to represent either a positive or a negative effect direction [86]; NA denotes unstable point estimate due to low number of studies. ** 95% Confidence interval (CI) estimation for binomial proportions using the Wilson interval method [85,87].

## 4. Discussion

Our systematic review included 42 interventions across 35 studies that focused on modifying the school’s routine meal service. Results from our meta-analysis indicate significant improvements in student’s fruit and vegetable selection and consumption. The vote-counting synthesis found more than half of the interventions had a positive impact on selection and consumption of a meal component, program participation rate, and attitudes and perceptions related to changes. There were only a few studies that assessed health outcomes and service speed, all of which showed promising benefit. These results support existing evidence that nutrition interventions targeting the school food environment can improve students’ food behaviours, health and dining experience [39,41,49,58].

### 4.1. Interpretation of Results

Utilising the NOURISHING framework to unpack each intervention strategy allowed an examination of their component parts across environmental and behavioural contexts. In other words, each strategy was not limited to one domain and one action area. For example, Hanks et al. [97] introduced and promoted a healthy convenience line, improved presentation and accessibility of fruit and vegetables, and implemented staff prompts (3 domains, 4 action areas). This examination was useful to identify trends in intervention impact according to the component parts, as previous literature has recognised the challenge of identifying the ‘active’ ingredient within multi-strategy interventions [58,126,127]. In particular, we found interventions that showed most impact incorporated student and/or staff engagement, targeted more NOURISHING framework domains or action areas, increased accessibility of healthier options, or reduced the availability of less healthy options. This highlights opportunities to scale-up future nutrition interventions in this setting by utilising the NOURISHING framework in program design and development. Importantly, the interventions showing less impact were those that excluded collaboration with key stakeholders; primarily the staff who prepare the food, and students who consume the food. Overall, the range of intervention strategies for examination was diverse which is similar to other reviews examining the school food environment [39,49,58,60,63]. There were novel interventions (for example, interactive kiosks to encourage healthy lunch choices [112]), simple interventions (for example, installation of water jets near the lunch line to increase water consumption [107]), and multi-strategy interventions (for example, integrating behavioural economics, staff training and promotional activities).

Selection and consumption of a meal component (together or separately) were the predominate outcomes measured in our review, which is consistent with recent reviews [39,46,47,48,49]. While selection does not accurately reflect or guarantee consumption, food cannot be consumed until it’s selected. Therefore, both measures can provide important insight for decision making around food choices or dietary intake and food waste. For example, two high quality and controlled studies warrant comparison. Firstly, Cohen et al. [89,119] engaged a professional chef to improve menu quality and modify cooking techniques to enhance palatability. Increasing selection was effective for only one of six meal components (wholegrains), but students consumed a greater proportion of the vegetables they selected. While change in selection was limited, the results highlight good news for palatability and diet quality (increased fibre, vitamin A, and vitamin C, and reduced saturated fat), and reduced food waste. Secondly, Cullen et al. [90] investigated changes in student food selection and consumption in response to the updated United States Department of Agriculture (USDA) School Meal Standards that allowed increased servings of fruit and vegetables. Students selected more fruit and vegetables; however, they did not consume a greater proportion what they selected, resulting in more food waste. This comparison reinforces the importance of addressing palatability. The best interventions will increase consumption without increased waste through additional selection that is only to be discarded.

In other studies (not included in this review), Cohen and colleagues advocate for engagement with professional chefs following their examination of a chef-initiative to enhance palatability of school meals, on student consumption in elementary and middle schools [128,129]. The studies included both short-and longer-term exposure to chef-enhanced meals, and choice architecture components were incorporated in combination and separately to examine their impact. Overall results (not separated by school type) showed that longer-term exposure to the chef initiative increased consumption of both fruit and vegetables. Interestingly, there was no effect on consumption during the short-term exposure to the chef initiative or the ‘choice architecture only’ intervention. The missing link that may further entice students to select and consume reformulated meals in the short-and longer-term is student sampling of new recipes. Other reviews highlight, and advocate for, the potential synergistic impact of engaging professional chefs and dietitians, improving staff skills and active involvement with students through food preparation and sampling to address palatability and improve student dietary intake [49,59]. 

Measurements of attitudes and perceptions contributed useful insight into students dining experience. For example, Fritts et al. [120] found with repeat exposure, students were willing to eat vegetables again that had added herbs and spices, Sharma et al. [106] found students were satisfied with service speed and the quality of healthier meals made available from a fast service lane, and Koch et al. [124] found students had a positive attitude toward changes to the dining room. Furthermore, several authors commented on aspects of intervention success in their discussion. While clear evidence was not provided, these comments add insight into stakeholder and end-user experience within the school dining room of pragmatic trials. For example, Chu et al. [118] commented that food service staff were not provided with instructions for preparing alternative lunch products, and Just et al. [93] highlighted the taste-testing event was an integral part of the overall experience for students. Previous studies have recognised a multitude of other factors that influence the success (or not) of school-based interventions such as acceptability, implementation fidelity, organisational and staff readiness to participate, adequate training and collaboration between stakeholders [130,131]. Additionally, this can contribute to future directions. The school mealtime environment and dining experience is important to foster positive attitudes and values, build skills, promote health and socialise with peers [132]. Therefore, measurements of stakeholder attitudes and perceptions need to be considered in this setting. In particular, to further explore adolescents’ views on what can and should be done to improve nutrition and the food culture within a school dining room. 

The findings from the meta-analysis did not indicate any clear pattern of effect according to study quality, study design or intervention duration. Eligible studies for the meta-analysis were limited and varied in intervention duration and types of strategies implemented. The significant improvements across meta-analyses were small on each occasion. We suggest all of these factors contributed to the high heterogeneity, and therefore limit the confidence in pooled estimates. Further examination using the NOURISHING framework in conjunction with sensitivity analyses of intervention effect direction, identified trends in study methodology or intervention strategies. Shorter interventions were more beneficial than longer interventions which may suggest a novelty effect. This concurs with other reviews of school-based interventions that found longer interventions are not necessarily more effective [58,63]. Our results support recommendations to explore strategies that maintain momentum to build sustained change [63]. Again, we suggest evaluation of stakeholder satisfaction and implementation fidelity in the school dining room where most students have daily exposure to the intervention. This could provide useful insight into sustained engagement, proper processes and a feedback loop (potentially to modify design and re-implementation) to maintain momentum over longer-term periods. Another notable trend showing more benefit included interventions incorporating student engagement. Of note, three studies evaluated a community-based participatory research program in middle schools via a pilot, efficacy and dissemination test [88,109,110]. On each occasion, peer advocacy was a key component, initially adopted in the pilot study, and guided by the ‘diffusion of innovation’ theory that suggests ≥15% of the target population be trained as advocates [109]. These findings contribute to evidence that advocate for stakeholder engagement, in particular the adolescents themselves and food service staff, throughout the intervention process for enhanced success [58,133,134,135]. The clinical significance associated with ‘active involvement’ and collaboration between stakeholders was recognised in several included studies [93,102,117,125].

We note interventions that targeted more NOURISHING framework domains or action areas were more beneficial than those targeting fewer which is consistent with the frameworks’ recommendations for a comprehensive response to effectively promote healthy eating [76]. However, we cannot ignore the success of some interventions that targeted fewer. For example, implementing updated school nutrition standards is not a simple strategy, and such mandated change can improve targeted food behaviours in the school dining room as evidenced in three included studies [90,92,103]. While these studies varied in levels of success and outcomes measured, barriers and enablers were not evident. Previous reviews have highlighted enablers to successful school food policy implementation include adequate support from schools, positive staff attitudes, training, collaboration, communication, tailoring to local contexts and adequate planning [136,137]. This reinforces the concept that multiple factors can impact intervention success in the school-based setting. We suggest incorporating qualitative work that could provide important insight to better understand the barriers and enablers to intervention success. 

Other notable findings include the benefits evident from increasing accessibility of healthier options. This review exposed a variety of strategies, illustrating how accessibility of healthier options can take many forms and are important in a school setting where the lunch break time is limited. The placement and convenience of healthier options can encourage selection and reduce time in the lunch line. This allows more time to sit and eat lunch, which has been shown to improve consumption of fruit, vegetables, entrees and milk, and therefore reduced waste [138,139]. Qualitative studies exploring adolescent perceptions of school food indicate lengthy queues are a barrier to a positive dining experience, access to fresh food a facilitator, and availability of less healthy options detract from healthier choices [140,141,142]. Most studies in the current review were conducted in the US where competitive foods (often less healthy options from alternative menu offerings, school stores, snack bars, or vending machines that compete with school meal program participation) have a significant presence in schools. In our review removing competitive foods or reducing less healthy options had beneficial results [44,103]. This supports other evidence that such strategies can improve student participation in meal programs [143,144], and selection and consumption of healthier food [145], therefore improving diet quality. Interestingly, adolescents have identified what they were being taught at school conflicts with the availability and accessibility of unhealthy food offered at school, sending mixed messages to students and giving rise to unsupportive food environments [141,146]. This highlights the importance of including adolescents as key stakeholders who can offer valuable insights into where the individual issues lie, and they should be engaged in co-designing the solutions. 

Promotion strategies included events for student sampling, awareness campaigns, nutrition labelling, dining room signage, peer advocates, staff verbal prompts, school announcements. While it is difficult to understand exactly how effective these strategies are without direct feedback from staff or students, they contribute visual and strong marketing appeal, and potential synergistic impact alongside other strategies, to steer decision making or thoughts about food and eating. An Australian study of 12,188 secondary students aged 12–17 found that cumulative exposure to food marketing was positively linked to adolescents’ food choices and eating behaviours [147], and another study reframed food marketing to reject junk food in favour of healthier alternatives, and found positive and sustained change in dietary attitudes and food choices [148]. Knowing the social ecology of adolescents lives has changed rapidly with the emergence and globalisation of all forms of media and communication [149], which now forms part of their daily lives, this may be a promising area for closer examination within a schools dining room.

### 4.2. Limitations

Most studies in this review were repeat cross-sectional by design, a large proportion were before-after studies or non-randomised trials, and many were pilot studies. These study designs are not methodologically strong, and generally at higher risk of bias, placing limitations on the representativeness of results and conclusions about why and how changes occurred. However, they do provide an estimate of the likely impact of interventions, and in our review, the benefit (or not) of their component parts using the NOURISHING framework. Future work can build the evidence with larger-scale pragmatic trials under real-world conditions for measures of effectiveness driven by a sample that includes more schools along with the use of validated measurement tools and adequate statistical analyses. Low- and lower-middle income countries were excluded from our review to increase generalisability among schools in countries with similar nutrition governance. Feeding programs in these regions typically focus on micro-nutrient deficiencies and undernutrition. However, the coverage of school feeding programs in low- and lower-middle income countries is increasing and should be considered for future reviews. Interventions were required to focus on modifications to the meal service at school, and eligible outcomes for the selection or consumption of a meal component were restricted to measurements within this setting rather than habitual intake. This means some studies in secondary schools that modified food service practices alongside physical activity strategies and/or classroom nutrition education and measured dietary intake from total diet were excluded.

The short duration of some interventions limits reliability and strength of results. Without longer-term implementation and follow-up, the results may indicate a novelty rather than any sustained effect. For example, Wansink et al. [111] implemented an effective single-strategy sliced-apple intervention over one month in intervention schools with two days data collection (one week apart) to assess student selection and consumption. While as a stand-alone study these results have limitations, the findings contribute to the formative research (interviews with elementary and middle school students) and pilot study indicating student preference for pre-sliced fruit. Furthermore, a recent review found the majority of studies offering pre-sliced fruit (6 of 8) had a positive association with fruit consumption [39]. Another example included in this review by the same group of researchers examined whether promoting and incorporating school garden produce into school salads impacted student selection and consumption. This pilot study was conducted over one day only and resulted in an increase in salad selection but not consumption [95]. 

Most studies were not eligible for meta-analysis due to missing data such as sample size, inadequate statistical analysis (e.g., statistical significance often not assessed for outcome measures, meal program participation rates or percentage of students selecting food items) or a unique outcome measure or unit of measurement not common across other studies. High levels of heterogeneity were evident. Contributing factors include the range of study designs, type and duration of interventions. There were limitations associated with data synthesis using vote-counting based on direction-of-effect which is less powerful than methods combining statistical significance. The binary measure of effect as either ‘positive’ or ‘not’ does not account for varying sample sizes or provide information on the magnitude of effect. However, this synthesis method was appropriate for this review due to the inconsistency of available data across all studies [85].

Some studies that included primary and secondary schools disaggregated some, but not all outcome data by school type or age group. Therefore, some study’s results were excluded from this review [102,107,118]. This limitation has been identified in other reviews that highlight the importance of disaggregating data to recognise differences in food behaviours according to life stage [49], in this case the differences between childhood and adolescence characterised by unique biological change, increased autonomy, peer influence and social development that can influence their decisions around food and eating [149].

Most studies were conducted in the US, similar to other reviews that have assessed nutrition-related interventions at school [46,47,48,49,62]. There is a need for studies across a wider variety of settings including boarding schools and school meals outside the US that target the adolescent population. Well-designed and higher quality pragmatic trials are required. There is an opportunity for enhanced impact through engagement with end users (including the students and staff) in the design of new and novel interventions. For example, one of the most successful trials in this review used an interactive kiosk and a points system similar to a computer game to select a balanced lunch meal [112]. Further trials that take a novel approach and integrate technology and end user involvement in the school dining room are needed, rather than implementing the same old strategies with moderate to no effectiveness for improved nutrition. There is a need to further evaluate changes implemented within a meal service such as assessment of implementation fidelity, stakeholder feedback via survey and/or qualitative methods. A mixed-methods approach incorporating qualitative research methods can provide an insight into stakeholders’ perception of an intervention that strengthens our understanding of adolescents’ food behaviours and their dining experience; why they eat the way they do, their attitudes toward food and eating, and what influences food selection and consumption [150].

### 4.3. Implications 

Adolescents who participate in a school meal program or attend a boarding school consume a significant proportion of their daily dietary intake while at school. Therefore, access to nutritious, palatable and appealing meals is paramount and should be mandated through policy. Targeting school menus by increasing wholefoods and decreasing the availability and affordability of less healthy options is actionable. Importantly, this translates to an increase in diet quality (macro-and micronutrients) as evidenced in this review among studies that measured nutrient intake [89,103,104,105,115,116,119,125]. The ultimate objective is to reverse the trend of poor diet quality during adolescence and influence their habitual food behaviours as they transition to adulthood, to reduce their risk and burden of disease. Our review has exposed the following opportunities, across environmental and behavioural contexts, for interventions within a schools’ routine meal service to improve adolescent food behaviours, health and dining experience:

- Engage the stakeholders who prepare the food (food service staff) and consume the food (adolescent students) through formative research, program development and/or implementation; recruit peer advocates to act as change agents;
- Explore novel approaches in the school dining room such as integrating technology which now forms part of adolescents daily lives;
- Ensure nutritional quality of school menus alongside assessment of palatability; they must go hand in hand to increase consumption, reduce waste, and improve students’ diet quality. Allow students to sample modified foods, and if feasible, engage experts in the field of food and nutrition (dietitians, school nutrition specialists or professional chefs) to inform recipe or menu reformulation;
- Healthy options must be accessible (front and centre), visually appealing (showcase them), and fast to access because time allowed for lunch at school is limited;
- Restrict the availability and portion size of less healthy options. Students can only make decisions based on the options placed in front of them;
- Include marketing strategies and positive health messaging to engage adolescents and promote positive changes to the meal service;
- Use short-and longer-term evaluations to monitor progress and build sustained change;
- Measure selection and consumption of meal components to assess intake and waste.

## 5. Conclusions

The impact of a routine school meal service on adolescents’ food behaviours, health and dining experience is affected by multiple factors including the food environment, food service practices and skills, nutritional quality and palatability of menus, schools’ engagement and attitude towards health promotion, competing food offerings, and varying student preferences. This review has identified a range of opportunities available to target these factors and supports the view that the education sector is a key domain to reach adolescents and improve nutrition and decision making around food and eating. A comprehensive approach that integrates environmental and behavioural strategies that engage adolescents in the development and/or implementation of initiatives, and evaluation can enhance success. Novel approaches such as integrating technology and student sampling of meals warrant further exploration. Higher quality pragmatic trials are required with longer term implementation and evaluation to build sustained change that will improve diet quality and ultimately benefit adolescents physical and mental health and wellbeing.

## Figures and Tables

**Table 1:** Eligibility criteria using the PICOS model.

	Inclusion Criteria	Exclusion Criteria
Population	Secondary (i.e., middle or high) schools that provide a routine main meal service (≥1 main meal/day) to most students (≥50%) on most days; students aged 10–19 years; generally well and independent of activities of daily living; upper-middle and high-income countries	Primary (i.e., elementary) schools; before or after school care; schools that only provide optional purchases that may supplement a meal provided from home or elsewhere; people aged <10 or >19 years; high-needs populations who are acutely or chronically unwell; selection of participants based on special nutritional needs (athletes, dance groups, high or at-risk of nutrient deficiency), specific disease state or weight status
Intervention	Single or multi-strategy nutrition-related interventions that target and modify the practices of the routine meal service; includes nudging strategies, policy implementation, menu changes, staff training; may vary in method, duration, or mode of delivery	Interventions that focus on components outside the routine meal service, e.g., introduce a new routine meal service, or target the total school food environment without specific routine meal service strategies
Comparison	Experimental studies with control or comparison groups (both classified as ‘controlled studies’ throughout review), not limited to parallel controls; single group experiments with comparison of before and after measurements	Experimental studies without control or comparison data; studies with comparative data but without an intervention (e.g., menu comparison across schools)
Outcomes	Objective or subjective measures of students’ food behaviours and dining experience that reflect a change in practice within the routine meal service; includes selection or consumption of a meal component (a food item, food group or nutrient), qualitative feedback, attitudes or satisfaction scores, knowledge, school meal program participation rates	Measurements that do not reflect student outcomes (e.g., menu assessment) or the impact of strategies targeting the routine meal service (e.g., dietary intake from total diet, anthropometric measures for interventions that include physical activity or classroom education unrelated to the routine meal service)
Study design	Randomised and non-randomised experimental trials, single group before-after studies; peer-reviewed publications; may be a pilot study	Non-peer-reviewed publications, reviews, observational studies, commentaries, editorials, conference proceedings, reports, PhD dissertations

PICOS, Population Intervention Comparison Outcome Study design; PhD, Doctor of Philosophy.

## Data Availability

Not applicable.

## Supplementary Materials

The following supporting information can be downloaded at: https://www.mdpi.com/article/10.3390/nu14173640/s1, Table S1: Risk of bias assessment by study [44,74,88,89,90,91,92,93,95,96,97,98,99,101,102,103,105,106,107,108,109,110,111,112,113,114,115,117,118,120,121,122,123,124,125]; Table S2: Outcome assessment, major findings and limitations of included studies [44,88,89,90,91,92,93,94,95,96,97,98,99,100,101,102,103,104,105,106,107,108,109,110,111,112,113,114,115,116,117,118,119,120,121,122,123,124,125]; Figure S1: Forest plot, proportion of students selecting a meal component [89,90,92,93,94,95,96,97,98,99,119]; Figure S2: (a) Forest plot, percent of serve consumed by students who selected a meal component [89,90,92,93,95,99,119]. (b) Forest plot, percent of serve consumed by students who selected a meal component (sensitivity analysis); Figure S3: (a) Forest plot, mean number of serves selected by students by meal component [88,90,91,98]. (b) Forest plot, mean number of serves selected by students by meal component (sensitivity analysis); Figure S4: Forest plot, mean number of serves consumed by students by meal component [89,90,91,98,119]; Figure S5: Effect direction plot for sensitivity analysis: quality and risk of bias [44,88,89,90,91,92,93,94,95,96,97,98,99,101,103,104,105,106,107,108,109,110,111,112,113,114,115,116,117,118,119,120,121,122,123,124,125]; Figure S6: Effect direction plot for sensitivity analysis: study design; Figure S7: Effect direction plot for sensitivity analysis: intervention duration [44,88,89,90,91,92,93,94,95,96,97,98,99,101,103,104,105,106,107,108,109,110,111,112,113,114,115,116,117,118,119,120,121,122,123,124,125]; Figure S8: Effect direction plot for sensitivity analysis: NOURISHING framework domains [44,88,89,90,91,92,93,94,95,96,97,98,99,101,103,104,105,106,107,108,109,110,111,112,113,114,115,116,117,118,119,120,121,122,123,124,125]; Figure S9: Effect direction plot for sensitivity analysis: NOURISHING framework action areas [44,88,89,90,91,92,93,94,95,96,97,98,99,101,103,104,105,106,107,108,109,110,111,112,113,114,115,116,117,118,119,120,121,122,123,124,125]; Figure S10: Effect direction plot for sensitivity analysis: student engagement [44,88,89,90,91,92,93,94,95,96,97,98,99,101,103,104,105,106,107,108,109,110,111,112,113,114,115,116,117,118,119,120,121,122,123,124,125]; Figure S11: Effect direction plot for sensitivity analysis: behaviour change communication [44,88,89,90,91,92,93,94,95,96,97,98,99,101,104,105,106,107,108,109,110,111,112,113,114,115,116,117,118,120,121,122,123,124,125].
