# Supplementary material for: The Impact of Modifying Food Service Practices in Secondary Schools Providing a Routine Meal Service on Student’s Food Behaviours, Health and Dining Experience: A Systematic Review and Meta-Analysis
Source: Nutrients. 2022 Sep 2;14(17):3640. doi: 10.3390/nu14173640 (PMC9460342; doi:10.3390/nu14173640)
Supplement: Supplementary file 1 [file nutrients-14-03640-s001.zip › nutrients-1887845-supplementary (1).pdf]

Table S1. Risk of bias assessment according to the Academy of Nutrition and Dietetics Quality Criteria Checklist [44,74,88–93,95–99,101–103,105–115,117,118,120–125].

#	Author	1. Was the research question clearly stated?	2. Was the selection of study subjects/patients free from bias?	3. Were study groups comparable?	4. Was method of handling withdrawals described?	5. Was blinding used to prevent introduction of bias?	6. Were intervention/therapeutic regimens/exposure factor or procedure and any comparison(s) described in detail? Were intervening factors described?	7. Were outcomes clearly defined and the measurements valid and reliable?	8. Was the statistical analysis appropriate for the study design and type of outcome indicators?	9. Are conclusions supported by results with biases and limitations taken into consideration?	10. Is bias due to study's funding or sponsorship unlikely?	Risk of bias rating: Neutral (Ø) Plus (+)
1	Askelson 2019 [117]	Yes	Unclear	Unclear	Unclear	No	Yes	Yes	No	Yes	Yes	Ø
2	Bean 2019 [102]	Yes	Yes	Unclear	No	Unclear	Yes	Yes	Yes	Yes	Yes	Ø
3	Bhatia 2011 [44]	Yes	Yes	Unclear	Yes	Unclear	Yes	Yes	No	Unclear	Yes	Ø
4	Boehm 2020 [96]	Yes	Unclear	Yes	No	Unclear	Unclear	Yes	Unclear	Yes	Yes	Ø
5	Bogart 2014 [88]	Yes	Yes	Yes	Yes	No	Yes	Yes	Yes	Yes	Yes	+
6	Bogart 2011 [109]	Yes	Yes	Yes	Yes	Yes	Yes	Yes	Yes	Yes	Yes	+
7	Bogart 2018 [110]	Yes	Yes	Yes	Yes	No	Yes	Yes	Yes	Yes	Yes	+
8	Chu 2011 [118]	Yes	Yes	Unclear	No	No	Yes	Unclear	Unclear	Yes	Yes	Ø
9	Cohen 2012 [89]	Yes	Yes	Yes	No	Unclear	Yes	Yes	Yes	Yes	Yes	+
10	Cullen 2007 [114]	Yes	Yes	Unclear	No	Unclear	Yes	Yes	Unclear	Yes	Yes	Ø
11	Cullen 2008 [103]	Yes	Yes	Yes	No	No	Yes	No	No	Yes	Yes	Ø
12	Cullen 2015 [90]	Yes	Yes	Yes	No	Unclear	Yes	Yes	Yes	Yes	Yes	+
13	D'Adamo 2021 [113]	Yes	Yes	Unclear	No	No	Yes	Yes	Yes	Yes	Yes	Ø
14	Elbel 2015 [107]	Yes	Yes	Yes	Unclear	Unclear	Yes	Yes	Yes	Yes	Yes	+

#	Author	1. Was the research question clearly stated?	2. Was the selection of study subjects/patients free from bias?	3. Were study groups comparable?	4. Was method of handling withdrawals described?	5. Was blinding used to prevent introduction of bias?	6. Were intervention/therapeutic regimens/exposure factor or procedure and any comparison(s) described in detail? Were intervening factors described?	7. Were outcomes clearly defined and the measurements valid and reliable?	8. Was the statistical analysis appropriate for the study design and type of outcome indicators?	9. Are conclusions supported by results with biases and limitations taken into consideration?	10. Is bias due to study's funding or sponsorship unlikely?	Risk of bias rating: Neutral (∅) Plus (+)
15	Ellison 1989 [115]	Yes	Yes	Yes	No	No	Yes	No	Yes	Yes	Yes	∅
16	Fritts 2019 [120]	Yes	Yes	Unclear	No	No	Yes	No	No	Yes	Yes	∅
17	Greene 2017 [91]	Yes	Yes	Yes	NA	No	Yes	Yes	Yes	Yes	Yes	+
18	Hackett 1990 [121]	Yes	No	Unclear	No	No	Yes	Yes	Yes	Yes	Unclear	∅
19	Hanks 2012 [122]	Yes	No	Yes	No	No	Yes	Yes	Yes	Yes	Yes	∅
20	Hanks 2013 [97]	Yes	No	Yes	No	No	Yes	Yes	Yes	Yes	Yes	∅
21	Hunsberger 2015 [123]	Yes	Yes	Unclear	Unclear	Unclear	Yes	Yes	Yes	Yes	Yes	+
22	Just 2014 [93]	Yes	Yes	Unclear	Unclear	Unclear	Yes	Yes	Yes	Yes	Yes	∅
23	Koch 2020 [124]	Yes	Yes	Yes	No	No	Yes	No	No	Yes	Yes	∅
24	Madden 2013 [105]	Yes	Yes	Unclear	Unclear	No	Yes	Yes	Yes	Yes	Yes	∅
25	McCool 2005 [108]	Yes	Yes	Unclear	No	No	Yes	No	No	Yes	No	∅
26	Pope 2018 [98]	Yes	Yes	Yes	Yes	No	Unclear	Unclear	Unclear	Yes	Yes	∅
27	Prell 2005 [101]	Yes	Unclear	No	No	Unclear	Yes	Unclear	No	Yes	Yes	∅
28	Prescott 2019 [99]	Yes	Unclear	Unclear	No	No	Unclear	Yes	Unclear	Yes	Yes	∅
29	Quinn 2018 [98]	Yes	Unclear	Unclear	Yes	Unclear	Yes	Yes	Yes	Yes	Yes	∅
30	Schwartz 2015 [92]	Yes	Unclear	Unclear	No	Unclear	No	Yes	Yes	No	Yes	∅

#	Author	1. Was the research question clearly stated?	2. Was the selection of study subjects/patients free from bias?	3. Were study groups comparable?	4. Was method of handling withdrawals described?	5. Was blinding used to prevent introduction of bias?	6. Were intervention/therapeutic regimens/exposure factor or procedure and any comparison(s) described in detail? Were intervening factors described?	7. Were outcomes clearly defined and the measurements valid and reliable?	8. Was the statistical analysis appropriate for the study design and type of outcome indicators?	9. Are conclusions supported by results with biases and limitations taken into consideration?	10. Is bias due to study's funding or sponsorship unlikely?	Risk of bias rating: Neutral (∅) Plus (+)
31	Sharma 2018 [106]	Yes	Yes	Unclear	No	No	Unclear	Unclear	No	Yes	Yes	∅
32	Turnin 2016 [112]	Yes	Yes	Yes	Yes	Unclear	Yes	Yes	Yes	Yes	Yes	+
33	Wansink 2015 [95]	Yes	Unclear	Yes	Unclear	No	Yes	Yes	Yes	Yes	Yes	∅
34	Wansink 2013 [111]	Yes	Yes	Unclear	Unclear	Unclear	Yes	Yes	Yes	Yes	Yes	∅
35	Witschi 1985 [125]	Yes	No	Yes	Unclear	No	Yes	Yes	Unclear	Yes	Yes	∅

QCC ratings: ∅ = neutral if validity questions 2, 3, 6 and 7 do not indicate that the study is exceptionally strong, + = positive if most of the validity questions are Yes including questions 2, 3, 6, 7 and at least one additional Yes. QCC, Quality Criteria Checklist; #, number.

Table S2. Outcome assessment, major findings and limitations of included studies [44,88–125].

Author, year of publication	Outcomes measured	Measurement tools	Tool scoring	Results	Major findings/conclusion	Limitations
Askelson et al. 2019 [117]	a. Lunchroom environment<br>b. Serves of (1) fruit, (2) veg, and (3) milk<br>c. Food service directors' experiences and perceptions of intervention	a. 21 item student lunchroom assessment tool targeting 5 areas, (1) milk), (2) fruit, (3) veg, (4) lunchroom atmosphere, (5) lunchroom staff. Data collection: students completed during lunch period (sample size NR) at B and FU (start and end of school year 2016 respectively)<br>b. Food service production records. Data collection: B (total servings 1 week in fall) vs. FU (total servings 1 week spring), approx. 4 months apart<br>c. Semi-structured interviews with food service directors (*n* = 6). Data collection: timing unclear	a. For each target area, students indicated if they identified assessment item questions as never, sometimes or always being true (score = 0, 1 or 2 respectively; total score range, 0–42)<br>b. Aggregate number of serves prepared less left-over serves; post-I vs. pre-I; binary outcome (increase or no increase)<br>c. Interviews recorded and transcribed; data coded by external social scientist not previously involved in intervention	a. 5 of 6 schools ↑ average score on assessment tool; total average score across schools ranged from 9–19 pre-I to 13–28 post-I<br>b. Aggregate serves of: (1) fruit: ↑ at 2 schools, (2) veg: ↑ at 3 schools, (3) milk: ↑ at 3 schools<br>c. Improved communication and relationships with students; humanisation of school workers; empowerment of students	Improvements to the school lunchroom in 5 evidence-based areas that promote healthy food choices; Actively involving the students and staff was a major benefit as evidenced	• Uncontrolled;<br>• Inadequate statistical analyses, results without SD;<br>• No measure of food consumption;<br>• Lunchroom assessment not a validated tool;<br>• Limited number of participating schools and mostly rural, prevents generalisability;
Bean et al. 2019 [102]	a. Sales of food and beverages: (1) fruits, (2) vegetables, (3) salad bar, (4) milk, (5) water	a. Cafeteria sales data during year 1 of study. Data collection: 2 months pre-staff training (pre-I, Feb-Mar 2015) vs. 2 months post-staff training (post-I, May-Jun 2015)	a. Difference in aggregate sales data pre-I vs. post-I collected for each category: (1) fruits, (2) vegetables, (3) salad bar, (4) milk, (5) water	a. NS difference for sales of (1) fruit, $p = 0.150$, (2) veg, $p = 0.245$, (3) salad bar, $p = 0.525$, (4) milk, $p = 0.245$, (5) water, $p = 0.986$.	Training cafeteria personnel improved adherence to Smarter Lunchroom principles, but without a SIG improvement in sales of fruit, veg, salad or milk (note: SSB sales ↓ in middle and high schools; $p = 0.024$ and 0.045 respectively)	• Uncontrolled;<br>• Short-term duration of initial follow-up;<br>• No measure of food consumed;<br>• Inability to examine sales data during sustainability year (year 2)

Author, year of publication	Outcomes measured	Measurement tools	Tool scoring	Results	Major findings/conclusion	Limitations
Bhatia et al. 2011 [44]	a. NSLP participation rate	a. School district data<br>Data collection: (1) school 1; pre-I, Jan-Mar 2009; post-I 14 months FU, Aug 2009-May 2010, (2) school 2; pre-I, Aug 2009-Jan 2010; post-I 4 months FU, Jan-May 2010, (3) school 3; pre-I, Aug 2009-Apr 2010; post-I 2 months FU, Apr-May 2010	a. Change in NSLP participation rates by subsidy status calculated for each school	a. Change in NSLP participation rates: (1) school 1, overall ↑ by 63%, (2) school 2, overall ↑ by 12%, (3) school 3, overall ↑ by 58%	Eliminating competitive a la carte offerings may ↑ NSLP participation among qualified low-income students. This effect may be mediated in part by reductions in stigma	• Uncontrolled;<br>• Inadequate statistical analyses, statistical significance not assessed;<br>• Limited study scope;<br>• Biases not described.
Boehm et al. 2020 [96]	a. Entrees (number served daily)<br>b. Cold and hot entrees (number served daily)<br>c. Share of entrees served with (1) veg, (2) fruit, or (3) milk (number served daily)	Cafeteria sales data provided by district food-service director for all outcomes measured<br>Data collection: B, 282 days across 3 schools (Sep 2013-Apr 2014); during-I, 50 days across 3 schools (after Apr 2014 spring break)	Average daily values of all outcome measures calculated from aggregate cafeteria sales data to compare I-schools vs. C-school, and pre-I vs. post-I (DID estimator; positive DID = ↑average daily value of outcome measure)	a. Choices School and Nudging School DID = 82.1 (SE 33.9; $p < 0.05$) and 107.4 (SE 28.2; $p < 0.01$) respectively<br>b. Choices School: DID = 69.7 (SE 22.0; $p < 0.01$) for cold entrees, NS for hot;<br>Nudging School: DID = 52.8 (SE 22.4; $p < 0.05$) and 54.3 (SE 24.4; $p < 0.05$) for cold and hot respectively<br>c. Choices School: DID = 0.06 (SE 0.02; $p < 0.01$) for veg, NS for fruit or milk;<br>Nudging School: NS difference for veg, fruit or milk	Removing competitive foods from a high school cafeteria (Choices school) was associated with a SIG ↑ in meal participation and share of entrees served with veg. Cafeteria nudging and marketing strategies (Nudging school; without changes to availability of competitive food) were also associated with higher school meal participation, however there was not a ↓ in competitive food sales	• Small study sample (n = 3 schools);<br>• No measure of food consumed;<br>• Short-term intervention;<br>• Majority of students (>95%) eligible for FRP meals, limits generalisability.
Bogart et al. 2011 [109]	a. Cafeteria attitudes<br>b. % student selection of: (1) fruit, (2) NSLP entrees	a. I-school student survey. Data collection: grade 7 students, 2 timepoints, (1) B, n = 425 (63% of all grade 7 students), and (2) post-I, 1 month FU, n = 399 students<br>b. School cafeteria records for all students attending the cafeteria (sample size NR). Data collection: pre-I	a. Survey question 'I believe eating in the cafeteria is …' measured by a three 7-point semantic differential scale (unsatisfying/satisfying; bad/good; unhealthy/healthy; midpoint = neither); survey identified groups	a. Within groups: cafeteria attitudes improved over time among peer advocates ($p = 0.003$), stable for non-peer (NS; $p = 0.34$);<br>Between groups: improvement in cafeteria attitudes among peer advocates > non-peer advocates (b = 0.71; $p < 0.001$)<br>b. Within groups: B vs. post-I: selection of fruit ↑18% in I-school ($p < 0.001$) and ↓5% in C-school (p	Compared with non-peer advocates, peer advocates appeared to benefit more from the intervention;<br>CBPR can help to develop programs that are based on community needs and priorities identified by community members	• Small study sample (n = 2 schools);<br>• Non-randomised comparison school, limits validity and examination of causality;<br>• No measure of food consumed;

Author, year of publication	Outcomes measured	Measurement tools	Tool scoring	Results	Major findings/conclusion	Limitations
		(I-school = 40 days; C-school = 48 days), during-I (I and C-schools both 26 days) and post-I (I-school = 30 days; C-school = 26 days)	as peer advocates vs. non-peer<br>b. Number of students per day, (1) visiting cafeteria, (2) selecting fruit, and (3) selecting healthier entrees to determine proportions (student sample sizes not reported)	< 0.001); healthy entrees ↑2% in I-school ($p < 0.001$) and NS difference for C-school;<br>Between schools: B vs. post-I: I-school change in selection of fruit and healthier entrees SIG vs. C-school ($p < 0.001$)		• Survey created for pilot study, not validated;<br>• Inadequate staffing in I-school compared to C-school.
Bogart et al. 2014 [88]	a. Cafeteria and tap water attitudes<br>b. Number of serves selected of (1) fruit, (2) veg	a. Student survey. Data collection: grade 7 students, 2 timepoints, (1) B, $n = 3211$ (80% of all grade 7 students), and (2) post-I, 1 month FU, $n = 2997$ (75% of all grade 7 students)<br>b. School cafeteria records for all students attending the cafeteria (sample size NR). Data collection: sales data for each day of the intervention semester/school for pre-I (5 weeks), during-I (5 weeks) and post-I FU (5 weeks)	a. Analysed at student level; researcher constructed questions: (1) cafeteria attitudes measured with mean of 2 items for "I believe eating in the cafeteria is…" with responses 1 = unsatisfying to 7 = satisfying, and 1 = bad for my health to 7 = good for my health; (2) tap water attitudes measured with "on a scale of 1–10 how to you feel about drinking water?" with responses 0 = very negatively to 10 = very positively<br>b. Aggregate cafeteria data of fruit and veg serves per day divided by number of students in attendance to derive proportions	a. Within groups: pre-I to FU, cafeteria and tap water attitudes less positive in C-schools ($p < 0.001$), remained similar in I-schools (NS).<br>Between groups: pre-I to FU, I-effect on cafeteria and tap water attitudes both positive (b(SE) = 0.13(0.05); $p < 0.05$) and (b(SE) = 0.20(0.09); $p < 0.05$) respectively<br>b. (1) fruit serves: within groups; I-schools, pre-to-during-I, ↑ 0.09 (SE 0.02, $p < 0.001$), pre-to-post-I remained stable (NS); C-schools, pre-to-during-I, remained stable (NS), pre-to-post-I ↓ 0.05 ($p<0.05$); between groups; I-effect, 15.3% more fruit served than parallel changes in C-schools. (2) veg serves: within and between groups; NS across I and C-schools	SNaX led to ↑ NSLP participation, changes in student body cafeteria serving patterns and attitudes in a large school district;<br>Multi-level school-based interventions may promote healthy adolescent dietary behaviours<br>Fruit effects not sustained beyond intervention period suggesting a need for permanent environmental changes, and that cafeterias need to sustain a variety of produce	• Unable to isolate aspects of SNaX that led to behaviour changes;<br>• No measure of food consumed;<br>• Whole-school effects may have been due to changes amongst 7th grade students who received largest I-dose.
Bogart et al. 2018 [110]	a. Number of serves selected of: (1) fruit, (2) veg, (3) entrée<br>b. Student advocate rating of each SNaX component<br>c. Student advocate and staff	a. School cafeteria data<br>b. Student advocate survey. Data collection: post-I (end of 2014–2015 school year), $n = 187$ student advocates<br>c. Interviews and focus groups. Data collection:	a. Aggregate cafeteria data of serves per day of fruit, veg and NSLP lunches divided by number of students attending cafeteria per day<br>b. Likert scale responses to rate each SNaX	a. Between schools, DID estimates for: (1) fruit serves, b(SE) = 2.04(1.55), NS, (2) veg serves, b(SE) = 2.65(3.73), NS, (3) NSLP lunches, b(SE) = 3.90(2.05), NS<br>b. Student responded excellent or very good rating: 91% for program in general, 73% for posters, 72%	Fruit and veg and cafeteria servings overall did not ↑ in SNaX schools;<br>Core program components must be implemented with fidelity to the original	• Comparisons schools not randomly selected, limits validity test for effectiveness;<br>• No measure of food consumed;

Author, year of publication	Outcomes measured	Measurement tools	Tool scoring	Results	Major findings/conclusion	Limitations
	perceptions of SNaX	post-I, n = 46 staff interviews, n = 154 student advocate focus group participants	component (1 = poor to 5 = excellent, with an option 'was not aware/did not participate')<br>c. Interviews and focus groups (recorded and transcribed) to understand staff and students overall impressions of SNaX and its activities	for videos, 75% for assembly, 86% for club sessions, 80% for lunchtime activities, 89% fir hydration station, 59% for website, 52% for apps<br>c. Positive feedback: students liked taste testing, giveaways, awareness of healthy vs. unhealthy; videos helped communicate messages. Less favourable feedback: student advocates had some difficulty engaging fellow students, hydration station required dedicated staff to supervise, labour-intensive, varying levels staff engagement	protocol for effectiveness	• Inconsistent implementation between original protocol and disseminated program, compromises effectiveness.
Chu et al. 2011 [118]	a. Attitudes: student acceptance of wholegrain and refined tortillas according to sensory attribute ratings (1) overall liking, (2) taste, (3) colour, (4) softness	a. Student survey<br>Data collection: surveys distributed to students during school meal on 2 occasions when whole-wheat or refined tortillas were served, total n = 735 surveys collected (n = 359 for refined tortillas, n = 85 for 66% whole-wheat, n = 291 for 100% whole-wheat)	a. Students were asked to rate sensory attributes on 9-point hedonic scales (higher rating indicates higher acceptance) for (1) refined wheat tortillas, (2) 66% white whole-wheat, (3) 100% white whole-wheat tortillas	a. (1) overall liking: similar for 66% whole-wheat vs refined (6.3 vs 6.8, p = 0.68), lower for 100% whole-wheat vs refined (5.8 vs 6.8, p = 0.01), (2) taste: similar for 66% whole-wheat vs refined (6.0 vs 6.8, p = 0.17), lower for 100% whole-wheat vs refined (5.6 vs 6.8, p = 0.002), (3) colour: lower for 66% whole-wheat vs refined (4.8 vs 6.2, p = 0.007), lower for 100% whole-wheat vs refined (5.5 vs 6.2, p = 0.14), (4) softness: similar for 66% whole-wheat vs refined (6.5 vs 6.8, p = 0.99), lower for 100% whole-wheat vs refined (5.6 vs 6.8, p = 0.002)	Novel whole-wheat products are acceptable to adolescents; Substituting refined grain with whole-wheat options represents a viable and relatively straightforward strategy to encourage and increase consumption of wholegrain products in schools	• Schools not randomly selected, limits generalisability;<br>• Food service staff were not provided with instructions for whole-wheat products, limits implementation fidelity.
Cohen et al. 2012 [89]	a. % student selection of: (1) wholegrains, (2) entrees, (3) sides, (4) milk, (5) fruit, (6) veg	a. Observation<br>b. Tray waste assessment<br>c. Tray waste assessment<br>Data collection: 2 consecutive days per school	a. Researcher at cash register recorded tray number, food selection, sex<br>b. % serve consumed: pre-lunch weight of menu items each study day	a. Wholegrain selection: I > C (85.7% vs. 34.7%; $p < 0.02$); NS differences for selection of entrees, sides, milk, fruit or veg<br>b. I and C-schools consumed similar amounts of food overall (61.6%	Chef-based model useful to enhance diet quality and palatability without ↓ food eaten	• Schools not randomly selected;<br>• Pre-I consumption data could not be assessed;

Author, year of publication	Outcomes measured	Measurement tools	Tool scoring	Results	Major findings/conclusion	Limitations
	b. % of serve consumed of: (1) overall meal, (2) side dish<br>c. Number of serves consumed: (1) veg, (2) fruit, (3) wholegrains	during spring 2009 (Mar-May 2009)	(average of 10 random samples of each portioned food; g) less post-lunch weight of leftover item (g) divided by pre-lunch weight multiplied by 100	and 57.3%; $p = 0.63$); side dish: I > C (74.6% vs. 29.2%; $p < 0.0001$), therefore > wholegrain consumed<br>c. Number of serves consumed: (1) veg: I > C (0.54 vs. 0.18; $p = 0.01$), (2) fruit: NS, (3) wholegrains: NS		• Only low-income middle schools included;<br>• Consumption evaluated on only 2 days at each school.
Cohen et al. 2013 [119]	a. Intake of nutrients: (1) food energy, (2) fibre, (3) total fat, (4) sat fat, (5) iron, (6) calcium, (7) vitamin C, (8) vitamin A<br>b. % of serve consumed of: (1) entrée, (2) fruit, (3) veg, (4) milk<br>c. % of serve wasted of: (1) entrée, (2) fruit, (3) veg, (4) milk	a. Assessment of menu components to estimate average nutrients<br>b. Tray waste assessment<br>c. Tray waste assessment	a. Average daily nutrients consumed = % students selected food item × average amount consumed<br>b. After lunch, trays collected and leftover food items weighed; % serve consumed = pre-lunch weight (per Cohen 2012 [89]) less leftover weight divided by pre-lunch weight multiplied by 100<br>c. % serve wasted: post-lunch weight of leftover item (g) divided by pre-lunch weight (per Cohen 2012) multiplied by 100	a. I-school students consumed more fibre, vitamin C and vitamin A, and less sat fat (all $p < 0.05$); NS diffs for food energy, total fat, iron and calcium<br>b. % serve consumed: (1) entrée: NS difference I vs. C (79.7% and 83.5%), (2) fruit: NS difference I vs. C (45.2% and 62.8%), (3) veg: I > C (40.2% and 10.8%, $p < 0.05$), (4) milk: NS difference, I vs. C (77.6% and 72.3%)<br>c. I-school students discarded less veg than C-school ($p < 0.05$); NS differences for entrees, fruit, milk; on average across all schools, students discarded 19% entrees, 47% fruit, 25% milk, 73% veg	I vs. C: students accepted healthier foods, wasted fewer veg, consumed more fibre, vitamin C and vitamin A, suggests ↑ food quality and ↓ meal waste is feasible; Schools require additional funding for higher-quality foods and additional staff training and support to produce more palatable meals and therefore reduce waste and ↑ nutrient consumption; substantial quantities of lunch foods are discarded.	• As per Cohen 2012 [89]

Author, year of publication	Outcomes measured	Measurement tools	Tool scoring	Results	Major findings/conclusion	Limitations
Cullen et al. 2007 [114]	a. Goal achievement for NSLP goals: (1) ≥3 fruit and veg menu items per day, (2) ≥10 different types of fruit and veg items over each 3-week period, and (3) ≥2 lower fat entrees per week<br>b. Number of serves selected of: (1) fruit, (2) veg<br>c. Student and staff feedback	a. Questionnaires<br>b. School food production and sales records; data collection at B (1 week) and FU (6 weeks)<br>c. Focus groups (*n* = 6) with grade 6 students and interviews with foodservice directors, managers and principals at each school. Data collection: at study end	a. Successful goal achievement if achieving ≥ 75% of goals not met at baseline<br>b. Daily count of fruit and veg served with NSLP meals per student enrolled in the school per day<br>c. Process of qualitative data analysis not reported	a. At week 6, all schools met all goals; goal achievement 100%<br>b. Across all schools: (1) mean fruit serves/student/day ↑ from 0.23 to 0.42, (2) mean veg serves/student/day ↑ from 0.65 to 0.79<br>c. Students noticed ↑ variety of fruit and veg; food service managers and directors were supportive of changes; principals noted positive comments from teachers, parents and staff	School cafeteria changes can be implemented in the short-term and are acceptable to staff and students;<br>Future work should implement longer-term interventions, assess cost issues and measure dietary intake	• Uncontrolled;<br>• Small study sample (*n* = 6 schools);<br>• Short-term intervention (*n* = 6 weeks);<br>• No measure of food consumed;<br>• Inadequate statistical analyses, results without statistical significance or SD.
Cullen et al. 2008 [103]	a. Intake of nutrients: (1) food energy, (2) protein, (3) % kJ from total fat and sat fat, (4) fibre, (5) vitamin A, (6) vitamin C, (7) iron, (8) calcium, (9) sodium<br>b. Serves consumed of: (1) fruit or juice, (2) veg<br>c. Number NSLP meals served and sold (FRP and full price meals)<br>d. % students receiving lunch meal by eligibility status: (1) free, (2) reduced-price, (3) full-price	a. Student self-reported lunch food records; data collection: Pre-I, Sep 2001 to May 2002, *n* = 2671 food records; post-I 1 year FU from policy implementation (fall of 2004), Sep 2005 to May 2006, *n* = 10234 food records; data collected 3–5 days/week from 1–2 tables of students at each lunch period<br>b. As above<br>c. Not described	a. Data collectors instructed convenience sample of assenting students how to complete lunch records listing each food item selected, number of servings eaten, source of each food item (NSLP, snack bar, home, vending or other); nutrient assessment using Nutrition Data System (NDS, v4.2)<br>b. Serves of food groups assessed using NDS<br>c. Not described<br>d. Not described	a. Mean daily nutrient intake (% from NSLP): (1) food energy: ↑ from 2646 to 2990 kJ, $p < 0.025$, (from 53 to 83%), (2) protein: ↑ from 22 to 28 g, $p < 0.025$, (from 63 to 93%), (3) % kJ from total fat and sat fat: NS, (4) fibre: ↑ from 3.6 to 4.6 g, $p < 0.025$, (from 62 to 91%), (5) vitamin A: ↑ from 151 RE to 220 RE, $p < 0.025$, (from 72 to 94%), (6) vitamin C: ↑ from 14 to 27mg, $p < 0.025$, (from 68 to 93%), (7) iron: ↑ from 3.4 to 4mg, NS, (from 58 to 89%), (8) calcium: ↑ from 292 to 454 mg, $p < 0.025$, (from 66 to 94%), (9) sodium: ↑ from 1020 to 1237 mg, $p < 0.025$, (from 58 to 91%)<br>b. Mean daily lunch serves consumed per student (% NSLP) of: (1) fruit or juice: ↑ from 0.32 to 0.45, NS, (from 87 to 94%), (2) veg: ↑ from 0.29 to 0.89, $p < 0.025$, (from 83 to 99%)	After implementation of the nutrition policy students significantly increased consumption of protein, fibre, vitamins A and C, calcium and sodium, and servings of vegetables;<br>More than 85% of the healthful food selections were from the NSLP meal;<br>Limiting snack bar offerings and encouraging students to select a reimbursable meal increased number of students receiving a NSLP meal by 98%	• Uncontrolled;<br>• Small study sample (*n* = 3 schools);<br>• Self-reported student data;<br>• Individual students not followed longitudinally, therefore students potentially completed multiple assessments;<br>• Bias associated with social clustering at lunch tables;<br>• Analyses did not account for potential clustering effect by school;<br>• No process evaluation to measure implementation fidelity.

Author, year of publication	Outcomes measured	Measurement tools	Tool scoring	Results	Major findings/conclusion	Limitations
				c. Total NSLP lunch meals served ↑ by 98% (205 547 to 407 063); statistical significance not assessed<br>d. % increase in students receiving lunch: free +77%, reduced-price +127%, full-price +143%		
Mendoza et al. 2010<br>[104]<br>(same data as Cullen 2008 [103])	a. Lunch meal % energy from: (1) NSLP entrée, (2) fruit, (3) veg	a. Student self-reported lunch food records; data collection: Pre-I, Sep 2001 to May 2002, *n* = 2616 food records; post-I 1 year FU from policy implementation (fall of 2004), Sep 2005 to May 2006, *n* = 10172 food records; data collected 3–5 days/week from 1–2 tables of students at each lunch period	a. As per Cullen 2008 [103] for scoring of lunch food records using NDS (v4.2)	a. Total lunch meal % energy increased significantly for (1) NSLP entrée, 30.3 (SD 19.6) to 37.5% (SD 20.2), (2) fruit, 4.1 (SD 8.4) to 6.5% (SD 10.1), (3) veg, 7.5 (SD10.2) to 10.3% (SD 12.9); all $p < 0.00625$	All schools combined, lunch meal % energy contribution significantly increased for NSLP entrée, veg and fruit	• As per Cullen 2008 [103]
Cullen et al. 2015<br>[90]	a. % student selections: (1) fruit, (2) total veg, (3) starchy veg, (4) legumes, (5) grains, (6) wholegrains, (7) protein foods, (8) milk<br>b. % of serve consumed of food groups as above<br>c. Amount selected of food groups as above<br>d. Amount consumed of food groups as above<br>e. Calories selected and consumed from food groups as above	a. Observation<br>b. Tray waste assessment<br>c. Diet analysis software (Nutrition Data System for Research; NDSR)<br>d. NDSR as above<br>e. NDSR as above<br>Data collection: observations conducted 1 day/week/per school during the semester (10 approx. 15 days), for *n* = 427 student observations across I and C-schools (*n* = 212 and 215 respectively)	a. Observations conducted unobtrusively from a distance; foods selected during lunch recorded on pre-printed observation checklist with all menu items listed for students with a reimbursable NSLP meal<br>b. Foods consumed recorded on observation checklist using quarter waste method (0, ¼ , ½ , ¾ , or all)<br>c. For each observation checklist completed, foods selected were entered into NDSR by a trained dietitian to calculate amounts selected	a. % students selecting: (1) fruit: I > C (45% and 21%, $p < 0.001$), (2) total veg: I > C (52% and 41%, $p < 0.05$), (3) starchy veg: I > C (39% and 27%, $p < 0.01$), (4) legumes: I > C (9% and 4%, $p < 0.05$), (5) grains: NS difference I vs. C (100% and 99%), (6) wholegrain: NS difference I vs. C (49% and 52%), (7) protein foods: I > C (100% and 97%, $p < 0.01$), (8) milk: NS difference I vs. C (76% and 74%)<br>b. % serve consumed: (1) fruit: NS difference I vs. C (76% and 78%), (2) total veg: NS difference I vs. C (53% and 52%), (3) starchy veg: NS difference, (4) legumes: NS difference, (5) grains: I < C (83% and 100%, $p < 0.001$), (6) wholegrains: I < C (67% and 81%, $p < 0.05$), (7) protein foods: I < C	More I-school students selected fruit and veg at lunch and consumed them compared to C-school students; Generally low consumption of fruit, veg, and wholegrains by students is a concern<br>Future studies with larger and more diverse student populations are warranted	• School district area only had 26% students eligible for FRP meals, limits generalisability;<br>• Study conducted in 12 schools in Houston area, limits generalisability;<br>• Marketing strategies only available in I-schools.

Author, year of publication	Outcomes measured	Measurement tools	Tool scoring	Results	Major findings/conclusion	Limitations
			d. As above to calculate amounts consumed<br>e. As above to calculate intake of calories	(84% and 96%, $p < 0.01$), (8) milk: NS difference I vs. C<br>c. Amount selected: (1) fruit: I > C ($p < 0.001$), (2) total veg: I > C ($p < 0.01$), (3) starchy veg: I > C ($p < 0.001$), (4) legumes: I > C ($p < 0.01$), (5) grains: NS difference I vs. C, (6) wholegrains: I < C ($p < 0.05$), (7) protein foods: NS difference I vs. C, (8) milk: NS difference I vs. C<br>d. Amount consumed: (1) fruit: I > C ($p < 0.001$), (2) total veg: I > C ($p < 0.01$), (3) starchy veg: I > C ($p < 0.05$), (4) legumes: I > C ($p < 0.01$), (5) grains: I < C ($p < 0.05$), (6) wholegrains: I < C ($p < 0.01$), (7) protein foods: NS difference I vs. C, (8) milk: NS difference I vs. C<br>e. Calories selected: NS difference I vs. C (612 and 599); calories consumed: I < C (520 and 571, $p < 0.001$)		
D'Adamo et al. 2021 [113]	a. Amount consumed of NSLP veg<br>b. Veg consumption during semester without student-led advocacy (year 1) vs. semester with student-led advocacy (year 2)	a. Tray waste assessment, weighing method<br>b. Tray waste assessment, weighing method<br>Data collection: anonymous collection of all student lunch trays for 8 weeks (4 weeks in Nov-Dec, 4 weeks in Apr-May); during each 4-week period, typical veg (C-group) was served for 2 weeks followed by spices and herbs veg (I-group) for 2 weeks; $n = 4570$ total tray waste observations (I-group $n = 2160$, C-group $n = 2410$)	a. One veg recipe offered each day; 1 serve per student per day in separate container; veg intake = estimated mean veg served weight each day (mean weight of 10 separate servings) less weighed plate waste (by research staff) using OHAUS Gold Series SPJ601 scales<br>b. As above	a. Mean daily veg consumed: I > C, 53g vs 44.8g (18.2% increase, 8.22 g, $p < 0.0001$)<br>b. Total veg consumed: without student led advocacy, spices and herbs recipe 15.4% higher than typical recipe; with student led advocacy, spices and herbs recipe 27.2% higher than typical recipe (NS, $p = 0.08$)	The addition of spices and herbs to veg in the NSLP was feasible and associated with small increases in veg consumed. The magnitude of the impact of spices and herbs on veg consumed was dampened by heterogenous effects that varied greatly by specific veg (7 different NSLP veg recipes)	• Small study sample ($n = 1$ school);<br>• Students not randomised to groups;<br>• Real-world setting led to variability in preparation and serving of veg<br>• Lack of 'liking survey' to accompany consumption data.

Author, year of publication	Outcomes measured	Measurement tools	Tool scoring	Results	Major findings/conclusion	Limitations
Elbel et al. 2015 [107]	a. Water-drinking behaviours at lunchtime<br>b. Cafeteria manager observations of students' interaction with water jets	a. Student survey to grade 8 (middle school) and grade 11 (high school) students, total n = 1759 surveys collected; data collected pre-I (Nov 2010) and follow up (Mar 2011)<br>b. Structured interviews with cafeteria manager in each I-school	a. Consumption frequency questions (I and C-school students) adapted from Youth Physical Activity and Nutrition Survey: frequency of water consumption the previous day, what they usually drank at lunch at school, did they agree or disagree that they liked the taste of tap water, safe to drink, healthy. Additional questions for I-schools: did students notice water jets, how often they used it, do they drink more water<br>b. Interviews conducted by trained interviewer; quantitative and open-ended questions including observations of student interaction with water jets	a. Middle/high school students drinking water at lunch on most days ↑ in both I and C-schools (p < 0.001) but change > in I-schools (from 27.6% to 42.5%), overall program impact 8.2 percentage point ↑ (p = 0.058); NS change in average glasses of water drunk the day before each survey; students drinking milk at lunch on most days ↓ slightly in I-schools (32% to 29%, p < 0.001), stable in C-schools (NS), program impact NS; 88% of middle and high school students noticed water jet in cafeteria, of which 12 approx. 64% said they used it every day or occasionally, 54% liked the taste, 50% indicated they drank more water, 85% indicated it was safe to drink<br>b. All managers noted that students were drinking more water since installation; most noted that >50% of students were accessing water jet	Water jets in school cafes at lunchtime, and without promotional activities, significantly increased students drinking water at lunch	• No direct measure of actual water and milk consumption;<br>• NYC has policy of no SSBs or other competitive foods in public schools, limits generalisability;<br>• Student survey data come from a subset of participating schools, potential recall bias

Author, year of publication	Outcomes measured	Measurement tools	Tool scoring	Results	Major findings/conclusion	Limitations
Ellison et al. 1989a [115]	a. Intake of nutrients: sodium (Na/mEq)<br>b. Acceptability of reduced-sodium foods	a. 24-hour food recall. Data collection: food diaries completed 1 day/week over 12 weeks (I and C schools): baseline, 6 weeks at start of year; during-I, 2 weeks in winter and 4 weeks in spring<br>b. Palatability survey. Data collection: students completed survey as they left dining hall, n = 1700 surveys	a. Dietary analysis by trained dietitians using Food Finder program: recipes and commercially prepared foods added to determine sodium and fat values<br>b. Students blindly tested both regular and modified products; 5-point Likert scale, (range 1 = much worse to 5 = much better than product previously served)	a. 51% less sodium in food served; I-group: mean sodium intake ↓ 13 approx. 15-20% ($p < 0.001$)<br>b. Average ratings almost identical; ↓ sodium 3.26 vs. regular 3.25	School food departments can readily be taught to purchase & prepare foods which contain considerably less sodium & less SFA than usual products; Food modifications were very acceptable to students and faculty	• Self-reported student data (dietary intake), possible measurement bias, limits validity
Ellison et al. 1989b [100]	a. Health status: systolic blood pressure (SBP) and diastolic blood pressure (DBP)	a. Student self-measure using vital signs monitor (Dinamap 845); data collection: B, at start of school year; post-I at end of school year	a. Measures during lab class each week; 3 measures/occasion; 2 and 3 averaged and recorded; baseline/subject taken as average of recordings in 4-weeks at start of year; follow-up/subject taken as average of recordings in 6-weeks at end of year	a. I-effect on SBP ↓1.7 mgHg (95% CI −0.6, −2.9; $p = 0.003$); DBP ↓1.5 mgHg (95% CI: −0.6, −2.5; $p = 0.002$)	Modest changes in sodium intake were sufficient to have a significant impact on BP	• Self-reported student data (blood pressure), potential measurement bias, limits validity.
Ellison et al. 1990 [116]	a. Intake of nutrients: saturated fat (g)<br>b. Polyunsaturated-to-saturated fat ratio (P/S ratio)	a. 24-hour food recall. Data collection: food diaries completed as per Ellison 1989a [115]<br>b. As above	a. Dietary analysis by trained dietitians using Food Finder program: recipes and commercially prepared foods added to determine fat values<br>b. Calculated from nutrient data using Food Finder program	a. I-effect on saturated fat intake: males ↓20% (95% CI: −29, −11); females ↓23% (95% CI: −33, −13)<br>b. I-effect on P/S ratio: males ↑81% (95% CI: 60, 102); females ↑47% (95% CI: 31, 62)	The modification of 13 approx. ⅔ of the fat-containing food products served in the schools' dining halls ↓ total saturated fat by > 20% & resulted in a P/S ratio approaching 1.0, the level currently recommended by most health agencies (at time of publication)	• As per Ellison 1989a [115] above;<br>• Statistical significance not assessed

Author, year of publication	Outcomes measured	Measurement tools	Tool scoring	Results	Major findings/conclusion	Limitations
Fritts et al. 2019 [120]	Phase 1 (I vs. C):<br>a. % student selection of veg (8 veg × 2 conditions)<br>b. Amount consumed of veg (8 veg × 2 conditions)<br>c. Willingness to eat again<br>Phase 2 (before-after):<br>a. % student selection of veg (2 veg recipes)<br>b. Amount consumed of featured veg recipes: (1) Dillicious Broccoli, (2) Fiesta Black Beans and Corn<br>c. Willingness to eat again	Phase 1 and 2:<br>a. Researcher observation. Data collection: 2 days for each veg recipe (1 veg + 1 condition per day); phase 1, *n* = 32 days; phase 2, *n* = 8 days; range of students receiving lunch each day, *n* = 569–670 (phase 1, Mar-May 2017), *n* = 507–637 (phase 2, pre-I Oct 2017, post-I Dec 2017)<br>b. As above<br>c. Student placement of returned veg containers according to signage	a. Researcher observation of the number of veg bowls returned to tray racks or discarded<br>b. Pre-weighed veg cups less post-weight from all veg cups returned after each lunch period<br>c. Students returned veg cups to tray racks that were divided into sections with signage: (1) 'would eat again', or (2) 'would not eat again'	Phase 1 (I-group, seasoned vs. C-group, lightly salted):<br>a. Veg selection: middle school, I > C for 5 of 8 veg varieties (NS); high school, I > C for 4 of 8 veg varieties (NS)<br>b. Veg consumption: middle school, I > C for 6 of 8 veg varieties; high school, I > C for 2 of 8 veg varieties<br>c. Willingness to eat again: middle school, I < C for all 8 veg varieties; high school, I > C for 3 of 8 veg varieties<br>Phase 2 (before-after assessment):<br>a. Veg selection: (1) broccoli, middle school ↑0.2% (from 6.2% to 6.4%); high school ↑0.3% (from 6.9% to 7.2%), (2) black beans, middle school ↓0.3% (from 1.6% to 1.3%); high school ↓0.8% (from 2.1% to 1.3%)<br>b. Veg consumption: (1) broccoli, ↑10.8g (from 130.1 to 140.9, NS p = 0.06), and (2) black beans, no effects, NS<br>c. Willingness to eat again: (1) broccoli, ↑'d proportion of students who 'would eat again' (p = 0.003), (2) black beans, NS effect	In a short-term intervention, herbs and spices did not produce robust increases in school lunch veg consumption, however with repeated exposure, flavours may become accepted.	• Phase 1: students not randomised to groups;<br>• Phase 2: uncontrolled

Author, year of publication	Outcomes measured	Measurement tools	Tool scoring	Results	Major findings/conclusion	Limitations
Greene et al. 2017 [91]	a. Number of serves selected of: (1) fruit, (2) veg, (3) white milk<br>b. Number of serves consumed of: (1) fruit, (2) veg, (3) white milk	a. Researcher observation<br>b. Tray waste observations by trained field researchers (not linked to individual students)<br>Data collection: pre-I, *n* = 5 days per school (*n* = 4654 tray observations, Feb 2014); during-I *n* = 4 days per school (*n* = 3098 tray observations, Mar-Apr 2014); total *n* = 7752 tray observations (I-schools, *n* = 4139; C-schools, *n* = 3613); observers recorded tray waste of students who ordered a school lunch, data not linked to individual students therefore unknown how often the same student was observed	a. Researcher observed all food items on student trays, each item measured as a unit or serving (e.g. 1 fruit serve = ½ cup diced fruit or 1 whole apple)<br>b. Amount consumed per food item = researcher observed typical serving of each food item prior to the start of lunch period less visual estimate of food remaining using quarter-waste method (0%, 25%, 50%, 75% or 100% left on tray)	a. Selection of (1) fruit: I-schools, ↑ by 36% (0.59 to 0.80 serves, $p < 0.001$); C-schools ↓ by 22% (0.64 to 0.50 serves, $p < 0.001$); DID I-effect, ↑ 36% ($p < 0.001$), (2) veg: I-schools, ↑ by 46% (0.67 to 0.98 serves, $p < 0.001$); C-schools ↑ by 10% (0.81 to 0.89 serves, $p < 0.004$); DID I-effect, NS ($p = 0.074$), (3) milk: I-schools, ↑ by 36% (0.10 to 0.14 serves, $p < 0.001$); C-schools ↓ by 25% (0.28 to 0.21 serves, $p < 0.000$); DID I-effect, NS<br>b. Consumption of (1) fruit: I-schools, ↑ by 14% (0.73 to 0.83, $p < 0.001$); C-schools ↓ by 16% (0.85 to 0.71, $p < 0.001$); DID I-effect, ↑ 23% ($p = 0.017$), (2) veg: I-schools, ↑ by 51% (0.57 to 0.86, $p < 0.001$); C-schools, ↑ by 34% (0.80 to 1.07, $p < 0.001$); DID I-effect, NS ($p < 0.10$), (3) milk: I-schools, stable (NS); C-schools, ↓ by 7% (NS, $p = 0.006$); DID I-effect, NS	Implementing specific fruit-promoting strategies ↑ selection and consumption of fruits, and partially impacted the selection and consumption of veg and milk; Findings add to previous positive results of Smarter Lunchrooms research, and have greater generalisability given the diversity of the school sample	• Average of 9 days of observations per school;<br>• No measurement of individual student data;<br>• High % of economic disadvantage in participating schools (≥49%), limits generalisability

Author, year of publication	Outcomes measured	Measurement tools	Tool scoring	Results	Major findings/conclusion	Limitations
Hackett et al. 1990 [121]	a. Meal program participation rate	a. Questionnaire	a. Self-report identical surveys at B (Jul 1987) and FU (Jul 1988); students lunch meal recorded as either (1) school meal, (2) packed lunch, (3) other arrangement, (4) no lunch	a. School meal participation: (1) 'Dish of day free-choice' I-schools, ↓10% (↓19% in affluent catchment school, ↓8% in less-affluent catchment school), and (2) '2 course meal fixed price' I-schools, ↓1% (↓7% in affluent catchment school, ↑5% in less-affluent catchment school)	School meal participation rate reduced in 3 out of 4 schools; Proportion of students eating school meals varied considerably between schools; no apparent relationship between school meal uptake and system (free choice vs. fixed price); School meals did not lose customers to 'packed lunches'; the number of students making 'other arrangements' out of school ↑; Attitudes towards school meals and the environment may be of paramount importance	• Survey questions related to 1 day at each school (repeat cross-sectional design);<br>• Inadequate statistical analysis;<br>• Study compared 2 slightly varied marketing campaigns dependent on the school meal system, therefore no comparison with a control group.

Author, year of publication	Outcomes measured	Measurement tools	Tool scoring	Results	Major findings/conclusion	Limitations
Hanks et al. 2012 [122]	a. Number of serves selected of: (1) healthier foods, (2) less healthy foods, (3) white milk, (4) flavoured milk<br>b. Amount consumed of: (1) healthier foods, (2) less healthy foods, (3) white milk, (4) flavoured milk	a. Report cards<br>b. Researcher tray waste observations, weighing method<br>Data collection: field researchers collected data from as many students as possible during 3 lunch periods each day (*n* = 1084 total observations); pre-I, *n* = 2 days (3 Feb and 25 Mar 2011, *n* = 362 and 240 observations respectively); post-I, *n* = 2 days (12 and 27 May 2011, *n* = 262 and 220 observations respectively)	a. Students self-reported lunch items selected on report card<br>b. Amount consumed = pre-weighed food item offered each day less weighed leftover portion of each food item on student lunch trays	a. Mean serves selected per student of: (1) healthier foods, 0.66 to 0.79 (18.8% increase, 0.13 serves, p = 0.00), (2) less healthy foods, 0.73 to 0.75 (NS, p = 0.54), (3) white milk, 0.12 to 0.11 (NS, p = 0.77), (4) flavoured milk, 0.74 to 0.85 (14.9% increase, 0.11 serves, p = 0.00)<br>b. Mean amount consumed per student of: (1) healthier foods, sable at 282g (NS, p = 1.0), (2) less healthy foods, 182.5 to 131.5 g (27.9% decrease, 51 g, p = 0.00), (3) white milk, 27.8 to 23.6 g (NS, p = 0.38), (4) flavoured milk, 190.7 to 211 g (10.6% increase, 20.2 g, p = 0.02)	Introduction of a convenience line offering healthier food items and flavoured milk prompted students to select significantly more of these items, but they did not increase consumption of healthier food items, therefore wasted more. Convenience most likely nudged the students to select more of these foods, but food preferences may have led them to limit their consumption	• Uncontrolled;<br>• Small study sample (*n* = 1 school);<br>• Menu variability on data collection days;<br>• No measurement of individual student consumption;<br>• Lack of longer-term FU.
Hanks et al. 2013 [97]	a. % student selection of: (1) fruit, (2) veg<br>b. % students consuming ≥50% of serving of: (1) fruit, (2) veg<br>c. % students consuming 100% of serving of: (1) fruit, (2) veg	a. Report cards<br>b. Researcher tray waste observations<br>c. As above<br>Data collection: total 12 days, pre-I, 2 days per school (Mar 2011), post-I, 4 days per school (May-Jun 2011); *n* = 3762 total observations (split between pre and post NR)	a. Available menu items recorded on cards; scoring of foods selected by students unclear<br>b. Tray waste data recorded on cards as (1) not eaten, (2) half-eaten, or (3) completely eaten for each student who purchased school lunch on data collection days; aggregate data (% of students) reported for pre-post comparisons<br>c. As above	a. % students who selected (1) fruit, ↑13.4% (47.3 to 53.7%; p = 0.012), and (2) veg, ↑23% (35.8 to 44.0%; $p < 0.001$)<br>b. % students consumed ≥ 50% serving of (1) fruit, ↑17.9% (40.4 to 47.7%; p = 0.004), and (2) veg, ↑24.5% (33.7 to 42.0%; $p < 0.001$);<br>c. % students consumed 100% serving of (1) fruit, ↑15.8% (31.6 to 36.6%; p = 0.006), and (2) veg, ↑9.8% (18.7 to 20.5%; p = 0.022)	Intervention effective because it guided students to take and eat more fruit and veg; Results suggest small changes in cafeterias and lunchrooms can guide students toward healthier behaviours; Larger-scale study recommended that can provide more concrete evidence for the potential efficacy of this Smarter Lunchroom intervention	• Uncontrolled;<br>• Tray waste data are repeated cross-sections;<br>• No measurement of individual student consumption;<br>• Tray waste measures do not identify what specific fruit or veg student selected and consumed.
Hunsberger et al., 2015 [123]	a. Intake of nutrients: (1) calorie, and (2) fat	a. Aggregate food weighing; data collection: B, 17 days (Jan 2010); during-I 17 days (Feb 2010)	a. Each food and beverage offered each lunch weighed before and after service to calculate gross	a. Intake of nutrients, mean amount served/student/day for: (1) calories, ↓47 kcal (668 to 621; SD of difference = 14; 95% CI −77 to −18;	Quantitative results demonstrated that calorie labels at POS ↓	• Uncontrolled;<br>• No measurement of individual student dietary intake;

Author, year of publication	Outcomes measured	Measurement tools	Tool scoring	Results	Major findings/conclusion	Limitations
	b. Student feedback on menu labelling	b. Interview; data collection: *n* = 32 audiotaped interviews (Mar-Apr 2010), 53% female	weight served; amounts entered into USDA approved nutrient database (Nutrikids) for assessment of gross calorie and fat served/day, divided by total number of students served/day = mean calorie and fat served/student/day;<br>b. *n* = 32 interviews following a guided approach (Mar-Apr 2010), audiotaped, transcribed verbatim and analysed for key themes by 3 researchers	$p = 0.004$), (2) fat, ↓2.1 g (23.1 to 21.1; SD = 0.6; 95% CI −3.3 to −0.9; $p = 0.0025$)<br>b. 5 key themes: (1) Students want nutrition information; schools have a role, (2) nutritional knowledge related to the home environment, (3) taste preference, nutrition, and healthy weight important to most students, (4) most students noticed and used calorie labels to make healthier choices, (5) calorie labels mostly not discussed among students	calories and fat served per day;<br>Qualitative results indicate labels enabled students to make healthier choices; taste, nutrition and appearance most important factors when choosing food, and students believed schools have a role to facilitate healthier eating	• Small study sample (*n* = 1 school);<br>• Lack of longer-term FU.
Just et al. 2014 [93]	a. % student selection (I-school only) of: (1) pizza dish, (2) fruit, (3) veg, (4) plain milk<br>b. % of serve consumed (I-school only) of: (1) pizza dish, (2) fruit, (3) veg, (4) plain milk<br>c. Total servings consumed of: (1) pizza dish, (2) fruit, (3) veg, (4) plain milk	a. Researcher tray waste observations; data collection: all trays in the cafeteria were included for pre-I (*n* = 2 days, 8/15 Mar 2012) vs. during-I (*n* = 1 day, 19 Apr 2012), sample size NR<br>b. Tray waste observations as above<br>c. Tray waste observation data	a. Researcher observations of whether food items offered were selected on lunch tray<br>b. Tray waste recorded using quarter waste method (0, ¼ , ½ , ¾ , or all of a specific item left on each tray) to determine % serving consumed<br>c. Total servings = % students selected multiplied by total trays observed, multiplied by average % consumed	a. % student selection: (1) pizza, ↑6.2% (91.3 to 97, $p = 0.01$), (2) fruit, ↓24.9% (25.1 to 18.9, $p = 0.013$), (3) veg, ↑57.6% (12.4 to 19.6, $p = 0.002$), (4) milk, NS<br>b. % serve consumed: (1) pizza, NS (87.8 to 90.6, $p = 0.156$), (2) fruit, NS, (3) veg: ↑16.5% (74.7 to 87.7, $p = 0.005$), (4) milk, NS<br>c. Total servings consumed: (1) pizza, ↑39.2% (137 to 191), (2) fruit, ↓0.2% (35.6 to 35.5), (3) veg, ↑133% (16 to 37), (4) milk, ↑45% (25 to 37); statistical significance NR	Connecting professional chef with NSLP is feasible; Chefs Move To Sschool program ↑ lunch sales and veg consumption in high school, suggesting potential; further work needed to generalise findings; Chef pairing of new main dish with side salad could be a by-product and long-term benefit of chef participation for menu planning;<br>Taste-testing event was an integral part of the overall experience	• Small study sample (*n* = 1 school)<br>• Chef prepared a variation of a meal that was already served and had high participation rates, limits potential I-impact<br>• Sample size for tray waste observations NR
Koch et al. 2020 [124]	a. % students selecting: (1) veg	a. Researcher observations using digital	a. A team of 9–10 researchers photographed	a. % students selecting across 3 timepoints (statistical SIG NR): (1)	Redesign of school cafeterias can have a	• Uncontrolled;

Author, year of publication	Outcomes measured	Measurement tools	Tool scoring	Results	Major findings/conclusion	Limitations
	excluding white potatoes, (2) veg white potatoes, (3) fruit, (4) grains, (5) protein, (6) milk)<br>b. Amount consumed of: (1) veg excluding white potatoes, (2) veg white potatoes, (3) fruit, (4) grains, (5) protein, (6) milk<br>c. Factors that influence school lunch consumption: (1) seated time, (2) attitudes toward school lunch, (3) school lunch participation	photography. Data collection: 2 consecutive days per timepoint, (1) pre-I, (2) post-I 3 month FU, (3) post-I 1 year FU; *n* = 899, 1193 and 1222 observations respectively<br>b. As above<br>c. (1) Digital camera time stamp, (2) Student Food Attitude survey. Data collection: timeframe NR, survey completed primarily by grade 10 students, (3) data from New York City Department of Education	lunch selections (of consenting students) at exit of lunch line; a unique ID was attached to lunch tray and items recorded<br>b. Researchers photographed lunch trays at disposal area where students brought their tray with leftover food and packaging; the amount of each item consumed was recorded in 10% increments (11-point scale)<br>c. (1) Seated time calculated as the difference between the time stamp of the before and after meal photos, (2) Survey questions across 5 domains: serving line, dining/seating, aesthetics, signage, general; responses on a 4-point Likert scale (0, 1, 2, 3; range, strongly disagree to strongly agree; higher score represents more positive attitudes), (3) New York City Department of Education data	veg excluding white potatoes, ↓ from 62% to 42% to 30%, (2) veg white potatoes, ↑ from 32% to 65% to 71%, (3) fruit, ↓ from 75% to 52% to 51%, (4) grains, relatively stable, 100% to 98%, (5) protein, relatively stable, 100% to 98%, (6) milk, stable, 38% to 38%<br>b. Amount consumed of: (1) veg excluding white potatoes (cups), from 0.15 to 0.19 ($p < 0.05$) to 0.12 (NS), (2) veg white potatoes (cups), from 0.25 to 0.47 ($p < .05$) to 0.40 ($p < 0.05$), (3) fruit (serves), from 0.48 to 0.35 ($p < 0.05$) to 0.35 ($p < 0.05$), (4) grains (serves), from 1.07 to 0.64 ($p < 0.05$) to 0.62 ($p < 0.05$), (5) protein (serves), from 0.75 to 0.82 ($p < 0.05$) to 0.82 ($p < 0.05$), (6) milk (serves), stable, 0.29 (NS)<br>c. Across 3 timepoints: (1) seated time ↑ from 13:25 minutes to 15:22 ($p = 0.041$), (2) all attitude scales ↑ but remained between 1–2; serving line ($p = 0.002$), dining seating ($p = 0.001$), aesthetics ($p = 0.011$), signage ($p = 0.015$), general ($p < 0.001$); perception of noise ($p = 0.035$), (3) school lunch participation ↑ from 21% to 36% to 41% ($p = 0.004$)	positive impact on students' participation, time spent in the cafeteria, attitudes towards the lunch meal, and their dining experience. There were no positive changes to food consumed. Authors suggest the daily presence and promotion of French fries led to a decrease in consumption of other vegetables, highlighting the importance of a health promoting menu alongside cafeteria re-designs.	• Schools not randomly selected;<br>• Attitudes survey primarily completed by grade 10 students rather than across all grades (i.e., 9 to 12);<br>• Universal Free Meals to all students was initiated during the intervention, potentially confounding participation results.

Author, year of publication	Outcomes measured	Measurement tools	Tool scoring	Results	Major findings/conclusion	Limitations
Madden et al. 2013 [105]	a. Amount consumed of: (1) fruit, (2) veg<br>b. Intake of nutrients: (1) energy, (2) protein, (3) total fat, (4) saturated fat, (5) carbohydrate, (6) zinc, (7) calcium, (8) vitamin C, (9) vitamin A, (10) iron, (11) folate	a. Food data collection (FDC) sheets used to record visual observation of food items served and weighing of food portions uneaten; data collection: pre-I $n = 5$ days vs. post-I $n = 5$ days<br>b. Nutrient analysis software (FOODBASE v3.1)	a. Fruit and veg data collapsed into 5 categories for assessment: (1) fruit, all types, (2) veg, all cooked including pasta sauce, (3) salad, (4) tomato puree, (5) ketchup; quantity consumed/student/ observation = food served using standard portions less waste remaining (both weighed);<br>b. Data from FDC sheets entered into FOODBASE to calculate nutrient intake from food consumed	a. Mean consumption: overall fruit and veg ↑ 12.0–30.3 g ($p < 0.001$); fruit ↑ 0–7.9 g ($p < 0.001$), veg ↑ 1.8–2.7 g (NS), salad ↑ 1.5–11 g ($p < 0.001$), tomato puree + ketchup 8.7–8.8 g (NS)<br>b. Mean nutrient intake: energy, protein, total fat, saturated fat ↓ (all $p < 0.01$), carbohydrate and zinc ↓ (both $p<0.05$), calcium ↓, vitamin C ↑ and vitamin A ↓ (all $p < 0.005$), NS difference for iron and folate	Total and saturated fat, and fruit and veg intake can be significantly improved by a short, kitchen-based intervention. While mean fruit and veg intake per school lunch ↑ to 30 g, this falls well below recommended provision of approx. 160 g per school lunch. Further action is required to optimise intake from school lunches	• Small study sample ($n = 1$ school)<br>• Uncontrolled<br>• Non-random selection of participating students<br>• Opportunistic sampling children in dining hall not reflecting intake of all children
McCool et al. 2005 [108]	a. Amount consumed of whole apples vs. sliced apples	a. Researcher observations: aggregate food weighing. Data collection: 2 days per week over 12 weeks; three timepoints, (1) 6 weeks, whole apple, (2) 4 weeks, sliced apple, (3) 2 weeks, choice between whole or sliced apple	a. Total fruit weighed prior to lunch period; un-issued fruit and waste (monitored by study personnel) were weighed after lunch to calculate aggregate amount consumed by students per day	a. Mean amount consumed (pounds) per day, students consumed more when given a choice, and more sliced than whole apples: timepoint 1, whole apples, 73.8 (SD 16.9); timepoint 2, sliced apples, 76.3 (SD 11.9); timepoint 3, choice, all apples 86.7 (whole 28.3, SD 4.1; sliced 58.4, SD 1.7)	Middle school students consumed more apples when they were offered both whole or sliced apples; students preferred sliced to whole apples	• Inadequate statistical analyses
Pope et al. 2018 [94]	a. % students selecting: (1) all NSLP entrée, (2) new entrée, and (3) alternative entrée<br>b. NSLP participation (% students) for (1) full price students, and (2) FRP eligible students<br>c. Lunch revenue generated by the	a. School data lunch sales; data collection: $n = 4$ days/month when new entrees offered; measurements at B (Sep 2015), during-I (Oct 2015), post-I FU (Nov 2015)<br>b. School data NSLP participation<br>c. Cafeteria sales data	a. Mean daily sales of (1) total NSLP lunches, (2) new entrees, and (3) alternative entrees<br>b. % students eligible for FRP or full-price meals participating in the NSLP on days targeted entrees were served<br>c. Revenue generated for each day targeted entrée was served; lunch revenue calculated using	a. % students selecting: (1) all NSLP entrée ↑ (57 to 62% B to FU, NS); (2) new entrée ↑ (31 to 40% B to FU; $p < 0.001$); (3) alternative entrée ↓ (27 to 21% B to FU; $p < 0.001$)<br>b. NSLP participation among FRP eligible students ↑ (82 to 92%; $p < 0.001$), no change among full-price students<br>c. Net revenue ↑ for 3 of the 4 new menu items (total +$292.53 B to FU).	There was a significant ↑ in the % students who chose targeted entrée at FU but not during-I;<br>Sampling may have a positive effect on NSLP participation rates, especially for those eligible for FRP meals	• Participation rate engaging in taste testing NR; limits the ability to conclude changes resulted from taste-testing<br>• Small study sample ($n = 1$ school)<br>• Uncontrolled

Author, year of publication	Outcomes measured	Measurement tools	Tool scoring	Results	Major findings/conclusion	Limitations
	foodservice program		federal reimbursement rate: \$3.13 per FRP or \$0.35 per full-price lunch			
Prell et al. 2005 [101]	a. Fish consumption (% students) for (1) eaters, (2) tasters, and (3) non-eaters<br>b. Knowledge about fish	a. Observations; data collection: $n = 5$ days over 5 weeks; comparison of cumulative relative frequencies, B vs. FU<br>b. 10-item questionnaire; data collection: B vs. FU	a. Fish selection and plate waste when fish was served, visually compared to sample portions; students categorised as (1) eaters: consumed >50% of fish served on ≥3 of 5 days, (2) tasters: consumed ≤50% on ≥1 of 5 days, or (3) non-eaters: students on 3 of 5 days were ill, did not appear at the canteen or if data missing on fish served or leftover<br>b. 7-point Likert scale (definitely no to definitely yes)	a. Eaters: SL-group ↑ from 59-69%, SLHE-group ↑ 56-71%, C-group ↓ 77-69%.<br>Systematic disagreement in position measured as RP between B and FU: C-group (RP −0.08; 95% CI −0.17 to 0.01), SL-group (RP 0.10; 95% CI −0.02 to 0.22, NS) and SLHE-group (RP 0.15; 95% CI 0.06 to 0.24, SIG)<br>b. Knowledge; total correct responses (mean), B vs. FU: SL-group ↑ from 2.8-4.1 ($p < 0.001$), SLHE-group ↑ 3.8-4.8 ($p < 0.001$), C-school ↑ from 3.2-3.4 (NS)	SIG ↑'s in students' knowledge about fish (compared with C-school) were detected in both I-groups. SLHE-group SIG influenced dietary behaviour (fish consumption), while suggestive improvements observed in SL-group did not result in any SIG differences to fish consumption.	• Baseline disparity between groups;<br>• High drop-out rates assoc. with measurement points criteria, high attrition bias/incomplete data;<br>• Short-term intervention ($n = 5$ weeks)
Prescott et al. 2019 [99]	a. % student selection of: (1) veg (hot veg + salad bar veg), (2) fruit (whole fruit + salad bar fruit), (3) entrée, and (3) milk<br>b. Amount food waste (g) for (1) hot veg, (2) salad bar veg, (3) whole fruit, (4) salad bar fruit, (5) entrée, and (6) milk<br>c. % of serve consumed of: (1) veg (hot veg + salad bar veg), (2) fruit (whole fruit + salad bar fruit), (3) entrée, and (4) milk	a. Researcher observation at cashier<br>b. Digital photography method (validated); 3 timepoints for analyses, 1 day/month, total tray observations, $n = 778$ (split between I and C not reported: pre-I, $n = 256$ (Nov 2017); post-I, $n = 236$ (Dec 2017); post-I 5-month FU, $n = 286$ (Apr 2018)<br>c. As above	a. Researchers recorded gender, grade, and selected food items for each student on pre-printed tray tags with day's menu options; % students who selected items from each food group: recorded as binary (selected/not select)<br>b. Photographs visually assessed for % waste of average reference food (g) calculated from 3–5 reference food samples at each time point<br>c. % of each food group consumed/student	a. Between groups: veg selection at B, post-I and FU, C > I, 35.9% vs. 22.5% ($p = 0.021$), 53% vs. 51.8% (NS) and 35.3% vs. 23.2% (NS) respectively. Fruit, entrée and milk all NS difference in selection at B, post-I, FU.<br>Within groups, I-group from B to post-I to FU: (1) veg, 22.5% to 51.8% (↑ 29.3%) to 23.2%, (2) fruit, 88.2% to 87.1% to 91.9%, (3) entrée, 99.1% to 100%, (4) milk, 81.1% to 80% to 75.7%; statistical SIG NR<br>b. Between groups: hot veg waste at B, I > C, 26.4 g vs. 6.1 g ($p = 0.015$), NS difference at post-I and FU; salad bar veg: at B and post-I NS, at FU C > I, 50.1 g vs. 24.2 g ($p = 0.029$); whole fruit: NS at B, post-I	Adolescents who received a food systems education and promotion intervention increased their vegetable and fruit consumption relative to baseline	• Short-term intervention<br>• Baseline disparity between groups (veg selection and consumption)<br>• Age differences between groups due to different grade levels (C-group 1–2 years older than I-group)

Author, year of publication	Outcomes measured	Measurement tools	Tool scoring	Results	Major findings/conclusion	Limitations
				and FU; salad bar fruit: at B and post-I NS, at FU waste C > I, 70.8 g vs. 46.1 g (*p* = 0.036); entrée and milk: NS at B, post-I and FU<br>c. Between groups: veg consumption at B, C > I, 71.8% vs. 47.1% (*p* = 0.006), NS at post-I and FU. Fruit consumption at B, C > I, 57.9% vs. 44.0% (*p* = 0.009), NS at post-I and FU. Entrée and milk consumption, NS at post-I and FU.<br>Within groups, I-group from B to post-I to FU: (1) veg, 47.1% to 69.4% to 63.82%, (2) fruit, 44% to 51.1% to 52.5%, (3) entrée, 79.1% to 87.7% to 83.1%, (4) milk, 64.9% to 61% to 61%; statistical SIG NR		

Author, year of publication	Outcomes measured	Measurement tools	Tool scoring	Results	Major findings/conclusion	Limitations
Quinn et al. 2018 [98]	a. Number of serves selected of: (1) fruit including juice, (2) fruit excluding juice, (3) veg including potatoes, (4) veg excluding potatoes, (5) low-fat white milk<br>b. % students selecting food items as above<br>c. Number of serves consumed of food items as above<br>d. % students consuming ≥25% of food items as above	a. Observation<br>b. Observation<br>c. Observation and plate waste<br>d. Observation and plate waste<br>Data collection: *n* = 4 days (2 days/school); total tray observations, *n* = 2309 (I and C-schools, *n* = 1150 and 1159 respectively); pre-I, 1 day/school, *n* = 902 (I and C-schools, *n* = 416 and 486 respectively, Sep-Oct 2013); post-I, 1 day/school, *n* = 1407 (I and C-schools, *n* = 734 and 673 respectively, May 2014)	a. Lunchroom tables numbered and study sample selected using a random number generator; researchers recorded type and quantity of items selected by each student on data collection card and taped card to student's trays<br>b. % students selecting food items calculated using data collection cards and total student sample from selected tables<br>c. Students returned trays to labelled rack once finished; using displayed reference portions, data collectors estimated leftover portion using quarter-waste method (0, ¼ , ½ , ¾ or all remaining) to determine number of serves consumed<br>d. % students who consumed any, defined as ≥25% fruit, veg or milk using plate waste data as above	a. Mean number of items selected ↑ for I vs. C for fruit including juice ($p = 0.001$) and fruit excluding juice ($p < 0.001$); NS difference for veg or milk<br>b. % students selecting items ↑ for I vs. C (adjusted analyses) for fruit including juice ($p = 0.004$), fruit excluding juice ($p < 0.001$); NS difference for veg or milk<br>c. Number of items consumed (of those who selected) ↓ in all categories for I and C (adjusted analyses); SIG greater change in C vs. I for fruit excluding juice ($p = 0.03$) and veg including potatoes ($p = 0.02$); NS difference for fruit including juice, veg excluding potatoes, milk<br>d. % students who consumed any (≥25%) fruit excluding juice ↑ for I vs. C (unadjusted; $p = 0.04$); NS consumption for other categories	Results indicate choice architecture can promote student selection of healthy foods, but have limited effects on their consumption	• Schools not randomly assigned to groups<br>• Baseline disparities between I and C-schools FRP meals eligibility (by design, I-schools had > proportions of students eligible for FRP meals);<br>• Varied lunch menus across data collection days and seasonal variation (or temporal variation) a possible influence on findings<br>• Single day of data collection pre and post creates potential for bias.
Schwartz et al. 2015 [92]	a. % students selecting: (1) fruit, (2) veg, (3) entrée, (4) low-fat white milk<br>b. % of serve consumed of food items as above<br>c. % students selecting fruit according to	Researcher observations: photography and weighing of food items served and uneaten<br>Data collection: 3 timepoints; pre-I (Apr 2012, *n* = 502) and post-I (May 2013, *n* = 465 and Jun 2014, *n* = 373), *n* = 36 days (1 day/year/school)	a. Mean % of students selecting each meal component; at cashier, researchers numbered and photographed lunch trays<br>b. Mean % consumed of each meal component; at conclusion of meal researchers collected all	a. Mean % of students selecting: fruit ↑ (54 to 66%; $p < 0.05$), veg NS ↓ (68 to 52%), entrée ↑ (91 to 98%; $p < 0.05$), milk stable<br>b. Mean % of each meal consumed: fruit stable (72 to 74%; NS), veg ↑ (46 to 64%; $p < 0.05$), entrée ↑ (71 to 84%; $p < 0.05$), milk stable (54 to 57%; NS)	The revised meal standards and policies appeared to lower plate waste. Students consumed more fruit, wasted less entrée and veg, and consumed same amount of milk. More students consumed fruit.	• Cross-sectional design limits reliability of observed changes and does not allow for analysis of individuals change in food behaviours<br>• Uncontrolled

Author, year of publication	Outcomes measured	Measurement tools	Tool scoring	Results	Major findings/conclusion	Limitations
	number of fruit options offered		lunch trays, weighed and recorded uneaten components, classified as either entrée, fruit, veg or milk, and compared to pre-lunch reference weight (average of 3 servings/lunch period); photographs provided evidence for items completely consumed<br>c. As per (a) above	c. Increasing the number of fruit options by 1 is associated with a SIG ↑ of 9.3% in fruit servings selected by students		• Data collected once per year<br>• Declining sample selecting featured lunch: n = 502 in pre period to n = 373 post
Sharma et al. 2018 [106]	a. Service speed of fast service lane (FSL) vs. regular service lanes (RSLs)<br>b. Student satisfaction of service speed and meal quality	a. Time measurements<br>b. Exit survey instrument<br>Data collection: n = 18 days, data collected randomly from students that visited FSL and 2 RSLs; procedures tested in a pilot study	a. Researchers used stopwatches to record the time taken by every 5th student (to allow for accurate time recording and reset for next student) to go from entry to exit in FSL and RSLs<br>b. Satisfaction rated on a 5-point Likert scale (1 = very dissatisfied to 5 = very satisfied); 2 questions, (1) I am satisfied with the quality of the meal, and (2) I am satisfied with the speed of the meal	a. Between groups: mean student service time faster in FSL (n = 387 students) compared to RSLs (n = 335 students), 39.4 secs vs. 143.7 secs respectively (t-stat = −32.1, p < 0.01)<br>b. Between groups: n = 272 student survey responses (grades 6–12); service speed satisfaction ratings higher for FSL (for grades 6, 7, 10 and 11; p < 0.01); NS difference in meal quality satisfaction ratings	Students were satisfied with service speed but there was no difference in meal quality satisfaction between 2 service options. Lower grades' scoring of quality and service speed > than higher grades; authors suggest higher grades begin to prefer > choices and therefore > loss aversion from lack of choices than lower grade students	• Short-term intervention (n = 4 weeks)<br>• Small study sample (n = 1 school)<br>• Aggregate data collected without matching individual data for participation in FSL and exit survey
Turnin et al. 2016 [112]	a. % students selecting: (1) dairy products (yoghurt or fromage blanc), (2) cheese, (3) starch, (4) fruits and veg, (5) pastry, ice cream, dessert<br>b. BMI z-score and obesity prevalence	a. Data set from Nutri-Advice software<br>Data collection: B, first 3 kiosk uses (Dec) vs FU, last 3 uses (Apr)<br>b. Anthropometric measurements taken at B (Nov) vs. FU (May)	a. Data set downloaded for each connection to kiosk; foods selected for lunch coded to 5 groups, (1) dairy, (2) cheese, (3) fruit and veg, (4) starch, (5) desserts. Per student, results expressed as overall % of each food group selected	a. Students' food choice competency of dairy, fruit and veg, starch ↑ (p = 0.03, 0.05, 0.03 respectively); cheese, desserts ↓ (p = 0.002 and < 0.001 respectively); school C showed changes consistent with healthy food policies for all food groups; school A showed NS differences; school B showed some SIG changes (starch and cheese)	Personalised nutrition counselling through an interactive device has the potential to improve the food choice competencies of children	• Kiosk location was at the discretion of schools<br>• Baseline disparity between groups (obesity prevalence)<br>• Comparison of first 3 and last 3 uses of the kiosk limits reliability of observed changes

Author, year of publication	Outcomes measured	Measurement tools	Tool scoring	Results	Major findings/conclusion	Limitations
			b. BMI z-score calculated and obesity defined as gender and age specific BMI above 97th percentile according to French reference curves (validated)	b. Overall BMI z-score ↓ (0.50 to 0.43; $p < 0.001$) and obesity prevalence ↓ (12.3% to 10.3%; $p = 0.04$); BMI z-score SIG in 2 of 3 schools; obesity prevalence SIG in 1 of 3 schools		• Uncontrolled
Wansink et al. 2015 [95]	a. % student selection of: salad<br>b. % of serve consumed of: salad	a. Researcher observation<br>b. Tray waste assessment<br>Data collection: $n = 554$ tray waste observations across 3 days; 2 days pre-I (29 Feb, $n = 179$ students; 13 Mar, $n = 194$ students) and 1 day post-I (24 Apr, $n = 181$ students)	a. Daily binary count variables from tray waste records (1 for salad serving; 0 otherwise)<br>b. Reference salad serve size (from pre-lunch visual inspection) less estimated tray waste using quarter-waste method (validated; 0, ¼ , ½ , ¾ or all remaining)	a. % students selecting salad ↑ from 2% to 10% ($p < 0.001$)<br>b. % salad consumed ↓ from 94% to 67% ($p = 0.007$)	Pilot suggests that school gardens can ↑ selection by 8 percentage points (2% to 10%), but 1/3 gets wasted. More students took salad when the greens were from the school garden	• Short-term intervention ($n = 1$ day); findings may indicate a novelty effect<br>• Small study sample ($n = 1$ school)<br>• Uncontrolled
Wansink et al. 2013 [111]	a. % student selection of: apples<br>b. % of serve consumed of: apples<br>c. % of serve wasted of apples	a. Tray waste record<br>b. Tray waste record<br>c. Researcher observation and tray waste assessment<br>Data collection: $n = 643$ tray waste observations across all timepoints (split between I and C NR); I-schools, $n = 4$ days (2 days pre-I; 2 days during-I); C-schools, $n = 2$ days (both during-I)	a. Number of students who selected an apple (proxy for daily apple sales) counted using tray waste records; binary outcome (apple taken or apple not taken)<br>b. % apple serving consumed/student from tray waste record<br>c. % apple serving wasted/student from paper-based tray waste record of whether o, ½ , ¾ , or all of apple remaining	a. Daily apple sales ↑ by 71% in I-schools compared to C-schools ($p < 0.01$)<br>b. Students that consumed >50% of apple ↑ by 73% in I-schools ($p = 0.02$)<br>c. Students that wasted >50% of apple ↓ by 48% in I-schools ($p = 0.03$)	Study results suggest sliced fruit is more appealing to children than whole fruit because it is easier and tidier to eat;<br>Fruit slicers can serve as an effective, low-cost measure to ↑ fruit consumption and ↓ food waste	• Fruit slicers may indicate a novelty effect<br>• C-school data only collected during I-period
Witschi et al. 1985 [125]	a. Intake of nutrients: sodium<br>b. Food acceptability	a. 24-hour food diaries. Data collection: pre-I, $n = 4$ days randomly assigned 1 day/week over 4 weeks, $n = 309$ student records; during-I, $n = 5$ days over 5	a. Food diaries and recipe analysis using Quick Input of Food program<br>b. (1) comparison of average food intake (caloric consumption), (2) all students leaving the	a. Mean daily sodium intake ↓ 35.7% (136.4 ± 3.9 mEq to 87.7 ± 2.2 mEq; $p < 0.0001$)<br>b. (1) Average caloric intake pre-I vs. during-I, males: 2926 ± 101 to 2769 ± 95 (NS, $p = 0.3$); females: 1974 ± 50 to 1940 ± 48 (NS, $p = 0.6$), (2)	The amount of sodium in prepared foods can be ↓ by 50% without loss of taste and acceptability;<br>The large amount of experimentation by the	• Self-reported student data (food diary)<br>• Small study sample ($n = 1$ school)<br>• Uncontrolled

Author, year of publication	Outcomes measured	Measurement tools	Tool scoring	Results	Major findings/conclusion	Limitations
		weeks, n = 318 student records<br>b. Two methods to assess acceptability: (1) 24-hour food diaries to measure caloric consumption (per data collection above), and (2) palatability survey	dining hall on selected study days (number of days not reported) were asked to judge the taste acceptability of specific foods (modified and unmodified) that had been served during meal; scored using a finite scale (range a = much better to e = much worse); students blinded to whether the food was made by the usual or modified recipe	student ratings of pre-I and during-I almost identical: pre-I, n = 1036 respondents, average rating = 2.75; during-I, n = 748 respondents, average rating = 2.74	food service department to develop palatable products undoubtably played an important role in their acceptance	

↑: increase; ↓: decrease; approx.: approximately; B: baseline; BE: behavioural economics; BMI: body mass index; BP: blood pressure; C: control or comparison; CBPR: community-based participatory research; CI: confidence interval; DID: difference-in-difference estimate; FU: follow-up; I: intervention; FRP: free or reduced-price; NR: not reported; NS: not significant; NSLP: National School Lunch Program; NYC: New York City; POS: point of selection; RP: relative position; sat fat: saturated fat; SD: standard deviation; SE: standard error; SFA: saturated fatty acids; SIG: significant; SLHE: school lunch plus home economics intervention; SL: school lunch intervention; SNaX: Students for Nutrition and eXercise intervention; SSB: sugar sweetened beverages; USDA: United States Department of Agriculture; Veg: vegetables.

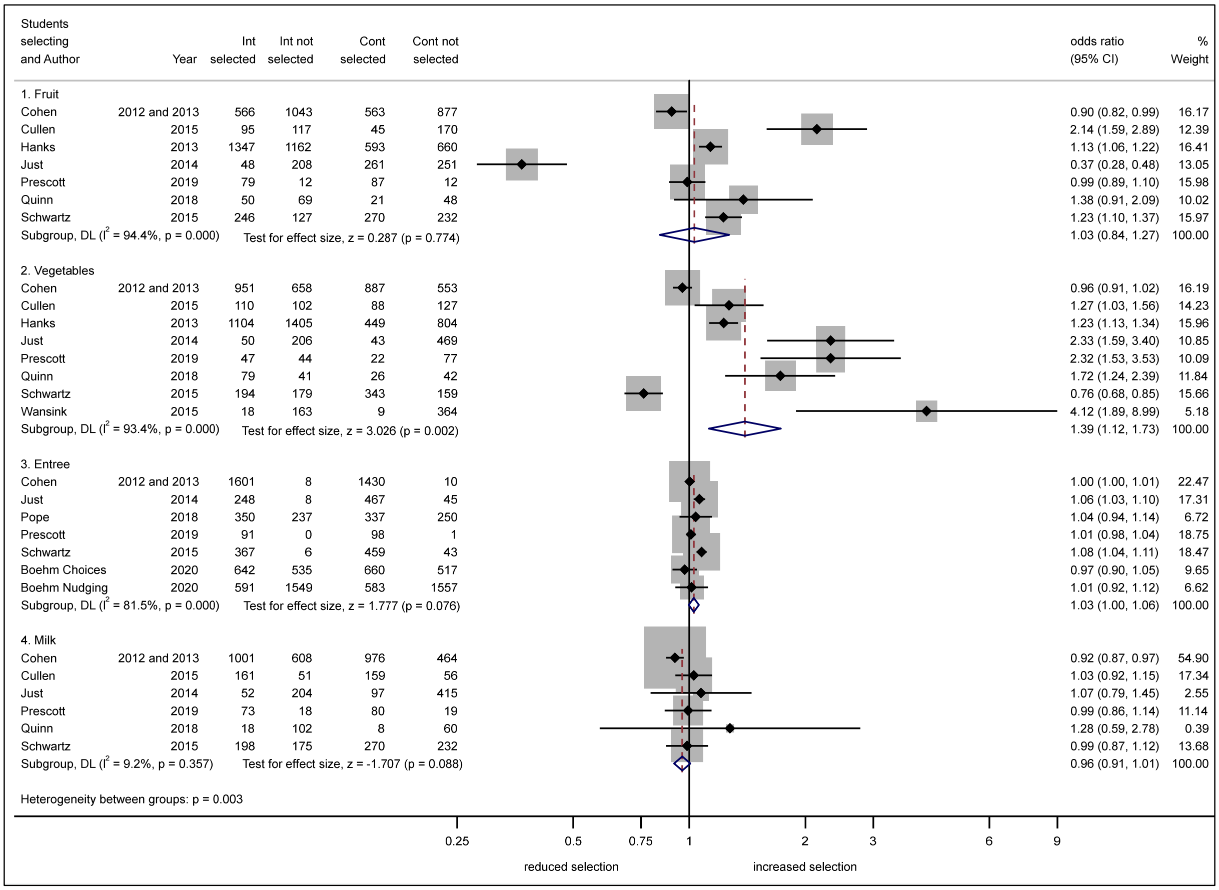

Figure S1. Forest plot of the effect of interventions on proportion of students selecting meal component: 1. fruit, 2. vegetables, 3. entrée and 4. Milk [89,90,92–99,119]. Weights are from random-effects model. Low risk of bias status: Cohen et al. (2012) [89]; Cullen et al. (2015) [90]. CI: confidence interval; Cont: control; DL: DerSimonian-Laird estimate; Int: intervention.

(a)

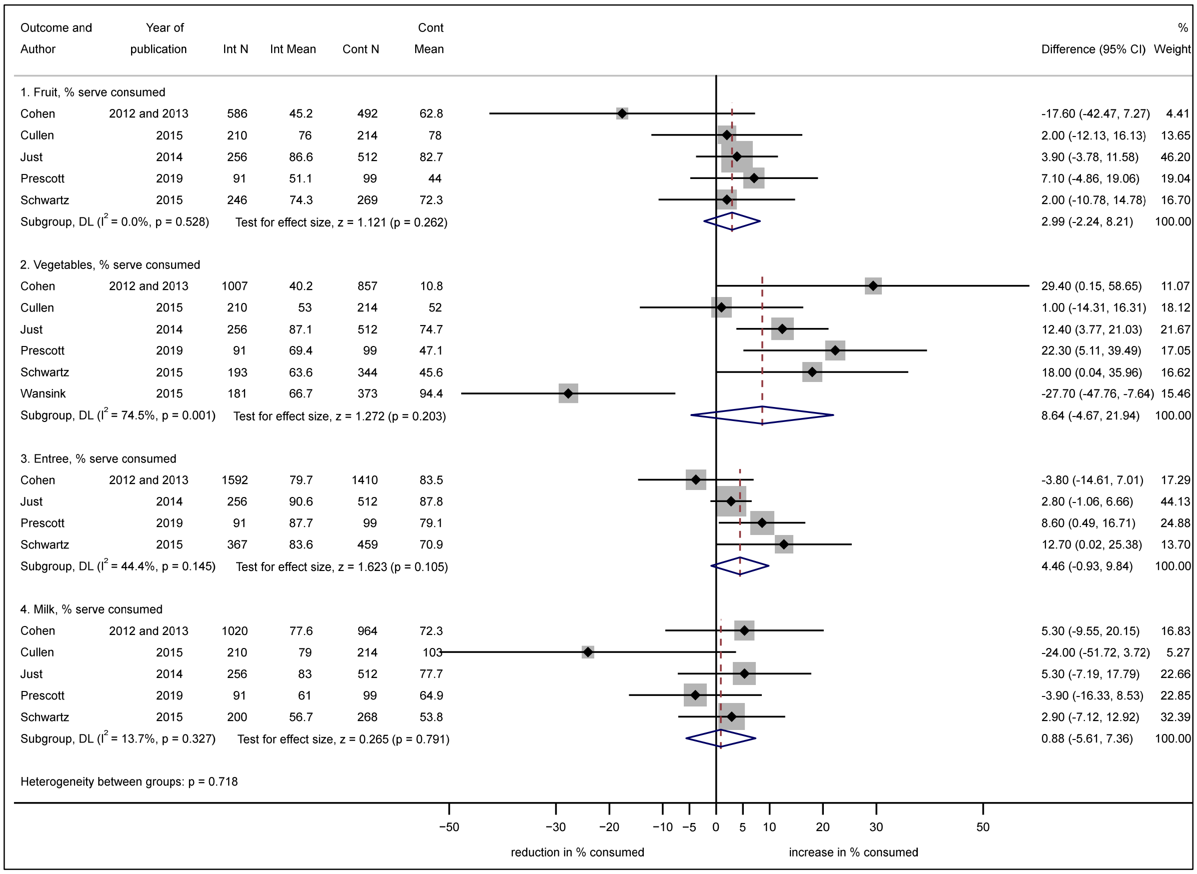

Outcome and Author | Year of publication | Int N | Int Mean | Cont N | Cont Mean | Difference (95% CI) | % Weight
1. Fruit, % serve consumed
Cohen 2012 and 2013 586 45.2 492 62.8 -17.60 (-42.47, 7.27) 4.41
Cullen 2015 210 76 214 78 2.00 (-12.13, 16.13) 13.65
Just 2014 256 86.6 512 82.7 3.90 (-3.78, 11.58) 46.20
Prescott 2019 91 51.1 99 44 7.10 (-4.86, 19.06) 19.04
Schwartz 2015 246 74.3 269 72.3 2.00 (-10.78, 14.78) 16.70
Subgroup, DL (I^2 = 0.0%, p = 0.528) Test for effect size, z = 1.121 (p = 0.262) 2.99 (-2.24, 8.21) 100.00
2. Vegetables, % serve consumed
Cohen 2012 and 2013 1007 40.2 857 10.8 29.40 (0.15, 58.65) 11.07
Cullen 2015 210 53 214 52 1.00 (-14.31, 16.31) 18.12
Just 2014 256 87.1 512 74.7 12.40 (3.77, 21.03) 21.67
Prescott 2019 91 69.4 99 47.1 22.30 (5.11, 39.49) 17.05
Schwartz 2015 193 63.6 344 45.6 18.00 (0.04, 35.96) 16.62
Wansink 2015 181 66.7 373 94.4 -27.70 (-47.76, -7.64) 15.46
Subgroup, DL (I^2 = 74.5%, p = 0.001) Test for effect size, z = 1.272 (p = 0.203) 8.64 (-4.67, 21.94) 100.00
3. Entree, % serve consumed
Cohen 2012 and 2013 1592 79.7 1410 83.5 -3.80 (-14.61, 7.01) 17.29
Just 2014 256 90.6 512 87.8 2.80 (-1.06, 6.66) 44.13
Prescott 2019 91 87.7 99 79.1 8.60 (0.49, 16.71) 24.88
Schwartz 2015 367 83.6 459 70.9 12.70 (0.02, 25.38) 13.70
Subgroup, DL (I^2 = 44.4%, p = 0.145) Test for effect size, z = 1.623 (p = 0.105) 4.46 (-0.93, 9.84) 100.00
4. Milk, % serve consumed
Cohen 2012 and 2013 1020 77.6 964 72.3 5.30 (-9.55, 20.15) 16.83
Cullen 2015 210 79 214 103 -24.00 (-51.72, 3.72) 5.27
Just 2014 256 83 512 77.7 5.30 (-7.19, 17.79) 22.66
Prescott 2019 91 61 99 64.9 -3.90 (-16.33, 8.53) 22.85
Schwartz 2015 200 56.7 268 53.8 2.90 (-7.12, 12.92) 32.39
Subgroup, DL (I^2 = 13.7%, p = 0.327) Test for effect size, z = 0.265 (p = 0.791) 0.88 (-5.61, 7.36) 100.00
Heterogeneity between groups: p = 0.718
−50 −30 −20 −10 −5 0 5 10 20 30 50
reduction in % consumed
increase in % consumed

(b)

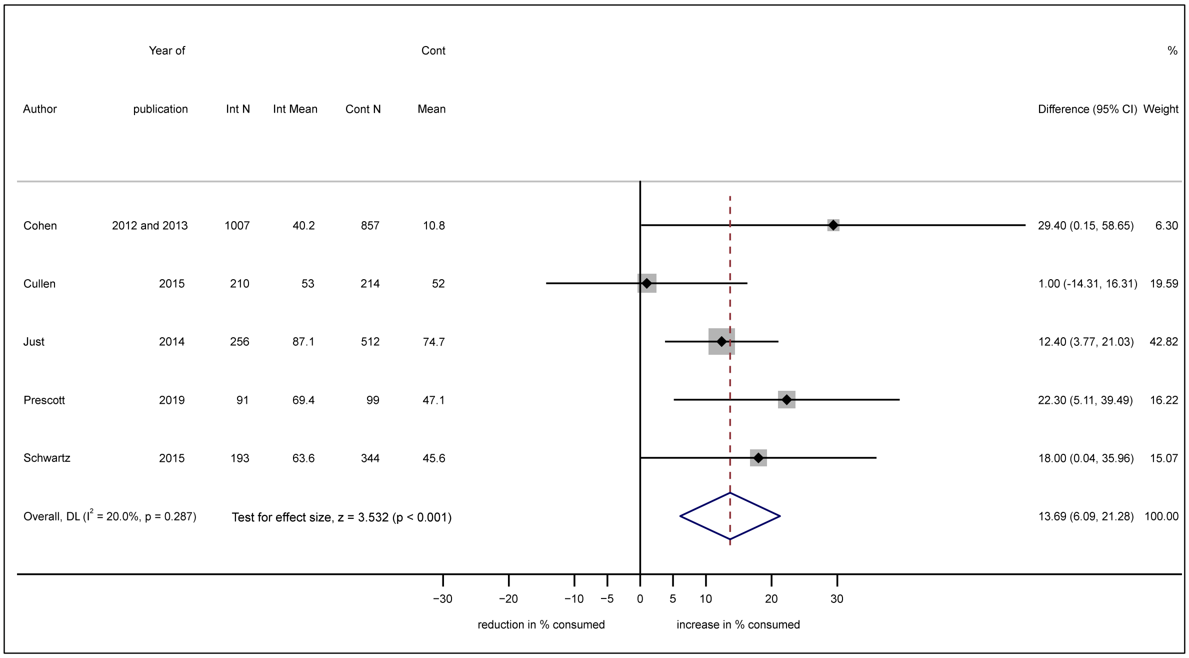

Figure S2. (**a**) Forest plot of the effect of interventions on the percent of serve consumed by students who selected a meal component: 1. fruit, 2. vegetables, 3. entrée and 4. Milk [89,90,92,93,95,99,119]. Weights are from random-effects model. Low risk of bias status: Cohen et al. (2012) [89]; Cullen et al. (2015) [90]. (**b**) Forest plot of the effect of interventions on the percent of serve consumed by students who selected vegetables (sensitivity analysis excluding Wansink et al. (2015) [95]. Weights are from random-effects model. Low risk of bias status: Cohen et al. (2012) [89]; Cullen et al (2015) [90]. CI: confidence interval; Cont: control; DL: DerSimonian-Laird estimate; Int: intervention; N: sample size.

(a)

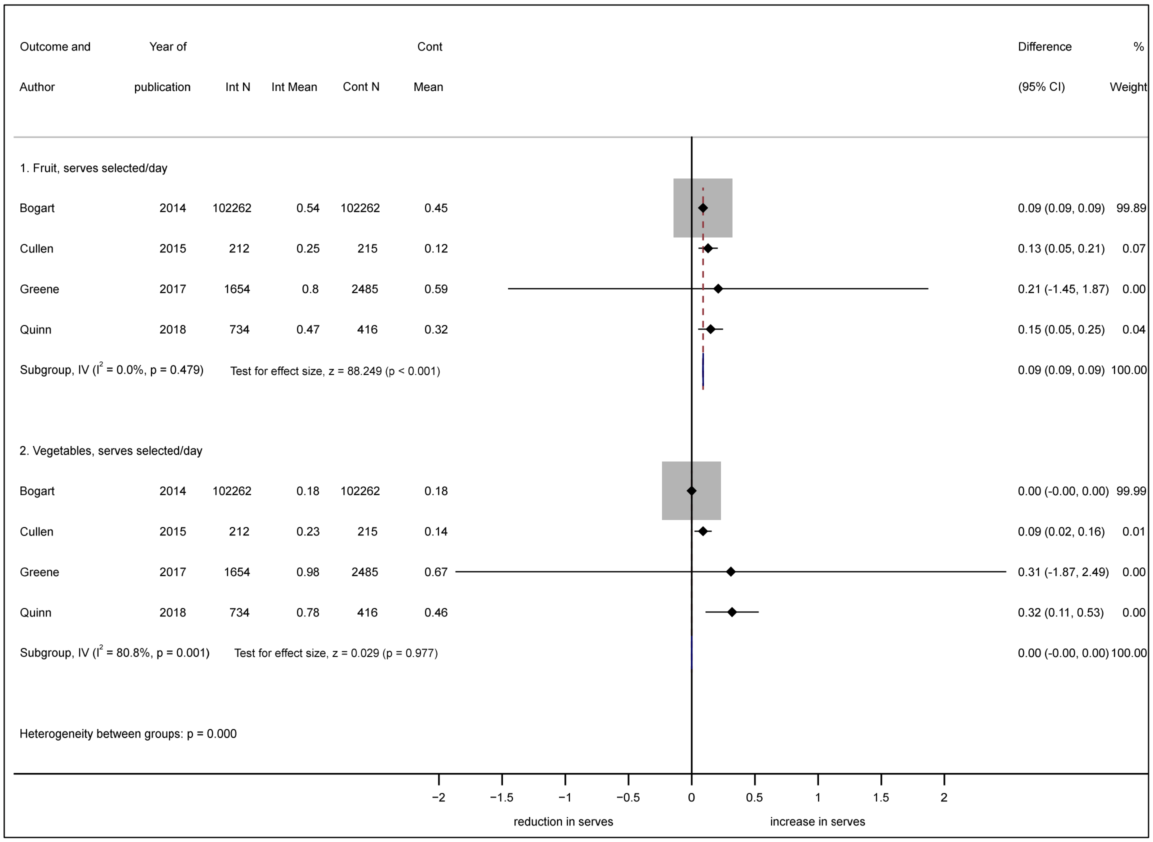

Outcome and Author
Year of publication
Int N
Int Mean
Cont N
Cont Mean
Difference (95% CI)
% Weight
1. Fruit, serves selected/day
Bogart 2014 102262 0.54 102262 0.45 0.09 (0.09, 0.09) 99.89
Cullen 2015 212 0.25 215 0.12 0.13 (0.05, 0.21) 0.07
Greene 2017 1654 0.8 2485 0.59 0.21 (-1.45, 1.87) 0.00
Quinn 2018 734 0.47 416 0.32 0.15 (0.05, 0.25) 0.04
Subgroup, IV (I^2 = 0.0%, p = 0.479) Test for effect size, z = 88.249 (p < 0.001) 0.09 (0.09, 0.09) 100.00
2. Vegetables, serves selected/day
Bogart 2014 102262 0.18 102262 0.18 0.00 (-0.00, 0.00) 99.99
Cullen 2015 212 0.23 215 0.14 0.09 (0.02, 0.16) 0.01
Greene 2017 1654 0.98 2485 0.67 0.31 (-1.87, 2.49) 0.00
Quinn 2018 734 0.78 416 0.46 0.32 (0.11, 0.53) 0.00
Subgroup, IV (I^2 = 80.8%, p = 0.001) Test for effect size, z = 0.029 (p = 0.977) 0.00 (-0.00, 0.00) 100.00
Heterogeneity between groups: p = 0.000
−2 −1.5 −1 −0.5 0 0.5 1 1.5 2
reduction in serves
increase in serves

(b)

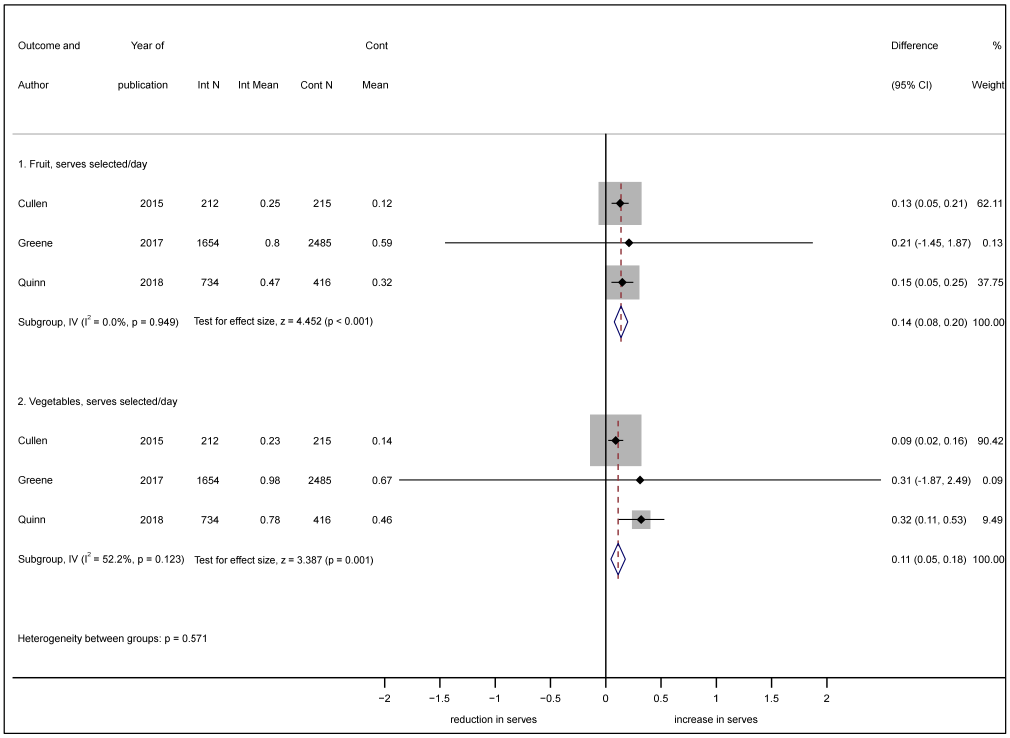

Figure S3. (**a**) Forest plot of the effect of interventions on the mean number of serves selected by students per day by meal component: 1. fruit and 2. Vegetables [88,90,91,98]. Weights are from fixed-effects model. Low risk of bias status: Bogart et al. (2014) [88]; Cullen et al. (2015) [90]; Greene et al. (2017) [91]. (**b**) Effect of interventions on the mean number of serves selected by students per day by meal component: 1. fruit and 2. vegetables (sensitivity analysis excluding Bogart et al. (2014) [88]. Weights are from fixed-effects model. Low risk of bias status: Cullen 2015 [90], Greene 2017 [91]. CI: confidence interval; Cont: control; Int: intervention; N: sample size.

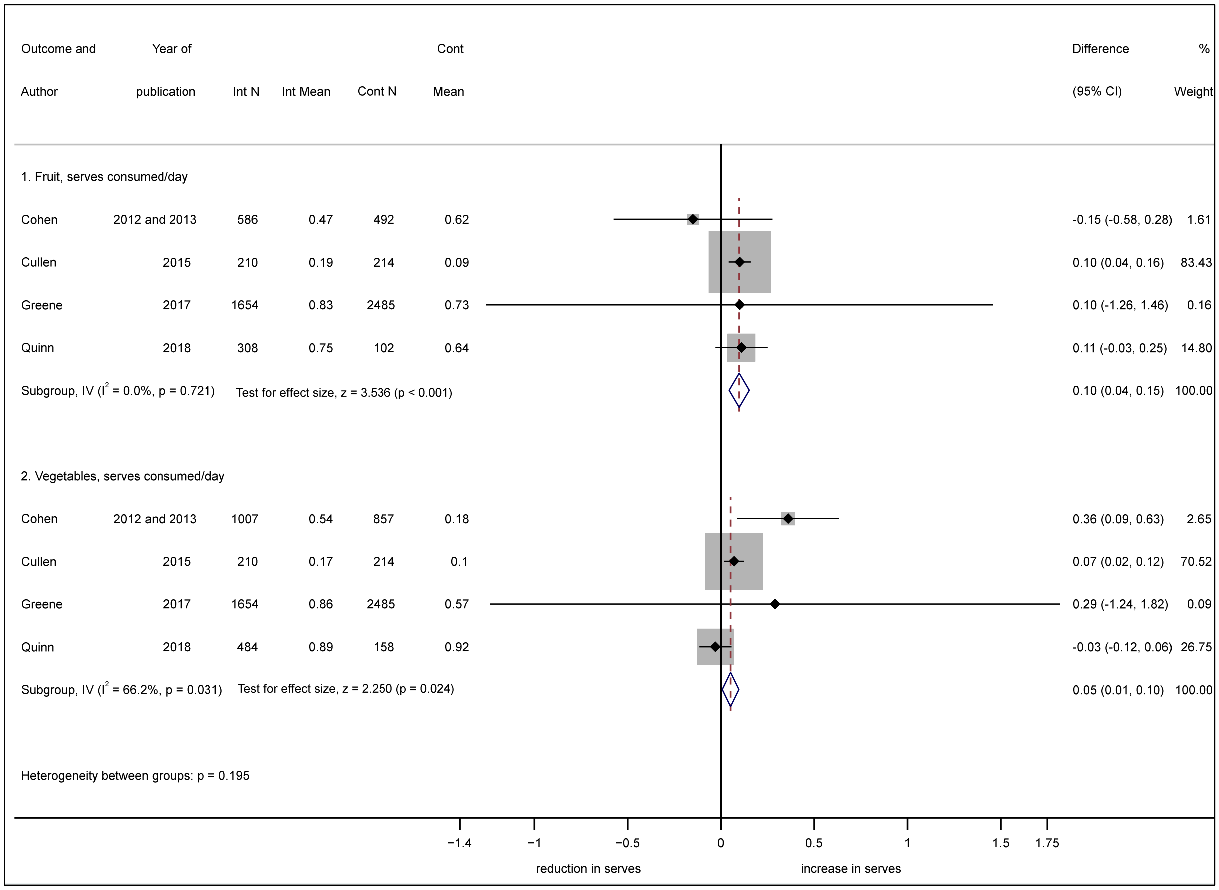

Figure S4. Forest plot of effect of interventions on the mean number of serves consumed by students per day by meal component: 1. fruit and 2. Vegetables [89–91,98,119]. Weights are from fixed-effects model. Low risk of bias status: Cohen et al. (2012) [89]; Cullen et al. (2015) [90]; Greene et al. (2017) [91]. CI: confidence interval; Cont: control; Int: intervention; N: sample size.

Study	Study Design	Pre-post, parallel or crossover	I, duration	Food environ domain	Food system domain	Behaviour change domain	Action areas, *n*	Risk of bias	Selection	Consumption	Participation rate	Attitudes
Bogart 2011	CBA	Pre-post	5 w	✓	✓	✓	6	+	▲$_{2}$			▲
Bogart 2014	C-RT	Pre-post	5 w	✓	✓	✓	6	+	◄►$_{3}$			▲
Bogart 2018	C-NRT	Pre-post	5 w	✓	✓	✓	6	+	▲$_{3}$			
Cohen 2012; 2013	C-NRT	Parallel	2 y	✓		✓	2	+	◄►$_{6}$	◄►$_{5}$		
Cullen 2015	C-RT	Parallel	15 w	✓		✓	2	+	◄►$_{5}$	◄►$_{5}$		
Elbel 2015	C-NRT	Pre-post	10 m	✓			2	+				▲
Greene 2017	C-RT	Pre-post	6 w	✓	✓	✓	5	+	▲$_{3}$	◄►$_{3}$		
Hunsberger 2015	BA	Pre-post	1 m	✓			1	+	▲$_{2}$			
Turnin 2016	BA	Pre-post	6 m	✓			1	+	▲$_{5}$			
Askelson 2019	BA	Pre-post	1 y	✓	✓	✓	5	Ø	◄►$_{3}$			▲
Bhatia 2011	BA	Pre-post	5 m	✓	✓	✓	6	Ø			▲	
Boehm 2020 1. Choices	CBA	Pre-post	4 w	✓			1	Ø	▼			
Boehm 2020 2. Nudges	CBA	Pre-post	4 w	✓		✓	3	Ø	▲			
Chu 2011 1. 66% wholewheat	NRT	Parallel	1 y	✓	✓		2	Ø				▼
Chu 2011 2. 100% wholewheat	NRT	Parallel	1 y	✓	✓		2	Ø				▼
Cullen 2007	BA	Pre-post	6 w	✓	✓	✓	3	Ø	▲$_{2}$			
Cullen 2008; Mendoza 2010	BA	Pre-post	2 y	✓			1	Ø	▲	▲$_{4}$		
D'Adamo 2021	NRT	Crossover	2 y	✓	✓	✓	3	Ø		▲		
Ellison 1989 1. Phase 1	CBA	Pre-post	6 m	✓	✓	✓	3	Ø		▲		▲
Ellison 1990 2. Phase 2	CBA	Pre-post	6 m	✓	✓	✓	3	Ø		▲		▼
Fritts 2019 1. Phase 1	NRT	Crossover	3 m	✓	✓	✓	4	Ø	◄►	▼		▼
Fritts 2019 2. Phase 2	BA	Pre-post	3 m	✓	✓	✓	4	Ø	◄►	▲		▲
Hackett 1990 1. Dish of Day	CBA	Pre-post	10 m	✓		✓	2	Ø			▼	
Hackett 1990 2. Fixed Price	CBA	Pre-post	10 m	✓		✓	3	Ø			▼	
Hanks 2012	BA	Pre-post	2 m	✓			2	Ø	◄►$_{2}$	▼$_{2}$		
Hanks 2013	BA	Pre-post	2 m	✓	✓	✓	4	Ø	▲$_{2}$	▲$_{2}$		
Just 2014	BA	Pre-post	2 d	✓	✓	✓	3	Ø	▲$_{4}$	▲$_{4}$		
Koch 2020	BA	Pre-post	1 y	✓	✓	✓	4	Ø	▼$_{5}$	◄►$_{5}$	▲	▲
Madden 2013	BA	Pre-post	1 w	✓	✓	✓	5	Ø		◄►$_{11}$		
McCool 2005 1. Phase 2 vs 1	NRT	Crossover	4 w	✓			3	Ø		▲		
McCool 2005 2. Phase 3 vs 1	NRT	Crossover	2 w	✓			3	Ø		▲		
Pope 2018	BA	Pre-post	1 m	✓	✓	✓	4	Ø	▲		▲	
Prell 2005 1. SL	CBA	Pre-post	5 w	✓	✓	✓	5	Ø		▲		
Prell 2005 2. SLHE	CBA	Pre-post	5 w	✓	✓	✓	5	Ø		▲		
Prescott 2019	CBA	Pre-post	4 w	✓	✓	✓	4	Ø	▲$_{4}$	▲$_{4}$		
Quinn 2018	C-NRT	Pre-post	1 y	✓	✓	✓	5	Ø	▲$_{3}$	◄►$_{3}$		
Schwartz 2015	BA	Pre-post	2 y	✓			1	Ø	◄►$_{4}$	▲$_{4}$		
Sharma 2018	NRT	Parallel	4 w	✓		✓	3	Ø				▲
Wansink 2013	C-NRT	Pre-post	1 m	✓			1	Ø	▲	▲		
Wansink 2015	BA	Pre-post	1 d	✓	✓	✓	4	Ø	▲	▼		
Witschi 1985	BA	Pre-post	5 w	✓	✓		2	Ø		◄►$_{2}$		▲

1. Low risk of bias:

	Selection	Consumption	Participation rate	Attitudes
Interventions reporting ED, *n*	8	3	0	3
Positive impact, *n*	5	0	0	3
Positive impact, %	63%	0%		100%
Negative impact, *n*	0	0	0	0
No change or mixed effects	3	3	0	0
Positive + negative for Sign test	5	0	0	3
Sign test^	0.063			0.250
95% CI^^	0.31, 0.86			0.44, 1.00

2. Neutral risk of bias:

	Selection	Consumption	Participation rate	Attitudes
Interventions reporting ED, *n*	17	21	5	10
Positive impact, *n*	10	14	3	6
Positive impact, %	59%	67%	60%	60%
Negative impact, *n*	2	3	2	4
No change or mixed effects	5	4	0	0
Positive + negative for Sign test	12	17	5	10
Sign test^	0.039	0.013	1.000	0.754
95% CI^^	0.36, 0.78	0.45, 0.83	0.23, 0.88	0.31, 0.83

Figure S5. Effect direction plot for sensitivity analysis: quality and risk of bias, low compared to neutral risk of bias [44,88–99,101,103–125]. **LEGEND:** Study design: C-RT, cluster randomised trial; C-NRT, cluster non-randomised trial; CBA, controlled before after study; BA, before after; NRT, non-randomised trial; studies include pre-post scores for single or multiple arm trials unless indicated as parallel arm or crossover beside study design. Study quality according to the Academy of Nutrition and Dietetics Quality Criteria Checklist [74]: denoted by row colour: green = positive rating; amber = neutral rating. Effect direction: upward arrow ▲ = positive impact, downward arrow ▼ = negative impact, sideways arrow ◄► = no change or mixed effects for multiple outcomes. Subscript numbers: Number of outcomes within each category synthesis is 1 unless indicated in subscript beside effect direction. ^Sign test: excludes studies with mixed effects direction as they cannot be said to represent either a positive or a negative effect direction [86]. ^^95% CI (confidence interval): estimation for binomial proportions using the Wilson interval method [85]. :✓: indicates the intervention has included components from the nominated NOURISHING framework domain; ED: effect direction; y: year/s; m: month/s; w: week/s; d: day/s.

Study	Study Design	Pre-post, parallel or crossover	I, duration	Food environ domain	Food system domain	Behaviour change domain	Action areas, *n*	Risk of bias	Selection	Consumption	Participation rate	Attitudes
Bogart 2014	C-RT	Pre-post	5 w	✓	✓	✓	5	+	◄►$_3$			▲
Greene 2017	C-RT	Pre-post	6 w	✓	✓	✓	5	+	▲$_3$	◄►$_3$		
Bogart 2018	C-NRT	Pre-post	5 w	✓	✓	✓	5	+	▲$_3$			
Elbel 2015	C-NRT	Pre-post	10 m	✓			2	+				▲
Quinn 2018	C-NRT	Pre-post	1 y	✓	✓	✓	5	Ø	▲$_3$	◄►$_3$		
Wansink 2013	C-NRT	Pre-post	1 m	✓			1	Ø	▲	▲		
Boehm 2020 1. Choices	CBA	Pre-post	4 w	✓			1	Ø	▼			
Boehm 2020 2. Nudges	CBA	Pre-post	4 w	✓		✓	3	Ø	▲			
Bogart 2011	CBA	Pre-post	5 w	✓	✓	✓	5	+	▲$_2$			▲
Ellison 1989 1. Phase 1	CBA	Pre-post	6 m	✓	✓	✓	3	Ø		▲		▲
Ellison 1990 2. Phase 2	CBA	Pre-post	6 m	✓	✓	✓	3	Ø		▲		▼
Hackett 1990 1. Dish of Day	CBA	Pre-post	10 m	✓		✓	2	Ø			▼	
Hackett 1990 2. Fixed Price	CBA	Pre-post	10 m	✓		✓	3	Ø			▼	
Prell 2005 1. SL	CBA	Pre-post	5 w	✓	✓	✓	5	Ø		▲		
Prell 2005 2. SLHE	CBA	Pre-post	5 w	✓	✓	✓	5	Ø		▲		
Prescott 2019	CBA	Pre-post	4 w	✓	✓	✓	4	Ø	▲$_4$	▲$_4$		
Askelson 2019	BA	Pre-post	1 y	✓	✓	✓	5	Ø	◄►$_3$			▲
Bhatia 2011	BA	Pre-post	5 m	✓	✓	✓	6	Ø			▲	
Cullen 2007	BA	Pre-post	6 w	✓	✓	✓	3	Ø	▲$_2$			
Cullen 2008; Mendoza 2010	BA	Pre-post	2 y	✓			1	Ø	▲	▲$_4$		
Fritts 2019 2. Phase 2	BA	Pre-post	3 m	✓	✓	✓	4	Ø	◄►	▲		▲
Hanks 2012	BA	Pre-post	2 m	✓			2	Ø	◄►$_2$	▼$_2$		
Hanks 2013	BA	Pre-post	2 m	✓	✓	✓	4	Ø	▲$_2$	▲$_2$		
Hunsberger 2015	BA	Pre-post	1 m	✓			1	+	▲$_2$			
Just 2014	BA	Pre-post	2 d	✓	✓	✓	3	Ø	▲$_4$	▲$_4$		
Koch 2020	BA	Pre-post	1 y	✓	✓	✓	4	Ø	▼$_5$	◄►$_5$	▲	▲
Madden 2013	BA	Pre-post	1 w	✓	✓	✓	5	Ø		◄►$_{11}$		
Pope 2018	BA	Pre-post	1 m	✓	✓	✓	4	Ø	▲		▲	
Schwartz 2015	BA	Pre-post	2 y	✓			1	Ø	◄►$_4$	▲$_4$		
Turnin 2016	BA	Pre-post	6 m	✓			1	+	▲$_5$			
Wansink 2015	BA	Pre-post	1 d	✓	✓	✓	4	Ø	▲	▼		
Witschi 1985	BA	Pre-post	5 w	✓	✓		2	Ø		◄►$_2$		▲
Chu 2011 1. 66% wholewheat	NRT	Crossover	1 y	✓	✓		2	Ø				▼
Chu 2011 2. 100% wholewheat	NRT	Crossover	1 y	✓	✓		2	Ø				▼
Sharma 2018	NRT	Parallel	4 w	✓		✓	3	Ø				▲
Cullen 2015	C-RT	Parallel	15 w	✓		✓	2	+	◄►$_5$	◄►$_5$		
Cohen 2012; 2013	C-NRT	Parallel	2 y	✓		✓	2	+	◄►$_6$	◄►$_5$		
D'Adamo 2021	NRT	Crossover	2 y	✓	✓	✓	3	Ø		▲		
Fritts 2019 1. Phase 1	NRT	Crossover	3 m	✓	✓	✓	4	Ø	◄►	▼		▼
McCool 2005 1. Phase 2 vs 1	NRT	Crossover	4 w	✓			3	Ø		▲		
McCool 2005 2. Phase 3 vs 1	NRT	Crossover	2 w	✓			3	Ø		▲		

1. Pre-post assessment:

Interventions reporting ED, *n*	22	18	5	9
Positive impact, *n*	15	11	3	8
Positive impact, %	68%	61%	60%	89%
Negative impact, *n*	2	2	2	1
No change or mixed effects	5	5	0	0
Positive + negative for Sign test	17	13	5	9
Sign test^	0.002	0.022		0.039
95% CI^^	0.47, 0.84	0.39, 0.80	0.23, 0.88	0.57, 0.98

2. Parallel or crossover assessment:

Interventions reporting ED, *n*	3	6	0	4
Positive impact, *n*	0	3	0	1
Positive impact, %	0%	50%		25%
Negative impact, *n*	0	1	0	3
No change or mixed effects	3	2	0	0
Positive + negative for Sign test	0	4	0	4
Sign test^		0.625		0.625
95% CI^^		0.19, 0.81		0.05, 0.70

Figure S6. Effect direction plot for sensitivity analysis: study design, pre-post compared to parallel or crossover assessment [44,88–99,101,103–125]. **LEGEND:** Study design: C-RT, cluster randomised trial; C-NRT, cluster non-randomised trial; CBA, controlled before after study; BA, before after; NRT, non-randomised trial; studies include pre-post scores for single or multiple arm trials unless indicated as parallel arm or crossover beside study design. Study quality according to the Academy of Nutrition and Dietetics Quality Criteria Checklist [74]: denoted by row colour: green = positive rating; amber = neutral rating. Effect direction: upward arrow ▲ = positive impact, downward arrow ▼ = negative impact, sideways arrow ◄► = no change or mixed effects for multiple outcomes. Subscript numbers: Number of outcomes within each category synthesis is 1 unless indicated in subscript beside effect direction. ^ Sign test: excludes studies with mixed effects direction as they cannot be said to represent either a positive or a negative effect direction [86]. ^^ 95% CI (confidence interval): estimation for binomial proportions using the Wilson in-terval method [85]. ✓: indicates the intervention has included components from the nominated NOURISHING framework domain; ED: effect direction.

Study	Study Design	I, duration	Food environ domain	Food system domain	Behaviour change domain	Action areas, *n*	Risk of bias	Selection	Consumption	Participation rate	Attitudes
Boehm 2020 1. Choices	CBA	4 w	✓			1	Ø	▼			
Boehm 2020 2. Nudges	CBA	4 w	✓		✓	3	Ø	▲			
Bogart 2011	CBA	5 w	✓	✓	✓	6	+	▲$_{2}$			▲
Bogart 2014	C-RT	5 w	✓	✓	✓	6	+	◀▶$_{3}$			▲
Bogart 2018	C-NRT	5 w	✓	✓	✓	6	+	▲$_{3}$			
Cullen 2007	BA	6 w	✓	✓	✓	3	Ø	▲$_{2}$			
Greene 2017	C-RT	6 w	✓	✓	✓	5	+	▲$_{3}$	◀▶$_{3}$		
Hanks 2012	BA	2 m	✓			2	Ø	◀▶$_{2}$	▼$_{2}$		
Hanks 2013	BA	2 m	✓	✓	✓	4	Ø	▲$_{2}$	▲$_{2}$		
Hunsberger 2015	BA	1 m	✓			1	+	▲$_{2}$			
Just 2014	BA	2 d	✓	✓	✓	3	Ø	▲$_{4}$	▲$_{4}$		
Madden 2013	BA	1 w	✓	✓	✓	5	Ø		◀▶$_{11}$		
McCool 2005 1. Phase 2 vs 1	NRT	4 w	✓			3	Ø		▲		
McCool 2005 2. Phase 3 vs 1	NRT	2 w	✓			3	Ø		▲		
Pope 2018	BA	1 m	✓	✓	✓	4	Ø	▲		▲	
Prell 2005 1. SL	CBA	5 w	✓	✓	✓	5	Ø		▲		
Prell 2005 2. SLHE	CBA	5 w	✓	✓	✓	5	Ø		▲		
Prescott 2019	CBA	4 w	✓	✓	✓	4	Ø	▲$_{4}$	▲$_{4}$		
Sharma 2018	NRT	4 w	✓		✓	3	Ø				▲
Wansink 2013	C-NRT	1 m	✓			1	Ø	▲	▲		
Wansink 2015	BA	1 d	✓	✓	✓	4	Ø	▲	▼		
Witschi 1985	BA	5 w	✓	✓		2	Ø		◀▶$_{2}$		▲
Bhatia 2011	BA	5 m	✓	✓	✓	6	Ø			▲	
Cullen 2015	C-RT	15 w	✓		✓	2	+	◀▶$_{5}$	◀▶$_{5}$		
Ellison 1989 1. Phase 1	CBA	6 m	✓	✓	✓	3	Ø		▲		▲
Ellison 1990 2. Phase 2	CBA	6 m	✓	✓	✓	3	Ø		▲		▼
Fritts 2019 1. Phase 1	NRT	3 m	✓	✓	✓	4	Ø	◀▶	▼		▼
Fritts 2019 2. Phase 2	BA	3 m	✓	✓	✓	4	Ø	◀▶	▲		▲
Turnin 2016	BA	6 m	✓			1	+	▲$_{5}$			
Askelson 2019	BA	1 y	✓	✓	✓	5	Ø	◀▶$_{3}$			▲
Chu 2011 1. 66% wholewheat	NRT	1 y	✓	✓		2	Ø				▼
Chu 2011 2. 100% wholewheat	NRT	1 y	✓	✓		2	Ø				▼
Cohen 2012; 2013	C-NRT	2 y	✓		✓	2	+	◀▶$_{6}$	◀▶$_{5}$		
Cullen 2008; Mendoza 2010	BA	2 y	✓			1	Ø	▲	▲$_{4}$		
D'Adamo 2021	NRT	2 y	✓	✓	✓	3	Ø		▲		
Elbel 2015	C-NRT	10 m	✓			2	+				▲
Hackett 1990 1. Dish of Day	CBA	10 m	✓		✓	2	Ø			▼	
Hackett 1990 2. Fixed Price	CBA	10 m	✓		✓	3	Ø			▼	
Koch 2020	BA	1 y	✓	✓	✓	4	Ø	▼$_{5}$	◀▶$_{5}$	▲	▲
Quinn 2018	C-NRT	1 y	✓	✓	✓	5	Ø	▲$_{3}$	◀▶$_{3}$		
Schwartz 2015	BA	2 y	✓			1	Ø	◀▶$_{4}$	▲$_{4}$		

1. Intervention duration ≤ 2 months:

	Selection	Consumption	Participation rate	Attitudes
Interventions reporting ED, *n*	15	13	1	4
Positive impact, *n*	12	8	1	4
Positive impact, %	80%	62%	100%	100%
Negative impact, *n*	1	2	0	0
No change or mixed effects	2	3	0	0
Positive + negative for Sign test	13	10	1	4
Sign test^	0.003	0.109		0.125
95% CI^^	0.55, 0.93	0.36, 0.82		0.51, 1.00

2. Intervention duration 3+ months:

	Selection	Consumption	Participation rate	Attitudes
Interventions reporting ED, *n*	10	11	4	9
Positive impact, *n*	3	6	2	5
Positive impact, %	30%	55%	50%	56%
Negative impact, *n*	1	1	2	4
No change or mixed effects	6	4	0	0
Positive + negative for Sign test	4	7	4	9
Sign test^	0.625	0.125	NA	1.000
95% CI^^	0.11, 0.60	0.28, 0.79	0.15, 0.85	0.27, 0.81

Figure S7. Effect direction plot for sensitivity analysis: intervention duration up to two months compared to interventions implemented for longer [44,88–99,101,103–125]. **LEGEND:** Study design: C-RT, cluster randomised trial; C-NRT, cluster non-randomised trial; CBA, controlled before after study; BA, before after; NRT, non-randomised trial; studies include pre-post scores for single or multiple arm trials unless indicated as parallel arm or crossover beside study design. Study quality according to the Academy of Nutrition and Dietetics Quality Criteria Checklist [74]: denoted by row colour: green = positive rating; amber = neutral rating. Effect direction: upward arrow ▲ = positive impact, downward arrow ▼ = negative impact, sideways arrow ◀▶ = no change or mixed effects for multiple outcomes. Subscript numbers: Number of outcomes within each category synthesis is 1 unless indicated in subscript beside effect direction. ^ Sign test: excludes studies with mixed effects direction as they cannot be said to represent either a positive or a negative effect direction [86]. ^^ 95% CI (confidence interval): estimation for binomial proportions using the Wilson in-terval method [85]. ✓: indicates the intervention has included components from the nominated NOURISHING framework domain; ED: effect direction.

Study	Study Design	I, duration	Food environ domain	Food system domain	Behaviour change domain	Action areas, n	Risk of bias	Selection	Consumption	Participation rate	Attitudes
Askelson 2019	BA	1 y	✓	✓	✓	5	Ø	◄►$_3$			▲
Bhatia 2011	BA	5 m	✓	✓	✓	6	Ø			▲	
Bogart 2011	CBA	5 w	✓	✓	✓	5	+	▲$_2$			▲
Bogart 2014	C-RT	5 w	✓	✓	✓	5	+	◄►$_3$			▲
Bogart 2018	C-NRT	5 w	✓	✓	✓	5	+	▲$_3$			
Cullen 2007	BA	6 w	✓	✓	✓	3	Ø	▲$_2$			
D'Adamo 2021	NRT	2 y	✓	✓	✓	3	Ø		▲		
Ellison 1989 1. Phase 1	CBA	6 m	✓	✓	✓	3	Ø		▲		▲
Ellison 1989 2. Phase 2	CBA	6 m	✓	✓	✓	3	Ø		▲		▼
Fritts 2019 1. Phase 1	NRT	3 m	✓	✓	✓	4	Ø	◄►	▼		▼
Fritts 2019 2. Phase 2	BA	3 m	✓	✓	✓	4	Ø	◄►	▲		▲
Greene 2017	C-RT	6 w	✓	✓	✓	5	+	▲$_3$	◄►$_3$		
Hanks 2013	BA	2 m	✓	✓	✓	4	Ø	▲$_2$	▲$_2$		
Just 2014	BA	2 d	✓	✓	✓	3	Ø	▲$_4$	▲$_4$		
Koch 2020	BA	1 y	✓	✓	✓	4	Ø	▼$_5$	◄►$_5$	▲	▲
Madden 2013	BA	1 w	✓	✓	✓	5	Ø		◄►$_{11}$		
Pope 2018	BA	1 m	✓	✓	✓	4	Ø	▲		▲	
Prell 2005 1. SL	CBA	5 w	✓	✓	✓	5	Ø		▲		
Prell 2005 2. SLHE	CBA	5 w	✓	✓	✓	5	Ø		▲		
Prescott 2019	CBA	4 w	✓	✓	✓	4	Ø	▲$_4$	▲$_4$		
Quinn 2018	C-NRT	1 y	✓	✓	✓	5	Ø	▲$_3$	◄►$_3$		
Wansink 2015	BA	1 d	✓	✓	✓	4	Ø	▲	▼		
Boehm 2020 2. Nudges	CBA	4 w	✓		✓	3	Ø	▲			
Chu 2011 1. 66% wholewheat	NRT	1 y	✓	✓		2	Ø				▼
Chu 2011 2. 100% wholewheat	NRT	1 y	✓	✓		2	Ø				▼
Cohen 2012; 2013	C-NRT	2 y	✓		✓	2	+	◄►$_6$	◄►$_5$		
Cullen 2015	C-RT	15 w	✓		✓	2	+	◄►$_5$	◄►$_5$		
Hackett 1990 1. Dish of Day	CBA	10 m	✓		✓	2	Ø			▼	
Hackett 1990 2. Fixed Price	CBA	10 m	✓		✓	3	Ø			▼	
Sharma 2018	NRT	4 w	✓		✓	3	Ø				▲
Witschi 1985	BA	5 w	✓	✓		2	Ø		◄►$_2$		▲
Boehm 2020 1. Choices	CBA	4 w	✓			1	Ø	▼			
Cullen 2008; Mendoza 2010	BA	2 y	✓			1	Ø	▲	▲$_4$		
Elbel 2015	C-NRT	10 m	✓			2	+				▲
Hanks 2012	BA	2 m	✓			2	Ø	◄►$_2$	▼$_2$		
Hunsberger 2015	BA	1 m	✓			1	+	▲$_2$			
McCool 2005 1. Phase 2 vs 1	NRT	4 w	✓			3	Ø		▲		
McCool 2005 2. Phase 3 vs 1	NRT	2 w	✓			3	Ø		▲		
Schwartz 2015	BA	2 y	✓			1	Ø	◄►$_4$	▲$_4$		
Turnin 2016	BA	6 m	✓			1	+	▲$_5$			
Wansink 2013	C-NRT	1 m	✓			1	Ø	▲	▲		

1. Three domains:	Selection	Consumption	Participation rate	Attitudes
Interventions reporting ED, n	15	15	3	8
Positive impact, n	10	9	3	6
Positive impact, %	67%	60%	100%	75%
Negative impact, n	1	2	0	2
No change or mixed effects	4	4	0	0
Positive + negative for Sign test	11	11	3	8
Sign test^	0.012	0.065	0.250	0.289
95% CI^^	0.42, 85	0.36, 0.80	0.44, 1.00	0.41, 0.93

2. One or two domains:	Selection	Consumption	Participation rate	Attitudes
Interventions reporting ED, n	10	9	2	5
Positive impact, n	5	5	0	3
Positive impact, %	50%	56%	0%	60%
Negative impact, n	1	1	2	2
No change or mixed effects	4	3	0	0
Positive + negative for Sign test	6	6	2	5
Sign test^	0.219	0.219	0.500	1.000
95% CI^^	0.24, 0.76	0.27, 0.81	0.00, 0.66	0.23, 0.88

Figure S8. Effect direction plot for sensitivity analysis: NOURISHING framework domains, interventions that include strategies across three domains compared to two or one domain [44,88–99,101,103–125]. **LEGEND:** Study design: C-RT, cluster randomised trial; C-NRT, cluster non-randomised trial; CBA, controlled before after study; BA, before after; NRT, non-randomised trial; studies include pre-post scores for single or multiple arm trials unless indicated as parallel arm or crossover beside study design. Study quality according to the Academy of Nutrition and Dietetics Quality Criteria Checklist [74]: denoted by row colour: green = positive rating; amber = neutral rating. Effect direction: upward arrow ▲ = positive impact, downward arrow ▼ = negative impact, sideways arrow ◄► = no change or mixed effects for multiple outcomes. Subscript numbers: Number of outcomes within each category synthesis is 1 unless indicated in subscript beside effect direction. ^ Sign test: excludes studies with mixed effects direction as they cannot be said to represent either a positive or a negative effect direction [86]. ^^ 95% CI (confidence interval): estimation for binomial proportions using the Wilson interval method [85]. ✓: indicates the intervention has included components from the nominated NOURISHING framework domain; ED: effect direction.

Study	Study Design	I, duration	Food environ domain	Food system domain	Behaviour change domain	Action areas, n	Risk of bias	Selection	Consumption	Participation rate	Attitudes
Bhatia 2011	BA	5 m	✓	✓	✓	6	Ø			▲	
Bogart 2011	CBA	5 w	✓	✓	✓	6	+	▲$_2$			▲
Bogart 2014	C-RT	5 w	✓	✓	✓	6	+	◀▶$_3$			▲
Bogart 2018	C-NRT	5 w	✓	✓	✓	6	+	▲$_3$			
Askelson 2019	BA	1 y	✓	✓	✓	5	Ø	◀▶$_3$			▲
Greene 2017	C-RT	6 w	✓	✓	✓	5	+	▲$_3$	◀▶$_3$		
Madden 2013	BA	1 w	✓	✓	✓	5	Ø		◀▶$_{11}$		
Prell 2005 1. SL	CBA	5 w	✓	✓	✓	5	Ø		▲		
Prell 2005 2. SLHE	CBA	5 w	✓	✓	✓	5	Ø		▲		
Quinn 2018	C-NRT	1 y	✓	✓	✓	5	Ø	▲$_3$	◀▶$_3$		
Fritts 2019 1. Phase 1	NRT	3 m	✓	✓	✓	4	Ø	◀▶	▼		▼
Fritts 2019 2. Phase 2	BA	3 m	✓	✓	✓	4	Ø	◀▶	▲		▲
Hanks 2013	BA	2 m	✓	✓	✓	4	Ø	▲$_2$	▲$_2$		
Koch 2020	BA	1 y	✓	✓	✓	4	Ø	▼$_5$	◀▶$_5$	▲	▲
Pope 2018	BA	1 m	✓	✓	✓	4	Ø	▲		▲	
Prescott 2019	CBA	4 w	✓	✓	✓	4	Ø	▲$_4$	▲$_4$		
Wansink 2015	BA	1 d	✓	✓	✓	4	Ø	▲	▼		
Boehm 2020 2. Nudges	CBA	4 w	✓		✓	3	Ø	▲			
Cullen 2007	BA	6 w	✓	✓	✓	3	Ø	▲$_2$			
D'Adamo 2021	NRT	2 y	✓	✓	✓	3	Ø		▲		
Ellison 1989 1. Phase 1	CBA	6 m	✓	✓	✓	3	Ø		▲		▲
Ellison 1990 2. Phase 2	CBA	6 m	✓	✓	✓	3	Ø		▲		▼
Hackett 1990 2. Fixed Price	CBA	10 m	✓		✓	3	Ø			▼	
Just 2014	BA	2 d	✓	✓	✓	3	Ø	▲$_4$	▲$_4$		
McCool 2005 1. Phase 2 vs 1	NRT	4 w	✓			3	Ø		▲		
McCool 2005 2. Phase 3 vs 1	NRT	2 w	✓			3	Ø		▲		
Sharma 2018	NRT	4 w	✓		✓	3	Ø				▲
Chu 2011 1. 66% wholewheat	NRT	1 y	✓	✓		2	Ø				▼
Chu 2011 2. 100% wholewheat	NRT	1 y	✓	✓		2	Ø				▼
Cohen 2012; 2013	C-NRT	2 y	✓		✓	2	+	◀▶$_6$	◀▶$_5$		
Cullen 2015	C-RT	15 w	✓		✓	2	+	◀▶$_5$	◀▶$_5$		
Elbel 2015	C-NRT	10 m	✓			2	+				▲
Hackett 1990 1. Dish of Day	CBA	10 m	✓		✓	2	Ø			▼	
Hanks 2012	BA	2 m	✓			2	Ø	◀▶$_2$	▼$_2$		
Witschi 1985	BA	5 w	✓	✓		2	Ø		◀▶$_2$		▲
Boehm 2020 1. Choices	CBA	4 w	✓			1	Ø	▼			
Cullen 2008 ; Mendoza 2010	BA	2 y	✓			1	Ø	▲	▲$_4$		
Hunsberger 2015	BA	1 m	✓			1	+	▲$_2$			
Schwartz 2015	BA	2 y	✓			1	Ø	◀▶$_4$	▲$_4$		
Turnin 2016	BA	6 m	✓			1	+	▲$_5$			
Wansink 2013	C-NRT	1 m	✓			1	Ø	▲	▲		

	Selection	Consumption	Participation rate	Attitudes
1. Three to six action areas:				
Interventions reporting ED, n	16	17	4	9
Positive impact, n	11	11	3	7
Positive impact, %	69%	65%	75%	78%
Negative impact, n	1	2	1	2
No change or mixed effects	4	4	0	0
Positive + negative for Sign test	12	13	4	9
Sign test^	0.006	0.022	0.625	0.180
95% CI^^	0.44, 0.86	0.41, 0.83	0.30, 0.95	0.45, 0.94
2. One or two action areas:				
Interventions reporting ED, n	9	7	1	4
Positive impact, n	4	3	0	2
Positive impact, %	44%	43%	0%	50%
Negative impact, n	1	1	1	2
No change or mixed effects	4	3	0	0
Positive + negative for Sign test	5	4	1	4
Sign test^	0.375	0.625		
95% CI^^	0.19, 0.73	0.16, 0.75		0.15, 0.85

Figure S9. Effect direction plot for sensitivity analysis: interventions targeting three or more NOURISHING framework action areas compared to interventions targeting fewer [44,88–99,101,103–125]. **LEGEND:** Study design: C-RT, cluster 37andomized trial; C-NRT, cluster non-randomised trial; CBA, controlled before after study; BA, before after; NRT, non-randomised trial; studies include pre-post scores for single or multiple arm trials unless indicated as parallel arm or crossover beside study design. Study quality according to the Academy of Nutrition and Dietetics Quality Criteria Checklist [74]: denoted by row colour: green = positive rating; amber = neutral rating. Effect direction: upward arrow ▲ = positive impact, downward arrow ▼ = negative impact, sideways arrow ◀▶ = no change or mixed effects for multiple outcomes. Subscript numbers: Number of outcomes within each category synthesis is 1 unless indicated in subscript beside effect direction. ^ Sign test: excludes studies with mixed effects direction as they cannot be said to represent either a positive or a negative effect direction [86]. ^^ 95% CI (confidence interval): estimation for binomial proportions using the Wilson interval method [85]. ◎: indicates the intervention has included components from the nominated NOURISHING framework domain; ED: effect direction.

Study	Study Design	I, duration	Food environ domain	Food system domain	Behaviour change domain	Action areas, *n*	Risk of bias	Selection	Consumption	Participation rate	Attitudes
Askelson 2019	BA	1 y	✓	✓	✓	5	Ø	◄►$_3$			▲
Bhatia 2011	BA	5 m	✓	✓	✓	6	Ø			▲	
Bogart 2011	CBA	5 w	✓	✓	✓	6	+	▲$_2$			▲
Bogart 2014	C-RT	5 w	✓	✓	✓	6	+	◄►$_3$			▲
Bogart 2018	C-NRT	5 w	✓	✓	✓	6	+	▲$_3$			
Cullen 2007	BA	6 w	✓	✓	✓	3	Ø	▲$_2$			
D'Adamo 2021	NRT	2 y	✓	✓	✓	3	Ø		▲		
Greene 2017	C-RT	6 w	✓	✓	✓	5	+	▲$_3$	◄►$_3$		
Just 2014	BA	2 d	✓	✓	✓	3	Ø	▲$_4$	▲$_4$		
Pope 2018	BA	1 m	✓	✓	✓	4	Ø	▲		▲	
Prell 2005 1. SL	CBA	5 w	✓	✓	✓	5	Ø		▲		
Prell 2005 2. SLHE	CBA	5 w	✓	✓	✓	5	Ø		▲		
Prescott 2019	CBA	4 w	✓	✓	✓	4	Ø	▲$_4$	▲$_4$		
Ellison 1989 1. Phase 1	CBA	6 m	✓	✓	✓	3	Ø		▲		▲
Ellison 1990 2. Phase 2	CBA	6 m	✓	✓	✓	3	Ø		▲		▼
Fritts 2019 1. Phase 1	NRT	3 m	✓	✓	✓	4	Ø	◄►	▼		▼
Fritts 2019 2. Phase 2	BA	3 m	✓	✓	✓	4	Ø	◄►	▲		▲
Hanks 2013	BA	2 m	✓	✓	✓	4	Ø	▲$_2$	▲$_2$		
Koch 2020	BA	1 y	✓	✓	✓	4	Ø	▼$_5$	◄►$_5$	▲	▲
Madden 2013	BA	1 w	✓	✓	✓	5	Ø		◄►$_{11}$		
Quinn 2018	C-NRT	1 y	✓	✓	✓	5	Ø	▲$_3$	◄►$_3$		
Witschi 1985	BA	5 w	✓	✓		2	Ø		◄►$_2$		▲
Boehm 2020 1. Choices	CBA	4 w	✓			1	Ø	▼			
Boehm 2020 2. Nudges	CBA	4 w	✓		✓	3	Ø	▲			
Chu 2011 1. 66% wholewheat	NRT	30 w / 1 y	✓	✓		2	Ø				▼
Chu 2011 2. 100% wholewheat	NRT	30 w / 1 y	✓	✓		2	Ø				▼
Cohen 2012; 2013	C-NRT	2 y	✓		✓	2	+	◄►$_6$	◄►$_5$		
Cullen 2008; Mendoza 2010	BA	2 y	✓			1	Ø	▲	▲$_4$		
Cullen 2015	C-RT	15 w	✓		✓	2	+	◄►$_5$	◄►$_5$		
Elbel 2015	C-NRT	10 m	✓			2	+				▲
Hackett 1990 1. Dish of Day	CBA	10 m	✓		✓	2	Ø			▼	
Hackett 1990 2. Fixed Price	CBA	10 m	✓		✓	3	Ø			▼	
Hanks 2012	BA	2 m	✓			2	Ø	◄►$_2$	▼$_2$		
Hunsberger 2015	BA	1 m	✓			1	+	▲$_2$			
McCool 2005 1. Phase 2 vs 1	NRT	4 w	✓			3	Ø		▲		
McCool 2005 2. Phase 3 vs 1	NRT	2 w	✓			3	Ø		▲		
Schwartz 2015	BA	2 y	✓			1	Ø	◄►$_4$	▲$_4$		
Sharma 2018	NRT	4 w	✓		✓	3	Ø				▲
Turnin 2016	BA	6 m	✓			1	+	▲$_5$			
Wansink 2013	C-NRT	1 m	✓			1	Ø	▲	▲		
Wansink 2015	BA	1 d	✓	✓	✓	4	Ø	▲	▼		

1. With student engagement (Askelson 2019 to Prescott 2019, *n* = 13 interventions):

	Selection	Consumption	Participation rate	Attitudes
Interventions reporting ED, *n*	9	6	2	3
Positive impact, *n*	7	5	2	3
Positive impact, %	78%	83%	100%	100%
Negative impact, *n*	0	0	0	0
No change or mixed effects	2	1	0	0
Positive + negative for Sign test	7	5	2	3
Sign test^	0.016	0.063	0.500	0.250
95% CI^^	0.45, 0.94	0.44, 0.97	0.34, 1.00	0.44, 1.00

2. Without student engagement (Ellison 1989 to Wansink 2015, *n* = 28 interventions):

	Selection	Consumption	Participation rate	Attitudes
Interventions reporting ED, *n*	16	18	3	10
Positive impact, *n*	8	9	1	6
Positive impact, %	50%	50%	33%	60%
Negative impact, *n*	2	3	2	4
No change or mixed effects	6	6	0	0
Positive + negative for Sign test	10	12	3	10
Sign test^	0.109	0.146	1.000	0.754
95% CI^^	0.28, 0.72	0.29, 0.71	0.06, 0.79	0.31, 0.83

3. With staff and/or student engagement (Askelson 2019 to Witschi 1985, *n* = 22 interventions):

	Selection	Consumption	Participation rate	Attitudes
Interventions reporting ED, *n*	14	15	3	9
Positive impact, *n*	9	9	3	7
Positive impact, %	64%	60%	100%	78%
Negative impact, *n*	1	1	0	2
No change or mixed effects	4	5	0	0
Positive + negative for Sign test	10	10	3	9
Sign test^	0.021	0.021	0.250	0.180
95% CI^^	0.39, 0.84	0.36, 0.80	0.44, 1.00	0.45, 0.94

4. Without staff and/or student engagement (Boehm 2020 to Wansink 2015, *n* = 19 interventions):

	Selection	Consumption	Participation rate	Attitudes
Interventions reporting ED, *n*	11	9	2	4
Positive impact, *n*	6	5	0	2

Figure S10. Effect direction plot for sensitivity analysis: interventions with student engagement compared to those without [44,88–99,101,103–125]. **LEGEND:** Study design: C-RT, cluster randomised trial; C-NRT, cluster non-randomised trial; CBA, controlled before after study; BA, before after; NRT, non-randomised trial; studies include pre-post scores for single or multiple arm trials unless indicated as parallel arm or crossover beside study design. Study quality according to the Academy of Nutrition and Dietetics Quality Criteria Checklist [74]: denoted by row

colour: green = positive rating; amber = neutral rating. Effect direction: upward arrow ▲ = positive impact, downward arrow ▼ = negative impact, sideways arrow ◄► = no change or mixed effects for multiple outcomes. Subscript numbers: Number of outcomes within each category synthesis is 1 unless indicated in subscript beside effect direction. ^ Sign test: excludes studies with mixed effects direction as they cannot be said to represent either a positive or a negative effect direction [86]. ^^ 95% CI (confidence interval): estimation for binomial proportions using the Wilson interval method [85]. ✓: indicates the intervention has included components from the nominated NOURISHING framework domain; ED: effect direction.

Study	Study Design	I, duration	Food environ domain	Food system domain	Behaviour change domain	Action areas, n	Risk of bias	Selection	Consumption	Participation rate	Attitudes
Askelson 2019	BA	1 y	✓	✓	✓	5	Ø	◀▶$_3$			▲
Bhatia 2011	BA	5 m	✓	✓	✓	6	Ø			▲	
Boehm 2020 2. Nudges	CBA	4 w	✓		✓	3	Ø	▲			
Bogart 2011	CBA	5 w	✓	✓	✓	6	+	▲$_2$			▲
Bogart 2014	C-RT	5 w	✓	✓	✓	6	+	◀▶$_3$			▲
Bogart 2018	C-NRT	5 w	✓	✓	✓	6	+	▲$_3$			
Cohen 2012; 2013	C-NRT	2 y	✓		✓	2	+	◀▶$_6$	◀▶$_5$		
Cullen 2007	BA	6 w	✓	✓	✓	3	Ø	▲$_2$			
Cullen 2015	C-RT	15 w	✓		✓	2	+	◀▶$_5$	◀▶$_5$		
D'Adamo 2021	NRT	2 y	✓	✓	✓	3	Ø		▲		
Ellison 1989 1. Phase 1	CBA	6 m	✓	✓	✓	3	Ø		▲		▲
Ellison 1990 2. Phase 2	CBA	6 m	✓	✓	✓	3	Ø		▲		▼
Fritts 2019 1. Phase 1	NRT	3 m	✓	✓	✓	4	Ø	◀▶	▼		▼
Fritts 2019 2. Phase 2	BA	3 m	✓	✓	✓	4	Ø	◀▶	▲		▲
Greene 2017	C-RT	6 w	✓	✓	✓	5	+	▲$_3$	◀▶$_3$		
Hackett 1990 1. Dish of Day	CBA	10 m	✓		✓	2	Ø			▼	
Hackett 1990 2. Fixed Price	CBA	10 m	✓		✓	3	Ø			▼	
Hanks 2013	BA	2 m	✓	✓	✓	4	Ø	▲$_2$	▲$_2$		
Just 2014	BA	2 d	✓	✓	✓	3	Ø	▲$_4$	▲$_4$		
Koch 2020	BA	1 y	✓	✓	✓	4	Ø	▼$_5$	◀▶$_5$	▲	▲
Madden 2013	BA	1 w	✓	✓	✓	5	Ø		◀▶$_{11}$		
Pope 2018	BA	1 m	✓	✓	✓	4	Ø	▲		▲	
Prell 2005 1. SL	CBA	5 w	✓	✓	✓	5	Ø		▲		
Prell 2005 2. SLHE	CBA	5 w	✓	✓	✓	5	Ø		▲		
Prescott 2019	CBA	4 w	✓	✓	✓	4	Ø	▲$_4$	▲$_4$		
Quinn 2018	C-NRT	1 y	✓	✓	✓	5	Ø	▲$_3$	◀▶$_3$		
Sharma 2018	NRT	4 w	✓		✓	3	Ø				▲
Wansink 2015	BA	1 d	✓	✓	✓	4	Ø	▲	▼		
Boehm 2020 1. Choices	CBA	4 w	✓			1	Ø	▼			
Chu 2011 1. 66% wholewheat	NRT	30 w / 1 y	✓	✓		2	Ø				▼
Chu 2011 2. 100% wholewheat	NRT	30 w / 1 y	✓	✓		2	Ø				▼
Cullen 2008; Mendoza 2010	BA	2 y	✓			1	Ø	▲	▲$_4$		
Elbel 2015	C-NRT	10 m	✓			2	+				▲
Hanks 2012	BA	2 m	✓			2	Ø	◀▶$_2$	▼$_2$		
Hunsberger 2015	BA	1 m	✓			1	+	▲$_2$			
McCool 2005 1. Phase 2 vs 1	NRT	4 w	✓			3	Ø		▲		
McCool 2005 2. Phase 3 vs 1	NRT	2 w	✓			3	Ø		▲		
Schwartz 2015	BA	2 y	✓			1	Ø	◀▶$_4$	▲$_4$		
Turnin 2016	BA	6 m	✓			1	+	▲$_5$			
Wansink 2013	C-NRT	1 m	✓			1	Ø	▲	▲		
Witschi 1985	BA	5 w	✓	✓		2	Ø		◀▶$_2$		▲

1. With behaviour change communication strategies (n = 28 interventions, Askelson 2019 to Wansink 2015):

	Selection	Consumption	Participation rate	Attitudes
Interventions reporting ED, n	18	17	5	9
Positive impact, n	11	9	3	7
Positive impact, %	61%	53%	60%	78%
Negative impact, n	1	2	2	2
No change or mixed effects	6	6	0	0
Positive + negative for Sign test	12	11	5	9
Sign test^	0.006	0.065	1.000	0.180
95% CI^^	0.39, 0.80	0.31, 0.74	0.23, 0.88	0.45, 0.94

2. Without behaviour change communication strategies (n = 13 interventions, Boehm Choices 2020 to Witschi 1985):

	Selection	Consumption	Participation rate	Attitudes
Interventions reporting ED, n	7	7	0	4
Positive impact, n	4	5	0	2
Positive impact, %	57%	71%		50%
Negative impact, n	1	1	0	2
No change or mixed effects	2	1	0	0
Positive + negative for Sign test	5	6	0	4
Sign test^	0.375	0.219		1.375
95% CI^^	0.25, 0.84	0.36, 0.92		0.15, 0.85

Figure S11. Effect direction plot for sensitivity analysis: interventions that include behaviour change communication compared to those without [44,88–99,101,104–118,120–125]. **LEGEND:** Study design: C-RT, cluster randomised trial; C-NRT, cluster non-randomised trial; CBA, controlled before after study; BA, before after; NRT, non-randomised trial; studies include pre-post scores for single or multiple arm trials unless indicated as parallel arm or crossover beside study design. Study quality according to the Academy of Nutrition and Dietetics Quality Criteria Checklist [74]: denoted by row colour: green = positive rating; amber = neutral rating. Effect direction: upward arrow ▲ = positive impact, downward arrow ▼ = negative impact, sideways arrow ◀▶ = no change or mixed effects for multiple outcomes. Subscript numbers: Number of outcomes within each category synthesis is 1 unless indicated in subscript beside effect direction. ^ Sign test: excludes studies with mixed effects direction as they cannot be said to represent either a positive or a negative effect direction [86]. ^^ 95% CI (confidence interval): estimation for binomial proportions using the Wilson interval method [85]. SLHE: school lunch plus home economics intervention; SL: school lunch intervention; ✓: indicates the intervention has included components from the nominated NOURISHING framework domain; ED: effect direction.
